# Supplementary material for: Coral Gardens Reef, Belize: An Acropora spp. refugium under threat in a warming world
Source: PLoS One. 2023 Feb 8;18(2):e0280852. doi: 10.1371/journal.pone.0280852 (PMC9907857; doi:10.1371/journal.pone.0280852)
Supplement: S1 Table — (PDF) [file pone.0280852.s001.pdf]

Table S1. pH measurements in proximity to Coral Gardens collected in 2019.

Date	Location	Distance from shore (m)	pH One	pH Two	Mean
7/3/2019	CARIBE ISLAND7.3	60	8.1	8.2	8.2
7/3/2019	CARIBE ISLAND7.3	75	8.2	8.2	8.2
7/3/2019	CARIBE ISLAND7.3	90	8.2	8.2	8.2
7/3/2019	EL NORTE 7.3	65	8.0	8.0	8.0
7/3/2019	EL NORTE 7.3	130	8.1	8.0	8.1
7/3/2019	EL NORTE 7.3	212	8.3	8.2	8.3
7/3/2019	EL NORTE 7.3	320	8.2	8.1	8.2
6/30/2019	CARIBE ISLAND 6.30	50	8.0	8.0	8.0
6/30/2019	CARIBE ISLAND 6.30	55	8.0	8.1	8.1
6/30/2019	CARIBE ISLAND 6.30	60	8.0	8.1	8.1
6/30/2019	CARIBE ISLAND 6.30	65	8.0	8.1	8.1
6/30/2019	CARIBE ISLAND 6.30	70	8.0	8.1	8.1
6/30/2019	CARIBE ISLAND 6.30	75	8.0	8.1	8.1
6/30/2019	CARIBE ISLAND 6.30	80	8.0	8.1	8.1
6/30/2019	CARIBE ISLAND 6.30	85	8.0	8.1	8.1
6/30/2019	CARIBE ISLAND 6.30	90	8.0	8.1	8.1
6/30/2019	CARIBE ISLAND 6.30	140	8.1	8.1	8.1
6/30/2019	CARIBE ISLAND 6.30	202	8.2	8.2	8.2
6/30/2019	AMBERGRIS DIVERS 6.30	50	8.2	8.2	8.2
6/30/2019	AMBERGRIS DIVERS 6.30	65	8.2	8.3	8.3
6/30/2019	AMBERGRIS DIVERS 6.30	80	8.2	8.2	8.2
6/30/2019	AMBERGRIS DIVERS 6.30	90	8.3	8.3	8.3
6/29/2019	CARIBE ISLAND 6.29	52	8	8	8.0
6/29/2019	CARIBE ISLAND 6.29	65	8	7.9	8.0
6/29/2019	CARIBE ISLAND 6.29	89	8	7.9	8.0
6/29/2019	CARIBE ISLAND 6.29	139	8.2	8.2	8.2
6/29/2019	CARIBE ISLAND 6.29	reef crest	8.2	8.2	8.2
6/29/2019	XANADU 6.29	64.5	8.1	8.2	8.2
6/29/2019	XANADU 6.29	80	8.1	8.1	8.1
7/2/2019	XANADU 7.2	50	7.7	7.8	7.8
7/2/2019	XANADU 7.2	65	7.8	7.7	7.8
7/2/2019	XANADU 7.2	80	7.8	7.7	7.8
