# Supplementary material for: Coral Gardens Reef, Belize: An Acropora spp. refugium under threat in a warming world
Source: PLoS One. 2023 Feb 8;18(2):e0280852. doi: 10.1371/journal.pone.0280852 (PMC9907857; doi:10.1371/journal.pone.0280852)
Supplement: S2 Table — (PDF) [file pone.0280852.s002.pdf]

Table S2. Live coral data from Coral Gardens from 2012-2019.

Transect 1 % Live Coral per quadrat each year

	**Maximum**	37.86	53.85	29.24	35.29	15.00	18.52	24.10	24.44
	**Minimum**	0.59	0.00	0	3.27	0.87	0.88	1.65	0.71
	**Mean**	19.61	19.31	14.54	13.70	6.63	8.70	9.85	12.31
	**StDev**	8.94	10.41	7.72	7.19	3.71	4.80	5.01	6.38
**Transect**	**Quadrat**	**2012**	**2013**	**2014**	**2016**	**16-Oct**	**2017**	**2018**	**2019**
1	1	26.41	28.01	26.83	19.27	4.86	3.12	3.55	7.35
1	2	27.05	22.68	20.15	35.29	3.25	4.95	3.77	4.46
1	3	11.94	14.42	6.35	15.45	15.00	5.84	4.27	8.09
1	4	16.32	22.85	10.44	10.69	9.80	3.63	12.76	12.96
1	5	26.29	22.28	28.03	15.88	9.03	4.89	6.45	4.75
1	6	21.23	16.32	14.26	24.01	13.60	15.17	24.10	21.74
1	7	19.69	17.69	16.86	14.6	8.21	10.59	15.28	17.99
1	8	23.84	53.85	17.92	12.69	11.23	9.62	8.40	9.17
1	9	29.44	33.25	21.14	11.33	5.70	12.73	12.43	17.58
1	10	20.84	28.21	29.24	29.74	11.71	12.20	15.79	22.82
1	11	10.36	9.95	10.73	20.02	10.80	18.52	13.70	17.27
1	12	12.34	16.75	14.55	14.44	7.23	7.69	11.70	12.59
1	13	18.43	22.99	10.14	13.21	10.46	9.86	11.63	15.08
1	14	10.73	19.36	10.84	15.54	6.64	12.93	11.18	18.07
1	15	13.88	18.39	4.89	8.64	6.31	16.63	11.59	22.23
1	16	11.52	29.75	6.67	17.01	8.76	8.60	14.32	21.42
1	17	10.45	34.03	25.44	13.6	7.67	15.64	21.99	24.44
1	18	11.41	9.04	10.64	15.67	8.22	10.72	11.14	14.17
1	19	19.87	0.00	0.00	14.76	3.67	11.98	9.54	11.24
1	20	23.67	0.20	0.00	4.95	2.33	16.98	9.62	9.32
1	21	30.52	15.88	4.19	4.98	0.87	10.72	9.53	13.83
1	22	24.94	18.40	14.08	11.7	2.16	13.01	12.78	12.24
1	23	28.04	12.50	18.80	11.92	6.31	7.92	9.67	15.96
1	24	30.1	10.53	17.45	3.27	2.90	5.54	6.21	17.41
1	25	34.09	13.58	20.22	6.17	1.80	3.10	3.60	2.47
1	26	2.67	10.27	10.95	8.61	3.79	5.03	3.99	0.71
1	27	0.59	21.92	21.38	14.03	3.68	7.78	7.33	6.98
1	28	17.79	23.16	16.72	14.77	3.19	0.88	8.02	13.52
1	29	10.28	14.83	11.33	5.24	4.96	1.93	1.65	8.23
1	30	13.88	24.64	20.79	3.43	3.26	9.67	7.53	2.53
1	31	37.86	12.74	9.54		8.08	4.28	5.56	11.91
1	32	32.83					4.87	11.83	4.03
1	33	19.41					7.50	9.45	12.96
1	34	18.04					1.13	4.50	10.73
1	35								4.64

Transect 2 % Live Coral per quadrat each year

	**Maximum**	65.16	59.50	58.24	56.33		37.60	40.49	42.94
	**Minimum**	0.30	1.83	2.37	0.98		1.04	0.03	0.00
	**Mean**	29.69	25.98	28.28	28.78		15.81	16.48	14.92
	**StDev**	18.17	13.31	12.95	15.82		10.12	11.91	11.82
**Transect**	**Quadrat**	**2012**	**2013**	**2014**	**2016**		**2017**	**2018**	**2019**
2	1	8.69	22.26	2.37	4.03		16.78	3.96	4.89
2	2	16.69	22.26	13.79	0.98		13.20	16.73	13.44
2	3	35.06	14.53	19.51	18.49		24.83	8.51	14.76
2	4	40.25	18.91	6.11	21.29		24.21	29.28	42.94
2	5	32.88	21.03	29.08	23.35		27.96	31.76	30.76
2	6	35.35	44.19	30.79	11.54		36.67	35.40	38.66
2	7	23.12	51.22	35.21	3.31		32.04	40.49	3.32
2	8	19.37	27.33	14.47	20.68		21.15	32.83	3.71
2	9	31.67	1.88	17.35	23.46		13.13	15.71	6.63
2	10	41.68	23.39	30.88	24.24		5.82	5.97	6.93
2	11	47.69	17.48	30.50	28.77		10.64	3.24	11.3
2	12	50.78	47.45	33.75	20.55		13.01	7.78	7.82
2	13	46.50	30.76	36.51	29.61		37.60	21.94	37.41
2	14	44.70	1.83	41.18	38.21		25.51	39.57	34.86
2	15	50.65	21.90	42.11	45.96		8.78	35.92	15.11
2	16	53.37	35.50	45.36	50.31		11.60	10.85	10.94
2	17	65.16	29.33	37.71	18.80		23.28	10.89	15.17
2	18	6.94	27.06	40.37	52.31		22.48	15.66	17.75
2	19	0.30	31.45	58.24	54.16		15.00	16.10	17.23
2	20	10.21	24.95	12.87	19.61		28.15	10.25	12.43
2	21	18.20	18.20	21.42	47.39		9.37	24.95	32.71
2	22	47.03	24.64	31.03	26.09		7.74	11.56	8.90
2	23	52.57	23.73	20.44	20.27		2.57	5.64	7.48
2	24	39.65	5.10	37.77	24.50		9.43	2.23	3.21
2	25	19.29	27.14	40.15	53.44		6.71	14.44	19.78
2	26	2.47	47.43	46.94	54.23		10.28	17.15	12.73
2	27	3.13	59.50	28.79	28.47		6.33	17.95	10.08
2	28	26.98	33.97	11.71	56.33		2.35	6.64	4.55
2	29	18.54	6.31	14.95	19.05		6.75	0.03	0.00
2	30	1.89	26.26	18.05	24.11		1.04	0.87	1.95
2	31		18.79	27.45					
2	32		25.59						

Transect 3 % Live Coral per quadrat each year

	**Maximum**	41.21	48.62	39.08	24.73		18.61	17.65	17.98
	**Minimum**	2.65	7.03	1.91	1.30		1.71	0.24	0.07
	**Mean**	24.21	23.05	21.05	13.39		6.97	4.63	5.33
	**StDev**	11.50	10.30	10.01	6.60		4.39	4.54	4.99
**Transect**	**Quadrat**	**2012**	**2013**	**2014**	**2016**		**2017**	**2018**	**2019**
3	1		7.03	1.91	4.36		3.16	1.90	1.29
3	2	14.73	10.39	9.18	1.30		6.06	1.88	5.24
3	3	22.87	13.01	6.17	8.57		4.39	4.26	2.87
3	4	27.81	22.94	14.69	4.82		4.36	2.21	4.25
3	5	22.70	15.40	23.58	10.24		1.71	3.69	0.99
3	6	23.65	28.64	15.93	5.99		7.26	0.24	6.56
3	7	41.21	36.46	25.63	10.19		5.09	3.66	2.48
3	8	33.96	31.52	23.82	8.06		4.07	2.05	0.07
3	9	39.48	27.86	39.08	15.49		6.48	1.77	1.35
3	10	33.44	48.62	27.00	24.73		1.85	1.88	0.28
3	11	26.11	28.61	36.07	6.11		3.27	0.37	0.54
3	12	32.85	23.98	22.24	19.27		5.70	1.26	4.03
3	13	2.65	12.51	15.13	13.76		7.37	4.34	9.63
3	14	5.57	17.38	9.55	12.56		7.39	3.96	7.25
3	15	11.85	18.62	16.42	12.30		4.64	5.66	2.21
3	16		22.59	22.98	16.19		13.46	2.05	4.59
3	17		29.28	25.13	20.22		14.50	8.36	13.12
3	18		32.53	33.42	19.84		18.61	9.78	17.98
3	19		10.60	32.11	18.84		10.41	17.65	11.82
3	20				22.83		12.74	10.07	13.6
3	21				22.72		3.86	14.33	1.71
3	22				16.15			0.50	

Transect 4 % Live Coral per quadrat each year

	**Maximum**	36.73	50.84	36.23	34.40		35.13	29.75	30.94
	**Minimum**	9.51	2.95244471	3.0483	2.5927		3.4421	0.8479	0.03
	**Mean**	23.73	32.68	19.39	22.52		22.27	16.45	16.07
	**StDev**	9.74	13.15	11.63	8.61		8.22	8.59	10.27
**Transect**	**Quadrat**	**2012**	**2013**	**2014**	**2016**		**2017**	**2018**	**2019**
4	1	9.51	20.52	3.54	2.59		18.64	8.14	0.03
4	2	20.32	45.99	8.51	13.24		21.75	15.77	0.47
4	3	12.02	38.24	16.65	18.86		18.77	25.10	13.76
4	4	25.94	50.84	18.79	20.46		26.02	14.50	28.86
4	5	36.57	41.36	29.35	22.70		26.65	17.21	16.88
4	6	36.73	45.92	32.77	34.11		32.25	29.75	26.46
4	7	35.20	34.56	32.38	34.40		35.13	24.04	28.98
4	8	33.70	31.98	36.23	28.32		28.56	28.93	30.94
4	9	28.05	35.84	26.68	25.14		24.58	17.11	22.64
4	10	15.36	24.41	19.37	30.17		15.07	18.92	15.68
4	11	15.57	19.55	5.38	25.89		16.36	14.94	11.10
4	12	15.79	2.95	3.05	23.17		3.44	13.98	14.93
4	13				13.76			1.08	13.60
4	14							0.85	0.70

Transect 5 % Live Coral per quadrat each year

	**Maximum**	73.85	72.33	64.33	59.89	37.50	31.21	39.22	43.85
	**Minimum**	1.62	33.60	1.28	6.03	0.54	0.49	0.70	4.78
	**Mean**	58.05	56.16	51.67	35.55	18.65	18.09	19.52	25.48
	**StDev**	12.79	11.04	12.16	13.47	8.65	7.54	9.44	9.18
**Transect**	**Quadrat**	**2012**	**2013**	**2014**	**2016**	**Oct-16**	**2017**	**2018**	**2019**
5	1	53.87	44.03	1.28	6.03	0.00	11.61	0.70	7.49
5	2	62.11	56.75	43.47	38.17	5.38	21.12	10.89	32.81
5	3	61.60	41.06	54.69	52.16	26.12	12.02	26.23	25.86
5	4	51.47	39.22	42.03	34.90	6.32	10.17	12.58	26.77
5	5	45.65	40.36	46.71	28.20	10.65	8.48	16.23	14.79
5	6	55.96	60.65	50.93	29.25	6.62	21.85	8.05	29.69
5	7	63.10	60.18	54.75	30.06	21.67	16.16	30.21	32.4
5	8	67.07	55.23	48.20	37.56	20.76	15.22	22.90	35.17
5	9	59.10	56.42	58.37	45.54	19.31	10.2	17.18	9.42
5	10	63.62	49.87	56.48	31.52	9.09	24.12	11.15	32.54
5	11	62.59	63.87	59.47	31.56	22.91	26.17	20.38	36.41
5	12	51.85	69.44	63.50	42.18	24.61	17.49	28.05	31.16
5	13	65.85	61.01	64.33	31.95	18.70	11.03	19.45	32.12
5	14	59.25	54.08	59.24	33.87	10.68	10.63	17.31	15.35
5	15	53.17	65.09	57.45	26.43	14.25	14.88	7.50	21.21
5	16	48.85	60.77	61.83	29.19	17.01	24.84	19.50	24.09
5	17	59.93	67.01	62.20	20.77	15.32	21.03	5.60	23.18
5	18	55.10	50.96	57.81	22.85	20.76	10.61	7.15	22.41
5	19	50.74	36.98	47.97	22.01	10.57	20.53	4.08	19.01
5	20	45.06	43.90	39.15	26.25	21.36	8.62	18.89	21.87
5	21	46.88	43.74	39.65	9.59	14.51	13.68	11.17	21.7
5	22	61.55	46.22	27.54	11.79	10.75	20.06	17.95	28.24
5	23	69.59	58.58	40.91	23.33	12.03	13.87	21.58	21.2
5	24	65.56	63.64	55.52	38.42	27.55	29.2	22.06	24.69
5	25	66.21	63.03	50.60	50.69	22.78	28.4	29.32	30.6
5	26	55.18	62.11	56.87	48.79	22.63	19.79	38.75	26.84
5	27	69.86	59.61	60.26	50.67	22.52	24.57	18.86	22.58
5	28	72.59	68.38	57.26	54.44	21.48	17.93	28.35	14.26
5	29	65.61	72.33	63.60	50.84	32.52	31.16	27.12	22.39
5	30	51.85	61.16	54.79	47.50	33.36	29.65	29.02	38.11
5	31	72.90	44.47	47.74	41.47	18.80	21.09	39.22	32.26
5	32	73.85	67.81	46.87	59.89	27.22	10.00	27.21	27.49
5	33	64.44	71.85	63.20	43.36	30.14	22.95	13.80	13.29
5	34	1.62	72.13	60.78	51.82	37.50	31.21	22.91	43.85
5	35		33.60	58.70	51.96	29.68	26.07	32.81	35.49
5	36			46.02	24.83	23.79	12.6	25.86	41.27
5	37					0.54	0.49	12.24	4.78
