# Supplementary material for: Coral Gardens Reef, Belize: An Acropora spp. refugium under threat in a warming world
Source: PLoS One. 2023 Feb 8;18(2):e0280852. doi: 10.1371/journal.pone.0280852 (PMC9907857; doi:10.1371/journal.pone.0280852)
Supplement: S3 Table — Satellite data were obtained as 0.5-deg gridded SST data (CoralTemp v3.1) from the Coral Reef Watch 5-km product suite (Heron et al., 2015). Maximum monthly means (MMM) SST were calculated using the gridded data for 1985–1990 + 1993 and DHW were calculated as daily values that exceeded MMM + 1°C over a 12-week period. (PDF) [file pone.0280852.s003.pdf]

	Sensor Date	Sensor Temp (°C)	Sat Date	Satellite Temp (°C)
2012	01/01/12 00:00		01/01/12	
	05/09/12 06:00		05/09/12	28.51
	05/10/12 06:00	29.15	05/10/12	28.77
	05/11/12 06:00	29.48	05/11/12	28.74
	05/12/12 06:00	29.51	05/12/12	28.38
	05/13/12 06:00	29.07	05/13/12	28.18
	05/14/12 06:00	28.95	05/14/12	28.15
	05/15/12 06:00	28.77	05/15/12	28.07
	05/16/12 06:00	28.68	05/16/12	28.21
	05/17/12 06:00	28.53	05/17/12	28.24
	05/18/12 06:00	28.47	05/18/12	28.54
	05/19/12 06:00	28.38	05/19/12	28.47
	05/20/12 06:00	28.70	05/20/12	28.63
	05/21/12 06:00	29.04	05/21/12	28.57
	05/22/12 06:00	28.26	05/22/12	28.52
	05/23/12 06:00	28.36	05/23/12	28.53
	05/24/12 06:00	28.85	05/24/12	28.90
	05/25/12 06:00	29.05	05/25/12	28.72
	05/26/12 06:00	29.05	05/26/12	28.46
	05/27/12 06:00	28.79	05/27/12	28.43
	05/28/12 06:00	28.66	05/28/12	28.25
	05/29/12 06:00		05/29/12	28.20
	05/30/12 06:00		05/30/12	28.07
	05/31/12 06:00		05/31/12	28.06
	06/01/12 06:00		06/01/12	28.25
	06/02/12 06:00		06/02/12	27.94
	06/03/12 06:00		06/03/12	28.06
	06/04/12 06:00		06/04/12	27.87
	06/05/12 06:00		06/05/12	28.15
	06/06/12 06:00		06/06/12	28.38
	06/07/12 06:00	29.15	06/07/12	28.74
	06/08/12 06:00	28.95	06/08/12	28.20
	06/09/12 06:00	28.95	06/09/12	28.34
	06/10/12 06:00	29.05	06/10/12	28.37
	06/11/12 06:00	28.85	06/11/12	28.45
	06/12/12 06:00	28.85	06/12/12	28.52
	06/13/12 06:00	28.75	06/13/12	28.45
	06/14/12 06:00	28.56	06/14/12	28.56
	06/15/12 06:00	28.66	06/15/12	28.53
	06/16/12 06:00	28.46	06/16/12	28.36
	06/17/12 06:00	28.56	06/17/12	28.32
	06/18/12 06:00	28.06	06/18/12	28.24

06/19/12 06:00	28.36	06/19/12	28.34
06/20/12 06:00	28.85	06/20/12	28.63
06/21/12 06:00	29.85	06/21/12	28.62
06/22/12 06:00	28.66	06/22/12	28.97
06/23/12 06:00	28.66	06/23/12	28.87
06/24/12 06:00	29.25	06/24/12	29.48
06/25/12 06:00	28.95	06/25/12	29.38
06/26/12 06:00	29.25	06/26/12	29.44
06/27/12 06:00	29.65	06/27/12	29.25
06/28/12 06:00	29.95	06/28/12	29.79
06/29/12 06:00	29.55	06/29/12	29.81
06/30/12 06:00	29.35	06/30/12	29.48
07/01/12 06:00	29.05	07/01/12	28.97
07/02/12 06:00	28.85	07/02/12	28.85
07/03/12 06:00	28.75	07/03/12	28.50
07/04/12 06:00	28.66	07/04/12	28.54
07/05/12 06:00	29.05	07/05/12	28.66
07/06/12 06:00	28.95	07/06/12	28.74
07/07/12 06:00	29.05	07/07/12	28.89
07/08/12 06:00	28.75	07/08/12	28.77
07/09/12 06:00	28.56	07/09/12	28.87
07/10/12 06:00	28.75	07/10/12	29.05
07/11/12 06:00	28.66	07/11/12	28.74
07/12/12 06:00	28.56	07/12/12	28.32
07/13/12 06:00	28.66	07/13/12	28.22
07/14/12 06:00	28.66	07/14/12	28.62
07/15/12 06:00	29.45	07/15/12	29.20
07/16/12 06:00	29.35	07/16/12	29.06
07/17/12 06:00	28.75	07/17/12	28.55
07/18/12 06:00	28.66	07/18/12	28.32
07/19/12 06:00	28.66	07/19/12	28.26
07/20/12 06:00	28.75	07/20/12	28.38
07/21/12 06:00	28.85	07/21/12	28.46
07/22/12 06:00	28.85	07/22/12	28.53
07/23/12 06:00	28.85	07/23/12	28.72
07/24/12 06:00	28.85	07/24/12	29.10
07/25/12 06:00	28.95	07/25/12	28.81
07/26/12 06:00	29.15	07/26/12	29.00
07/27/12 06:00	29.15	07/27/12	28.86
07/28/12 06:00	28.95	07/28/12	28.56
07/29/12 06:00	28.85	07/29/12	28.69
07/30/12 06:00	28.95	07/30/12	28.74
07/31/12 06:00	29.45	07/31/12	29.10
08/01/12 06:00	29.65	08/01/12	29.20
08/02/12 06:00	29.85	08/02/12	29.55

08/03/12 06:00	29.85	08/03/12	29.71
08/04/12 06:00	29.95	08/04/12	29.97
08/05/12 06:00	29.35	08/05/12	29.79
08/06/12 06:00	29.75	08/06/12	29.88
08/07/12 06:00	29.25	08/07/12	29.31
08/08/12 06:00	28.56	08/08/12	29.30
08/09/12 06:00	28.66	08/09/12	29.07
08/10/12 06:00	28.75	08/10/12	29.13
08/11/12 06:00	28.95	08/11/12	29.49
08/12/12 06:00	28.95	08/12/12	29.21
08/13/12 06:00	28.95	08/13/12	29.08
08/14/12 06:00	29.45	08/14/12	29.02
08/15/12 06:00	28.95	08/15/12	28.79
08/16/12 06:00	29.15	08/16/12	28.49
08/17/12 06:00	28.75	08/17/12	28.59
08/18/12 06:00	29.45	08/18/12	29.00
08/19/12 06:00	29.95	08/19/12	29.38
08/20/12 06:00	29.45	08/20/12	29.68
08/21/12 06:00	29.35	08/21/12	29.60
08/22/12 06:00	29.45	08/22/12	29.15
08/23/12 06:00	29.05	08/23/12	29.31
08/24/12 06:00	29.75	08/24/12	29.29
08/25/12 06:00	29.65	08/25/12	29.25
08/26/12 06:00	29.85	08/26/12	29.35
08/27/12 06:00	29.85	08/27/12	29.03
08/28/12 06:00	29.55	08/28/12	29.27
08/29/12 06:00	29.45	08/29/12	29.27
08/30/12 06:00	29.25	08/30/12	29.27
08/31/12 06:00	29.15	08/31/12	29.10
09/01/12 06:00	29.05	09/01/12	28.99
09/02/12 06:00	29.55	09/02/12	29.29
09/03/12 06:00	29.65	09/03/12	29.53
09/04/12 06:00	29.85	09/04/12	29.67
09/05/12 06:00	30.15	09/05/12	29.54
09/06/12 06:00	30.15	09/06/12	29.37
09/07/12 06:00	29.85	09/07/12	29.40
09/08/12 06:00	29.65	09/08/12	29.44
09/09/12 06:00	29.65	09/09/12	29.53
09/10/12 06:00	29.35	09/10/12	29.52
09/11/12 06:00	29.45	09/11/12	29.51
09/12/12 06:00	29.25	09/12/12	29.15
09/13/12 06:00	29.55	09/13/12	29.42
09/14/12 06:00	29.85	09/14/12	29.69
09/15/12 06:00	29.85	09/15/12	29.34
09/16/12 06:00	30.46	09/16/12	29.65

09/17/12 06:00	30.66	09/17/12	29.56
09/18/12 06:00	30.46	09/18/12	29.38
09/19/12 06:00	30.05	09/19/12	29.30
09/20/12 06:00	29.85	09/20/12	29.43
09/21/12 06:00	29.95	09/21/12	29.43
09/22/12 06:00	30.05	09/22/12	29.45
09/23/12 06:00	30.05	09/23/12	29.45
09/24/12 06:00	29.65	09/24/12	29.46
09/25/12 06:00	29.65	09/25/12	29.44
09/26/12 06:00	29.25	09/26/12	28.99
09/27/12 06:00	29.15	09/27/12	29.05
09/28/12 06:00	29.05	09/28/12	29.23
09/29/12 06:00	29.25	09/29/12	29.31
09/30/12 06:00	29.45	09/30/12	29.37
10/01/12 06:00	29.95	10/01/12	29.90
10/02/12 06:00	30.36	10/02/12	30.11
10/03/12 06:00	30.36	10/03/12	29.74
10/04/12 06:00	29.15	10/04/12	29.60
10/05/12 06:00	29.15	10/05/12	29.57
10/06/12 06:00	29.45	10/06/12	29.34
10/07/12 06:00	29.35	10/07/12	29.49
10/08/12 06:00	29.25	10/08/12	29.30
10/09/12 06:00	29.35	10/09/12	29.48
10/10/12 06:00	29.45	10/10/12	29.18
10/11/12 06:00	28.95	10/11/12	29.15
10/12/12 06:00	29.05	10/12/12	29.12
10/13/12 06:00	29.05	10/13/12	28.70
10/14/12 06:00	28.85	10/14/12	28.73
10/15/12 06:00	29.25	10/15/12	28.82
10/16/12 06:00	29.05	10/16/12	29.09
10/17/12 06:00	29.55	10/17/12	29.23
10/18/12 06:00	29.75	10/18/12	29.21
10/19/12 06:00	29.95	10/19/12	29.38
10/20/12 06:00	29.75	10/20/12	29.48
10/21/12 06:00	29.05	10/21/12	28.64
10/22/12 06:00	28.66	10/22/12	28.46
10/23/12 06:00	28.95	10/23/12	28.48
10/24/12 06:00	28.95	10/24/12	28.77
10/25/12 06:00	29.05	10/25/12	29.04
10/26/12 06:00	28.75	10/26/12	29.02
10/27/12 06:00	29.15	10/27/12	29.11
10/28/12 06:00	29.25	10/28/12	29.12
10/29/12 06:00	28.95	10/29/12	29.12
10/30/12 06:00	27.96	10/30/12	28.76
10/31/12 06:00	28.26	10/31/12	28.85

11/01/12 06:00	28.46	11/01/12	28.92
11/02/12 06:00	27.96	11/02/12	29.10
11/03/12 06:00	28.46	11/03/12	28.95
11/04/12 06:00	28.56	11/04/12	28.37
11/05/12 06:00	28.46	11/05/12	28.24
11/06/12 06:00	28.56	11/06/12	28.06
11/07/12 06:00	28.46	11/07/12	28.13
11/08/12 06:00	28.36	11/08/12	28.07
11/09/12 06:00	27.96	11/09/12	28.12
11/10/12 06:00	28.06	11/10/12	28.19
11/11/12 06:00	28.16	11/11/12	28.32
11/12/12 06:00	28.06	11/12/12	28.34
11/13/12 06:00	28.06	11/13/12	28.35
11/14/12 06:00	27.76	11/14/12	28.14
11/15/12 06:00	27.76	11/15/12	28.01
11/16/12 06:00	27.37	11/16/12	27.95
11/17/12 06:00	27.67	11/17/12	27.90
11/18/12 06:00	27.57	11/18/12	27.55
11/19/12 06:00	27.76	11/19/12	27.50
11/20/12 06:00	27.57	11/20/12	27.62
11/21/12 06:00	27.67	11/21/12	27.59
11/22/12 06:00	27.57	11/22/12	27.43
11/23/12 06:00	27.47	11/23/12	27.51
11/24/12 06:00	26.78	11/24/12	27.44
11/25/12 06:00	26.88	11/25/12	27.56
11/26/12 06:00	26.78	11/26/12	27.49
11/27/12 06:00	27.57	11/27/12	27.36
11/28/12 06:00	26.68	11/28/12	27.16
11/29/12 06:00	26.98	11/29/12	26.95
11/30/12 06:00	27.08	11/30/12	26.85
12/01/12 06:00	27.08	12/01/12	27.00
12/02/12 06:00	27.17	12/02/12	27.00
12/03/12 06:00	27.08	12/03/12	26.93
12/04/12 06:00	27.08	12/04/12	26.78
12/05/12 06:00	26.98	12/05/12	26.88
12/06/12 06:00	27.17	12/06/12	26.98
12/07/12 06:00	27.27	12/07/12	26.98
12/08/12 06:00	27.27	12/08/12	27.17
12/09/12 06:00	26.98	12/09/12	27.09
12/10/12 06:00	27.08	12/10/12	27.24
12/11/12 06:00	27.67	12/11/12	27.32
12/12/12 06:00	28.16	12/12/12	27.39
12/13/12 06:00	28.26	12/13/12	27.49
12/14/12 06:00	27.76	12/14/12	27.20
12/15/12 06:00	27.27	12/15/12	27.35

	12/16/12 06:00	27.37	12/16/12	27.52
	12/17/12 06:00	27.27	12/17/12	27.52
	12/18/12 06:00	27.57	12/18/12	27.55
	12/19/12 06:00	27.76	12/19/12	27.68
	12/20/12 06:00	27.27	12/20/12	27.63
	12/21/12 06:00	27.57	12/21/12	27.43
	12/22/12 06:00	26.98	12/22/12	26.61
	12/23/12 06:00	27.08	12/23/12	26.37
	12/24/12 06:00	26.68	12/24/12	26.91
	12/25/12 06:00	26.29	12/25/12	27.00
	12/26/12 06:00	26.68	12/26/12	26.61
	12/27/12 06:00	27.08	12/27/12	27.07
	12/28/12 06:00	27.17	12/28/12	27.19
	12/29/12 06:00	27.08	12/29/12	27.06
	12/30/12 06:00	27.37	12/30/12	27.26
	12/31/12 06:00	27.08	12/31/12	26.96
2013	01/01/13 06:00	27.17	01/01/13	26.86
	01/02/13 06:00	27.08	01/02/13	27.00
	01/03/13 06:00	27.27	01/03/13	26.64
	01/04/13 06:00	27.27	01/04/13	26.65
	01/05/13 06:00	27.17	01/05/13	26.67
	01/06/13 06:00	27.17	01/06/13	27.00
	01/07/13 06:00	26.98	01/07/13	26.90
	01/08/13 06:00	27.08	01/08/13	26.88
	01/09/13 06:00	27.08	01/09/13	26.83
	01/10/13 06:00	27.17	01/10/13	27.02
	01/11/13 06:00	26.88	01/11/13	26.92
	01/12/13 06:00	26.78	01/12/13	27.05
	01/13/13 06:00	26.88	01/13/13	27.20
	01/14/13 06:00	26.78	01/14/13	27.27
	01/15/13 06:00	26.88	01/15/13	27.12
	01/16/13 06:00	26.98	01/16/13	27.10
	01/17/13 06:00	27.57	01/17/13	26.93
	01/18/13 06:00	25.42	01/18/13	26.74
	01/19/13 06:00	26.29	01/19/13	26.48
	01/20/13 06:00	26.59	01/20/13	26.36
	01/21/13 06:00	26.59	01/21/13	26.42
	01/22/13 06:00	26.78	01/22/13	26.56
	01/23/13 06:00	26.29	01/23/13	26.34
	01/24/13 06:00	25.51	01/24/13	26.40
	01/25/13 06:00	25.71	01/25/13	26.22
	01/26/13 06:00	26.10	01/26/13	26.18
	01/27/13 06:00	26.10	01/27/13	26.61
	01/28/13 06:00	26.20	01/28/13	26.38
	01/29/13 06:00	26.49	01/29/13	26.70

01/30/13 06:00	26.59	01/30/13	26.81
01/31/13 06:00	26.49	01/31/13	26.70
02/01/13 06:00	26.98	02/01/13	26.66
02/02/13 06:00	26.68	02/02/13	26.49
02/03/13 06:00	26.59	02/03/13	26.32
02/04/13 06:00	26.49	02/04/13	26.20
02/05/13 06:00	26.49	02/05/13	26.25
02/06/13 06:00	26.88	02/06/13	26.68
02/07/13 06:00	27.37	02/07/13	26.76
02/08/13 06:00	27.37	02/08/13	26.92
02/09/13 06:00	27.57	02/09/13	26.90
02/10/13 06:00	26.78	02/10/13	26.89
02/11/13 06:00	26.68	02/11/13	26.94
02/12/13 06:00	26.68	02/12/13	27.04
02/13/13 06:00	26.88	02/13/13	26.95
02/14/13 06:00	26.68	02/14/13	26.99
02/15/13 06:00	27.08	02/15/13	26.81
02/16/13 06:00	27.67	02/16/13	27.03
02/17/13 06:00	26.29	02/17/13	26.62
02/18/13 06:00	26.49	02/18/13	26.30
02/19/13 06:00	26.59	02/19/13	26.21
02/20/13 06:00	26.68	02/20/13	26.22
02/21/13 06:00	26.78	02/21/13	26.61
02/22/13 06:00	26.88	02/22/13	26.92
02/23/13 06:00	26.68	02/23/13	26.97
02/24/13 06:00	26.59	02/24/13	26.54
02/25/13 06:00	26.59	02/25/13	26.49
02/26/13 06:00	26.88	02/26/13	26.89
02/27/13 06:00	27.86	02/27/13	27.11
02/28/13 06:00	27.47	02/28/13	27.23
03/01/13 06:00	28.26	03/01/13	27.17
03/02/13 06:00	25.51	03/02/13	26.80
03/03/13 06:00	25.90	03/03/13	26.10
03/04/13 06:00	26.29	03/04/13	26.19
03/05/13 06:00	26.49	03/05/13	26.22
03/06/13 06:00	26.49	03/06/13	26.21
03/07/13 06:00	26.39	03/07/13	26.25
03/08/13 06:00	26.59	03/08/13	26.34
03/09/13 06:00	25.90	03/09/13	26.23
03/10/13 06:00	26.10	03/10/13	26.09
03/11/13 06:00	26.59	03/11/13	26.04
03/12/13 06:00	26.68	03/12/13	26.32
03/13/13 06:00	25.81	03/13/13	26.34
03/14/13 06:00	25.81	03/14/13	26.19
03/15/13 06:00	25.90	03/15/13	26.00

03/16/13 06:00	26.20	03/16/13	25.88
03/17/13 06:00	26.39	03/17/13	26.04
03/18/13 06:00	26.78	03/18/13	26.45
03/19/13 06:00	26.68	03/19/13	26.62
03/20/13 06:00	26.98	03/20/13	26.63
03/21/13 06:00	26.98	03/21/13	26.85
03/22/13 06:00	27.27	03/22/13	27.17
03/23/13 06:00	27.27	03/23/13	27.58
03/24/13 06:00	27.17	03/24/13	27.38
03/25/13 06:00	27.86	03/25/13	27.43
03/26/13 06:00	27.27	03/26/13	27.28
03/27/13 06:00	26.20	03/27/13	26.53
03/28/13 06:00	26.20	03/28/13	26.27
03/29/13 06:00	26.20	03/29/13	26.10
03/30/13 06:00	26.39	03/30/13	26.30
03/31/13 06:00	26.39	03/31/13	26.53
04/01/13 06:00	26.98	04/01/13	26.64
04/02/13 06:00	27.08	04/02/13	26.53
04/03/13 06:00	27.37	04/03/13	26.69
04/04/13 06:00	27.17	04/04/13	27.32
04/05/13 06:00	27.27	04/05/13	26.70
04/06/13 06:00	26.39	04/06/13	26.66
04/07/13 06:00	26.20	04/07/13	26.49
04/08/13 06:00	26.39	04/08/13	26.53
04/09/13 06:00	26.78	04/09/13	26.66
04/10/13 06:00	26.98	04/10/13	27.21
04/11/13 06:00	26.98	04/11/13	27.21
04/12/13 06:00	26.98	04/12/13	27.50
04/13/13 06:00	27.27	04/13/13	27.70
04/14/13 06:00	27.47	04/14/13	27.58
04/15/13 06:00	27.27	04/15/13	27.63
04/16/13 06:00	27.47	04/16/13	27.53
04/17/13 06:00	27.37	04/17/13	27.58
04/18/13 06:00	27.37	04/18/13	27.46
04/19/13 06:00	27.47	04/19/13	27.54
04/20/13 06:00	27.47	04/20/13	27.40
04/21/13 06:00	28.06	04/21/13	27.51
04/22/13 06:00	27.76	04/22/13	27.70
04/23/13 06:00	28.46	04/23/13	28.12
04/24/13 06:00	28.46	04/24/13	28.23
04/25/13 06:00	28.66	04/25/13	28.09
04/26/13 06:00	28.06	04/26/13	28.03
04/27/13 06:00	28.16	04/27/13	27.77
04/28/13 06:00	27.76	04/28/13	27.69
04/29/13 06:00	27.96	04/29/13	27.99

04/30/13 06:00	27.96	04/30/13	28.03
05/01/13 06:00	28.06	05/01/13	27.94
05/02/13 06:00	28.46	05/02/13	28.04
05/03/13 06:00	28.36	05/03/13	28.10
05/04/13 06:00	28.06	05/04/13	28.06
05/05/13 06:00	27.86	05/05/13	27.56
05/06/13 06:00	27.96	05/06/13	27.34
05/07/13 06:00	28.16	05/07/13	27.61
05/08/13 06:00	27.96	05/08/13	28.08
05/09/13 06:00	28.26	05/09/13	27.96
05/10/13 06:00	28.26	05/10/13	28.03
05/11/13 06:00	28.26	05/11/13	28.20
05/12/13 06:00	28.46	05/12/13	28.17
05/13/13 06:00	28.16	05/13/13	28.18
05/14/13 06:00	28.36	05/14/13	28.09
05/15/13 06:00	28.46	05/15/13	28.34
05/16/13 06:00	28.56	05/16/13	28.51
05/17/13 06:00	28.56	05/17/13	28.53
05/18/13 06:00	28.36	05/18/13	28.42
05/19/13 06:00	28.66	05/19/13	28.39
05/20/13 06:00	28.66	05/20/13	28.16
05/21/13 06:00	28.56	05/21/13	28.35
05/22/13 06:00	28.56	05/22/13	28.21
05/23/13 06:00	28.56	05/23/13	28.24
05/24/13 06:00	28.56	05/24/13	28.26
05/25/13 06:00		05/25/13	27.93
05/26/13 06:00		05/26/13	27.96
05/27/13 06:00		05/27/13	27.52
05/28/13 06:00	27.88	05/28/13	27.70
05/29/13 06:00	27.75	05/29/13	27.69
05/30/13 06:00	28.20	05/30/13	28.39
05/31/13 06:00	28.44	05/31/13	28.50
06/01/13 06:00	28.53	06/01/13	28.23
06/02/13 06:00	28.56	06/02/13	28.34
06/03/13 06:00	28.05	06/03/13	28.23
06/04/13 06:00	28.30	06/04/13	28.38
06/05/13 06:00	28.57	06/05/13	28.48
06/06/13 06:00	28.75	06/06/13	28.76
06/07/13 06:25	29.15	06/07/13	29.15
06/08/13 06:25	29.15	06/08/13	28.78
06/09/13 06:25	28.95	06/09/13	28.63
06/10/13 06:25	28.75	06/10/13	28.62
06/11/13 06:25	28.95	06/11/13	28.94
06/12/13 06:25	28.95	06/12/13	28.87
06/13/13 06:25	28.90	06/13/13	28.96

06/14/13 06:25	29.00	06/14/13	28.85
06/15/13 06:25	29.10	06/15/13	28.84
06/16/13 06:25	29.05	06/16/13	28.82
06/17/13 06:25	28.85	06/17/13	28.77
06/18/13 06:25	28.56	06/18/13	28.56
06/19/13 06:25	28.46	06/19/13	28.21
06/20/13 06:25	28.46	06/20/13	28.13
06/21/13 06:25	28.31	06/21/13	28.05
06/22/13 06:25	28.56	06/22/13	28.15
06/23/13 06:25	28.51	06/23/13	28.20
06/24/13 06:25	28.46	06/24/13	28.33
06/25/13 06:25	29.50	06/25/13	28.59
06/26/13 06:25	28.85	06/26/13	28.56
06/27/13 06:25	29.50	06/27/13	28.68
06/28/13 06:25	29.30	06/28/13	28.56
06/29/13 06:25	29.45	06/29/13	28.63
06/30/13 06:25	29.55	06/30/13	29.53
07/01/13 06:25	30.00	07/01/13	29.54
07/02/13 06:25	29.40	07/02/13	29.24
07/03/13 06:25	29.05	07/03/13	29.06
07/04/13 06:25	29.05	07/04/13	28.40
07/05/13 06:25	28.51	07/05/13	28.52
07/06/13 06:25	28.85	07/06/13	28.65
07/07/13 06:25	28.85	07/07/13	28.65
07/08/13 06:25	29.05	07/08/13	28.96
07/09/13 06:25	29.90	07/09/13	29.15
07/10/13 06:25	29.35	07/10/13	28.85
07/11/13 06:25	28.90	07/11/13	28.67
07/12/13 06:25	28.75	07/12/13	28.76
07/13/13 06:25	28.95	07/13/13	28.51
07/14/13 06:25	28.56	07/14/13	28.36
07/15/13 06:25	28.41	07/15/13	28.35
07/16/13 06:25	28.26	07/16/13	28.39
07/17/13 06:25	28.56	07/17/13	28.25
07/18/13 06:25	28.85	07/18/13	28.13
07/19/13 06:25	28.61	07/19/13	28.59
07/20/13 06:25	28.75	07/20/13	28.69
07/21/13 06:25	28.75	07/21/13	28.74
07/22/13 06:25	28.66	07/22/13	28.64
07/23/13 06:25	28.85	07/23/13	28.77
07/24/13 06:25	28.75	07/24/13	28.64
07/25/13 06:25	28.85	07/25/13	28.62
07/26/13 06:25	29.05	07/26/13	28.63
07/27/13 06:25	29.45	07/27/13	28.84
07/28/13 06:25	29.50	07/28/13	29.35

07/29/13 06:25	29.25	07/29/13	29.44
07/30/13 06:25	29.15	07/30/13	29.34
07/31/13 06:25	29.60	07/31/13	29.35
08/01/13 06:25	29.85	08/01/13	29.43
08/02/13 06:25	29.65	08/02/13	29.68
08/03/13 06:25	29.45	08/03/13	29.37
08/04/13 06:25	29.15	08/04/13	28.79
08/05/13 06:25	29.15	08/05/13	28.36
08/06/13 06:25	29.25	08/06/13	28.60
08/07/13 06:25	29.95	08/07/13	29.22
08/08/13 06:25	30.36	08/08/13	29.38
08/09/13 06:25	30.26	08/09/13	29.54
08/10/13 06:25	30.05	08/10/13	29.56
08/11/13 06:25	29.50	08/11/13	29.38
08/12/13 06:25	29.10	08/12/13	28.84
08/13/13 06:25	29.35	08/13/13	28.88
08/14/13 06:25	29.50	08/14/13	28.85
08/15/13 06:25	29.45	08/15/13	28.59
08/16/13 06:25	29.15	08/16/13	28.40
08/17/13 06:25	29.15	08/17/13	28.79
08/18/13 06:25	28.95	08/18/13	28.92
08/19/13 06:25	29.85	08/19/13	29.40
08/20/13 06:25	29.60	08/20/13	29.44
08/21/13 06:25	30.05	08/21/13	28.91
08/22/13 06:25	30.15	08/22/13	29.07
08/23/13 06:25	30.15	08/23/13	29.16
08/24/13 06:25	29.40	08/24/13	28.35
08/25/13 06:25	29.00	08/25/13	28.24
08/26/13 06:25	29.05	08/26/13	28.43
08/27/13 06:25	29.70	08/27/13	28.43
08/28/13 06:25	29.80	08/28/13	28.59
08/29/13 06:25	29.70	08/29/13	28.62
08/30/13 06:25	29.20	08/30/13	28.77
08/31/13 06:25	29.05	08/31/13	28.57
09/01/13 06:25	28.95	09/01/13	28.62
09/02/13 06:25	29.60	09/02/13	28.88
09/03/13 06:25	29.00	09/03/13	28.83
09/04/13 06:25	28.56	09/04/13	29.04
09/05/13 06:25	29.20	09/05/13	28.52
09/06/13 06:25	29.75	09/06/13	28.66
09/07/13 06:25	29.95	09/07/13	29.08
09/08/13 06:25	29.55	09/08/13	29.22
09/09/13 06:25	29.95	09/09/13	28.93
09/10/13 06:25	29.90	09/10/13	29.10
09/11/13 06:25	28.70	09/11/13	28.95

09/12/13 06:25	28.56	09/12/13	28.99
09/13/13 06:25	28.66	09/13/13	29.14
09/14/13 06:25	28.66	09/14/13	28.92
09/15/13 06:25	28.70	09/15/13	29.00
09/16/13 06:25	28.85	09/16/13	28.90
09/17/13 06:25	28.56	09/17/13	28.87
09/18/13 06:25	27.96	09/18/13	28.96
09/19/13 06:25	28.66	09/19/13	28.88
09/20/13 06:25	28.66	09/20/13	28.82
09/21/13 06:25	29.20	09/21/13	29.22
09/22/13 06:25	29.85	09/22/13	29.34
09/23/13 06:25	30.41	09/23/13	29.59
09/24/13 06:25	30.36	09/24/13	29.50
09/25/13 06:25	29.80	09/25/13	29.16
09/26/13 06:25	29.55	09/26/13	29.55
09/27/13 06:25	29.35	09/27/13	29.81
09/28/13 06:25	29.85	09/28/13	29.94
09/29/13 06:25	30.26	09/29/13	30.01
09/30/13 06:25	30.15	09/30/13	29.97
10/01/13 06:25	29.75	10/01/13	29.85
10/02/13 06:25	30.66	10/02/13	29.80
10/03/13 06:25	30.66	10/03/13	29.56
10/04/13 06:25	29.45	10/04/13	29.44
10/05/13 06:25	29.95	10/05/13	29.61
10/06/13 06:25	29.95	10/06/13	29.53
10/07/13 06:25	30.05	10/07/13	29.37
10/08/13 06:25	29.65	10/08/13	29.07
10/09/13 06:25	29.80	10/09/13	29.11
10/10/13 06:25	28.51	10/10/13	29.08
10/11/13 06:25	29.10	10/11/13	29.08
10/12/13 06:25	29.35	10/12/13	29.18
10/13/13 06:25	29.55	10/13/13	29.27
10/14/13 06:25	29.45	10/14/13	29.14
10/15/13 06:25	29.15	10/15/13	29.01
10/16/13 06:25	29.40	10/16/13	29.12
10/17/13 06:25	29.30	10/17/13	29.25
10/18/13 06:25	29.60	10/18/13	29.27
10/19/13 06:25	29.50	10/19/13	29.36
10/20/13 06:25	29.90	10/20/13	29.34
10/21/13 06:25	29.80	10/21/13	29.44
10/22/13 06:25	29.20	10/22/13	29.50
10/23/13 06:25	29.75	10/23/13	29.51
10/24/13 06:25	29.65	10/24/13	29.24
10/25/13 06:25	29.15	10/25/13	28.92
10/26/13 06:25	29.25	10/26/13	28.75

10/27/13 06:25	29.10	10/27/13	28.61
10/28/13 06:25	28.95	10/28/13	28.54
10/29/13 06:25	28.95	10/29/13	28.85
10/30/13 06:25	28.95	10/30/13	28.85
10/31/13 06:25	29.00	10/31/13	28.78
11/01/13 06:25	28.85	11/01/13	28.89
11/02/13 06:25	29.55	11/02/13	28.70
11/03/13 06:25	30.15	11/03/13	28.88
11/04/13 06:25	28.75	11/04/13	28.63
11/05/13 06:25	29.55	11/05/13	28.54
11/06/13 06:25	29.45	11/06/13	28.64
11/07/13 06:25	29.10	11/07/13	28.48
11/08/13 06:25	29.00	11/08/13	28.38
11/09/13 06:25	28.75	11/09/13	28.15
11/10/13 06:25	29.00	11/10/13	28.23
11/11/13 06:25	28.36	11/11/13	27.92
11/12/13 06:25	28.46	11/12/13	28.10
11/13/13 06:25	28.70	11/13/13	28.10
11/14/13 06:25	28.36	11/14/13	28.06
11/15/13 06:25	28.51	11/15/13	27.92
11/16/13 06:25	28.16	11/16/13	27.85
11/17/13 06:25	28.26	11/17/13	28.13
11/18/13 06:25	28.75	11/18/13	28.18
11/19/13 06:25	29.20	11/19/13	28.47
11/20/13 06:25	28.85	11/20/13	28.73
11/21/13 06:25	28.90	11/21/13	28.55
11/22/13 06:25	28.75	11/22/13	28.40
11/23/13 06:25	28.36	11/23/13	28.22
11/24/13 06:25	28.36	11/24/13	28.15
11/25/13 06:25	28.46	11/25/13	28.12
11/26/13 06:25	28.75	11/26/13	27.88
11/27/13 06:25	28.46	11/27/13	27.65
11/28/13 06:25	28.56	11/28/13	27.75
11/29/13 06:25	28.06	11/29/13	27.46
11/30/13 06:25	28.26	11/30/13	27.78
12/01/13 06:25	28.11	12/01/13	27.58
12/02/13 06:25	27.67	12/02/13	27.58
12/03/13 06:25	28.06	12/03/13	27.67
12/04/13 06:25	28.01	12/04/13	27.87
12/05/13 06:25	28.11	12/05/13	27.89
12/06/13 06:25	27.86	12/06/13	27.88
12/07/13 06:25	28.21	12/07/13	27.81
12/08/13 06:25	28.16	12/08/13	27.69
12/09/13 06:25	28.26	12/09/13	27.55
12/10/13 06:25	28.26	12/10/13	27.93

	12/11/13 06:25	28.21	12/11/13	27.87
	12/12/13 06:25	28.16	12/12/13	27.55
	12/13/13 06:25	28.06	12/13/13	27.54
	12/14/13 06:25	27.67	12/14/13	27.62
	12/15/13 06:25	27.86	12/15/13	27.46
	12/16/13 06:25	27.71	12/16/13	27.29
	12/17/13 06:25	26.93	12/17/13	27.14
	12/18/13 06:25	27.67	12/18/13	27.26
	12/19/13 06:25	28.01	12/19/13	27.26
	12/20/13 06:25	27.76	12/20/13	27.39
	12/21/13 06:25	27.76	12/21/13	27.56
	12/22/13 06:25	27.96	12/22/13	27.81
	12/23/13 06:25	27.67	12/23/13	27.59
	12/24/13 06:25	27.81	12/24/13	27.60
	12/25/13 06:25	27.76	12/25/13	27.33
	12/26/13 06:25	27.86	12/26/13	27.14
	12/27/13 06:25	27.71	12/27/13	27.18
	12/28/13 06:25	27.71	12/28/13	27.16
	12/29/13 06:25	27.76	12/29/13	27.13
	12/30/13 06:25	27.03	12/30/13	27.14
	12/31/13 06:25	27.52	12/31/13	27.31
2014	01/01/14 06:25	27.47	01/01/14	27.46
	01/02/14 06:25	27.47	01/02/14	27.61
	01/03/14 06:25	27.17	01/03/14	27.33
	01/04/14 06:25	27.12	01/04/14	27.03
	01/05/14 06:25	27.12	01/05/14	27.08
	01/06/14 06:25	27.57	01/06/14	27.08
	01/07/14 06:25	26.88	01/07/14	26.92
	01/08/14 06:25	26.98	01/08/14	26.69
	01/09/14 06:25	27.27	01/09/14	26.57
	01/10/14 06:25	26.83	01/10/14	26.66
	01/11/14 06:25	26.78	01/11/14	26.79
	01/12/14 06:25	26.93	01/12/14	26.86
	01/13/14 06:25	26.63	01/13/14	26.78
	01/14/14 06:25	27.52	01/14/14	26.86
	01/15/14 06:25	26.73	01/15/14	26.73
	01/16/14 06:25	24.64	01/16/14	26.44
	01/17/14 06:25	25.71	01/17/14	26.47
	01/18/14 06:25	25.85	01/18/14	26.21
	01/19/14 06:25	26.59	01/19/14	26.20
	01/20/14 06:25	26.34	01/20/14	26.22
	01/21/14 06:25	26.54	01/21/14	26.33
	01/22/14 06:25	26.34	01/22/14	26.56
	01/23/14 06:25	26.59	01/23/14	26.22
	01/24/14 06:25	26.49	01/24/14	26.15

01/25/14 06:25	26.44	01/25/14	26.27
01/26/14 06:25	26.49	01/26/14	26.32
01/27/14 06:25	26.39	01/27/14	26.17
01/28/14 06:25	26.44	01/28/14	26.47
01/29/14 06:25	26.88	01/29/14	26.57
01/30/14 06:25	27.08	01/30/14	26.44
01/31/14 06:25	26.10	01/31/14	26.30
02/01/14 06:25	26.10	02/01/14	26.21
02/02/14 06:25	26.24	02/02/14	26.49
02/03/14 06:25	26.39	02/03/14	26.68
02/04/14 06:25	26.59	02/04/14	26.76
02/05/14 06:25	26.59	02/05/14	26.92
02/06/14 06:25	26.59	02/06/14	26.78
02/07/14 06:25	26.54	02/07/14	26.91
02/08/14 06:25	27.42	02/08/14	26.95
02/09/14 06:25	27.27	02/09/14	26.88
02/10/14 06:25	26.73	02/10/14	26.93
02/11/14 06:25	26.73	02/11/14	26.98
02/12/14 06:25	26.68	02/12/14	26.96
02/13/14 06:25	27.52	02/13/14	26.98
02/14/14 06:25	26.44	02/14/14	26.88
02/15/14 06:25	26.78	02/15/14	27.03
02/16/14 06:25	27.17	02/16/14	27.04
02/17/14 06:25	26.54	02/17/14	26.75
02/18/14 06:25	26.59	02/18/14	26.66
02/19/14 06:25	26.78	02/19/14	26.73
02/20/14 06:25	26.98	02/20/14	26.95
02/21/14 06:25	27.03	02/21/14	27.11
02/22/14 06:25	26.98	02/22/14	27.03
02/23/14 06:25	26.98	02/23/14	27.06
02/24/14 06:25	27.03	02/24/14	27.03
02/25/14 06:25	27.03	02/25/14	26.95
02/26/14 06:25	27.67	02/26/14	27.20
02/27/14 06:25	27.32	02/27/14	27.31
02/28/14 06:25	27.57	02/28/14	27.50
03/01/14 06:25	27.86	03/01/14	27.63
03/02/14 06:25	27.67	03/02/14	27.42
03/03/14 06:25	27.12	03/03/14	27.12
03/04/14 06:25	27.03	03/04/14	27.07
03/05/14 06:25	27.42	03/05/14	27.22
03/06/14 06:25	27.57	03/06/14	27.16
03/07/14 06:25	27.42	03/07/14	27.01
03/08/14 06:25	27.27	03/08/14	27.01
03/09/14 06:25	27.27	03/09/14	27.08
03/10/14 06:25	27.22	03/10/14	27.09

03/11/14 06:25	27.91	03/11/14	27.43
03/12/14 06:25	27.91	03/12/14	27.56
03/13/14 06:25	28.31	03/13/14	27.89
03/14/14 06:25	27.22	03/14/14	27.38
03/15/14 06:25	27.32	03/15/14	27.46
03/16/14 06:25	27.27	03/16/14	27.59
03/17/14 06:25	27.47	03/17/14	27.62
03/18/14 06:25	27.27	03/18/14	27.08
03/19/14 06:25	27.17	03/19/14	26.97
03/20/14 06:25	27.47	03/20/14	27.09
03/21/14 06:25	27.47	03/21/14	27.33
03/22/14 06:25	27.91	03/22/14	27.64
03/23/14 06:25	27.91	03/23/14	27.91
03/24/14 06:25	27.86	03/24/14	27.70
03/25/14 06:25	28.31	03/25/14	27.58
03/26/14 06:25	28.61	03/26/14	27.92
03/27/14 06:25	27.76	03/27/14	27.68
03/28/14 06:25	27.86	03/28/14	27.72
03/29/14 06:25	27.52	03/29/14	27.71
03/30/14 06:25	28.21	03/30/14	27.39
03/31/14 06:25	28.51	03/31/14	27.75
04/01/14 06:25	28.06	04/01/14	27.70
04/02/14 06:25	28.06	04/02/14	27.52
04/03/14 06:25	28.11	04/03/14	27.24
04/04/14 06:25	28.06	04/04/14	27.28
04/05/14 06:25	28.16	04/05/14	27.36
04/06/14 06:25	28.16	04/06/14	27.39
04/07/14 06:25	28.11	04/07/14	27.48
04/08/14 06:25	28.16	04/08/14	27.60
04/09/14 06:25	27.32	04/09/14	27.23
04/10/14 06:25	27.32	04/10/14	27.13
04/11/14 06:25	27.52	04/11/14	27.16
04/12/14 06:25	27.47	04/12/14	27.07
04/13/14 06:25	27.47	04/13/14	27.47
04/14/14 06:25	27.62	04/14/14	27.73
04/15/14 06:25	27.57	04/15/14	27.50
04/16/14 06:25	28.11	04/16/14	28.06
04/17/14 06:25	28.06	04/17/14	27.80
04/18/14 06:25	27.96	04/18/14	27.82
04/19/14 06:25	28.26	04/19/14	27.92
04/20/14 06:25	28.26	04/20/14	28.35
04/21/14 06:25	28.26	04/21/14	28.14
04/22/14 06:25	28.16	04/22/14	28.18
04/23/14 06:25	28.26	04/23/14	28.36
04/24/14 06:25	28.21	04/24/14	28.41

04/25/14 06:25	28.56	04/25/14	28.26
04/26/14 06:25	28.26	04/26/14	28.19
04/27/14 06:25	28.11	04/27/14	28.24
04/28/14 06:25	28.26	04/28/14	28.26
04/29/14 06:25	28.26	04/29/14	28.12
04/30/14 06:25	28.26	04/30/14	27.73
05/01/14 06:25	28.16	05/01/14	27.71
05/02/14 06:25	28.16	05/02/14	27.99
05/03/14 06:25	28.36	05/03/14	28.01
05/04/14 06:25	27.86	05/04/14	28.15
05/05/14 06:25	28.21	05/05/14	28.21
05/06/14 06:25	28.46	05/06/14	28.49
05/07/14 06:25	28.66	05/07/14	28.46
05/08/14 06:25	28.46	05/08/14	28.24
05/09/14 06:25	28.41	05/09/14	28.21
05/10/14 06:25	28.46	05/10/14	28.06
05/11/14 06:25	28.36	05/11/14	28.11
05/12/14 06:25	28.21	05/12/14	27.94
05/13/14 06:25	27.96	05/13/14	27.98
05/14/14 06:25	28.16	05/14/14	28.10
05/15/14 06:25	29.00	05/15/14	28.11
05/16/14 06:25	28.70	05/16/14	28.12
05/17/14 06:25	27.91	05/17/14	28.11
05/18/14 06:25	27.91	05/18/14	28.24
05/19/14 06:25	28.51	05/19/14	28.28
05/20/14 06:25	28.46	05/20/14	28.42
05/21/14 06:25	28.66	05/21/14	28.31
05/22/14 06:25	28.46	05/22/14	28.52
05/23/14 06:25	28.46	05/23/14	28.46
05/24/14 06:25	28.36	05/24/14	28.47
05/25/14 06:25	28.36	05/25/14	28.50
05/26/14 06:25	28.31	05/26/14	28.45
05/27/14 06:25	28.26	05/27/14	28.17
05/28/14 06:25	28.06	05/28/14	27.89
05/29/14 06:25	28.26	05/29/14	28.19
05/30/14 06:25	28.46	05/30/14	28.24
05/31/14 06:25	28.46	05/31/14	28.33
06/01/14 06:25	28.46	06/01/14	28.22
06/02/14 06:25	28.36	06/02/14	27.93
06/03/14 06:25	28.46	06/03/14	27.92
06/04/14 06:25	28.46	06/04/14	27.77
06/05/14 06:25	28.36	06/05/14	27.83
06/06/14 06:25	28.36	06/06/14	27.71
06/07/14 06:25	28.36	06/07/14	27.50
06/08/14 06:25	28.16	06/08/14	27.46

06/09/14 06:25	28.16	06/09/14	27.39
06/10/14 06:25	28.36	06/10/14	27.62
06/11/14 06:25	28.46	06/11/14	27.67
06/12/14 06:25	28.56	06/12/14	28.10
06/13/14 06:25	28.56	06/13/14	28.41
06/14/14 06:25	28.80	06/14/14	28.46
06/15/14 06:25	28.36	06/15/14	28.50
06/16/14 06:25	28.31	06/16/14	28.47
06/17/14 06:25	28.56	06/17/14	28.42
06/18/14 06:25	28.56	06/18/14	28.44
06/19/14 06:25	28.56	06/19/14	28.54
06/20/14 06:25	28.66	06/20/14	28.47
06/21/14 06:25	28.56	06/21/14	28.40
06/22/14 06:25	28.46	06/22/14	28.52
06/23/14 06:25	28.46	06/23/14	28.60
06/24/14 06:25	28.46	06/24/14	28.73
06/25/14 06:25	28.46	06/25/14	28.61
06/26/14 06:25	28.51	06/26/14	28.66
06/27/14 06:25	28.46	06/27/14	28.50
06/28/14 06:25	28.56	06/28/14	28.04
06/29/14 06:25	28.36	06/29/14	28.17
06/30/14 06:25	28.70	06/30/14	28.30
07/01/14 06:00	28.85	07/01/14	28.59
07/02/14 06:00	28.90	07/02/14	28.77
07/03/14 06:00	28.95	07/03/14	28.67
07/04/14 06:00	26.44	07/04/14	28.53
07/05/14 06:00	26.56	07/05/14	28.54
07/06/14 06:00	25.13	07/06/14	28.48
07/07/14 06:00	28.70	07/07/14	28.56
07/08/14 06:00	28.88	07/08/14	28.45
07/09/14 06:00	28.85	07/09/14	28.41
07/10/14 06:00	28.88	07/10/14	28.50
07/11/14 06:00	29.13	07/11/14	28.90
07/12/14 06:00	28.95	07/12/14	28.88
07/13/14 06:00	29.00	07/13/14	28.88
07/14/14 06:00	28.51	07/14/14	28.64
07/15/14 06:00	28.75	07/15/14	28.75
07/16/14 06:00	29.30	07/16/14	28.83
07/17/14 06:00	29.65	07/17/14	28.90
07/18/14 06:00	29.33	07/18/14	29.16
07/19/14 06:00	29.35	07/19/14	29.26
07/20/14 06:00	29.05	07/20/14	29.23
07/21/14 06:00	29.35	07/21/14	29.46
07/22/14 06:00	29.35	07/22/14	29.21
07/23/14 06:00	29.35	07/23/14	29.07

07/24/14 06:00	29.43	07/24/14	29.15
07/25/14 06:00	29.40	07/25/14	29.13
07/26/14 06:00	29.53	07/26/14	29.07
07/27/14 06:00	29.55	07/27/14	29.29
07/28/14 06:00	29.38	07/28/14	29.03
07/29/14 06:00	29.35	07/29/14	28.97
07/30/14 06:00	29.30	07/30/14	28.99
07/31/14 06:00	30.36	07/31/14	29.24
08/01/14 06:00	29.75	08/01/14	29.36
08/02/14 06:00	29.85	08/02/14	29.42
08/03/14 06:00	30.56	08/03/14	29.55
08/04/14 06:00	30.46	08/04/14	29.78
08/05/14 06:00	30.05	08/05/14	29.79
08/06/14 06:00	29.83	08/06/14	29.87
08/07/14 06:00	29.95	08/07/14	29.78
08/08/14 06:00	29.95	08/08/14	30.01
08/09/14 06:00	30.05	08/09/14	29.56
08/10/14 06:00	29.68	08/10/14	29.59
08/11/14 06:00	29.85	08/11/14	29.44
08/12/14 06:00	29.85	08/12/14	29.39
08/13/14 06:00	29.85	08/13/14	29.52
08/14/14 06:00	30.20	08/14/14	29.67
08/15/14 06:00	29.70	08/15/14	29.49
08/16/14 06:00	29.68	08/16/14	29.30
08/17/14 06:00	29.65	08/17/14	29.12
08/18/14 06:00	29.85	08/18/14	29.09
08/19/14 06:00	29.90	08/19/14	29.16
08/20/14 06:00	29.75	08/20/14	28.98
08/21/14 06:00	29.55	08/21/14	28.75
08/22/14 06:00	29.95	08/22/14	29.13
08/23/14 06:00	29.95	08/23/14	29.56
08/24/14 06:00	30.28	08/24/14	29.99
08/25/14 06:00	30.13	08/25/14	30.06
08/26/14 06:00	30.00	08/26/14	30.13
08/27/14 06:00	30.61	08/27/14	30.15
08/28/14 06:00	30.86	08/28/14	30.16
08/29/14 06:00	29.80	08/29/14	30.22
08/30/14 06:00	29.70	08/30/14	29.91
08/31/14 06:00	29.65	08/31/14	29.51
09/01/14 06:00	29.45	09/01/14	29.44
09/02/14 06:00	29.35	09/02/14	29.07
09/03/14 06:00	29.15	09/03/14	28.89
09/04/14 06:00	29.68	09/04/14	29.15
09/05/14 06:00	29.65	09/05/14	29.05
09/06/14 06:00	29.48	09/06/14	28.87

09/07/14 06:00	29.30	09/07/14	28.71
09/08/14 06:00	29.33	09/08/14	28.72
09/09/14 06:00	29.43	09/09/14	28.93
09/10/14 06:00	30.05	09/10/14	29.08
09/11/14 06:00	29.35	09/11/14	29.10
09/12/14 06:00	28.90	09/12/14	29.20
09/13/14 06:00	28.88	09/13/14	29.21
09/14/14 06:00	28.95	09/14/14	29.08
09/15/14 06:00	29.05	09/15/14	29.15
09/16/14 06:00	29.05	09/16/14	29.33
09/17/14 06:00	29.25	09/17/14	29.42
09/18/14 06:00	29.20	09/18/14	29.73
09/19/14 06:00	29.33	09/19/14	29.81
09/20/14 06:00	29.28	09/20/14	29.77
09/21/14 06:00	29.55	09/21/14	29.44
09/22/14 06:00	29.40	09/22/14	29.43
09/23/14 06:00	29.80	09/23/14	29.30
09/24/14 06:00	29.40	09/24/14	29.24
09/25/14 06:00	28.90	09/25/14	29.21
09/26/14 06:00	28.68	09/26/14	28.83
09/27/14 06:00	28.83	09/27/14	28.79
09/28/14 06:00	29.18	09/28/14	29.12
09/29/14 06:00	29.65	09/29/14	29.21
09/30/14 06:00	29.95	09/30/14	29.27
10/01/14 06:00	29.93	10/01/14	29.31
10/02/14 06:00	29.68	10/02/14	29.30
10/03/14 06:00	29.90	10/03/14	29.28
10/04/14 06:00	29.75	10/04/14	29.29
10/05/14 06:00	29.68	10/05/14	29.40
10/06/14 06:00	29.75	10/06/14	29.41
10/07/14 06:00	29.70	10/07/14	29.36
10/08/14 06:00	29.40	10/08/14	29.13
10/09/14 06:00	28.73	10/09/14	29.04
10/10/14 06:00	28.68	10/10/14	29.10
10/11/14 06:00	29.55	10/11/14	28.81
10/12/14 06:00	29.05	10/12/14	28.88
10/13/14 06:00	29.05	10/13/14	28.89
10/14/14 06:00	29.13	10/14/14	28.93
10/15/14 06:00	29.15	10/15/14	28.61
10/16/14 06:00	28.83	10/16/14	28.39
10/17/14 06:00	28.85	10/17/14	28.57
10/18/14 06:00	28.85	10/18/14	28.50
10/19/14 06:00	28.36	10/19/14	28.51
10/20/14 06:00	28.46	10/20/14	28.32
10/21/14 06:00	28.63	10/21/14	28.50

10/22/14 06:00	28.80	10/22/14	28.42
10/23/14 06:00	29.48	10/23/14	28.61
10/24/14 06:00	29.40	10/24/14	28.58
10/25/14 06:00	28.41	10/25/14	28.31
10/26/14 06:00	28.04	10/26/14	28.42
10/27/14 06:00	28.36	10/27/14	28.48
10/28/14 06:00	28.36	10/28/14	28.40
10/29/14 06:00	28.48	10/29/14	28.37
10/30/14 06:00	28.28	10/30/14	28.27
10/31/14 06:00	28.66	10/31/14	28.07
11/01/14 06:00	28.53	11/01/14	27.90
11/02/14 06:00	28.46	11/02/14	27.80
11/03/14 06:00	28.26	11/03/14	27.84
11/04/14 06:00	28.36	11/04/14	27.78
11/05/14 06:00	28.26	11/05/14	27.96
11/06/14 06:00	28.08	11/06/14	28.09
11/07/14 06:00	27.81	11/07/14	28.23
11/08/14 06:00	28.31	11/08/14	28.09
11/09/14 06:00	27.99	11/09/14	27.94
11/10/14 06:00	26.93	11/10/14	27.66
11/11/14 06:00	27.76	11/11/14	27.44
11/12/14 06:00	27.81	11/12/14	27.72
11/13/14 06:00	27.84	11/13/14	27.91
11/14/14 06:00	27.91	11/14/14	27.93
11/15/14 06:00	27.96	11/15/14	27.78
11/16/14 06:00	28.06	11/16/14	28.20
11/17/14 06:00	27.96	11/17/14	28.29
11/18/14 06:00	28.28	11/18/14	28.27
11/19/14 06:00	27.76	11/19/14	28.14
11/20/14 06:00	28.06	11/20/14	27.66
11/21/14 06:00	27.99	11/21/14	27.61
11/22/14 06:00	27.76	11/22/14	27.74
11/23/14 06:00	28.06	11/23/14	27.64
11/24/14 06:00	28.11	11/24/14	27.75
11/25/14 06:00	28.36	11/25/14	27.78
11/26/14 06:00	28.46	11/26/14	27.71
11/27/14 06:00	26.46	11/27/14	27.47
11/28/14 06:00	27.35	11/28/14	27.26
11/29/14 06:00	27.47	11/29/14	27.27
11/30/14 06:00	27.57	11/30/14	27.56
12/01/14 06:00	27.57	12/01/14	27.41
12/02/14 06:00	27.37	12/02/14	27.41
12/03/14 06:00	27.25	12/03/14	27.26
12/04/14 06:00	27.25	12/04/14	27.11
12/05/14 06:00	27.08	12/05/14	27.18

	12/06/14 06:00	26.90	12/06/14	27.16
	12/07/14 06:00	27.08	12/07/14	27.26
	12/08/14 06:00	27.08	12/08/14	27.24
	12/09/14 06:00	26.76	12/09/14	27.04
	12/10/14 06:00	26.78	12/10/14	26.85
	12/11/14 06:00	26.78	12/11/14	26.88
	12/12/14 06:00	27.20	12/12/14	26.72
	12/13/14 06:00	27.10	12/13/14	26.48
	12/14/14 06:00	27.27	12/14/14	26.22
	12/15/14 06:00	27.39	12/15/14	26.39
	12/16/14 06:00	27.37	12/16/14	26.68
	12/17/14 06:00	27.27	12/17/14	26.74
	12/18/14 06:00	27.37	12/18/14	26.91
	12/19/14 06:00	27.47	12/19/14	26.90
	12/20/14 06:00	27.27	12/20/14	26.69
	12/21/14 06:00	27.47	12/21/14	26.99
	12/22/14 06:00	27.49	12/22/14	27.29
	12/23/14 06:00	27.00	12/23/14	27.29
	12/24/14 06:00	26.98	12/24/14	27.18
	12/25/14 06:00	26.68	12/25/14	27.15
	12/26/14 06:00	26.63	12/26/14	27.00
	12/27/14 06:00	27.00	12/27/14	26.71
	12/28/14 06:00	27.10	12/28/14	26.55
	12/29/14 06:00	27.17	12/29/14	26.70
	12/30/14 06:00	27.27	12/30/14	27.02
	12/31/14 06:00	27.27	12/31/14	26.94
2015	01/01/15 06:00	27.17	01/01/15	26.96
	01/02/15 06:00	27.17	01/02/15	26.88
	01/03/15 06:00	27.08	01/03/15	26.71
	01/04/15 06:00	27.08	01/04/15	26.83
	01/05/15 06:00	26.78	01/05/15	26.92
	01/06/15 06:00	26.78	01/06/15	26.70
	01/07/15 06:00	26.68	01/07/15	26.65
	01/08/15 06:00	26.68	01/08/15	26.58
	01/09/15 06:00	26.83	01/09/15	26.49
	01/10/15 06:00	26.61	01/10/15	26.59
	01/11/15 06:00	26.88	01/11/15	26.42
	01/12/15 06:00	26.85	01/12/15	26.60
	01/13/15 06:00	26.90	01/13/15	26.58
	01/14/15 06:00	26.98	01/14/15	26.70
	01/15/15 06:00	26.98	01/15/15	26.88
	01/16/15 06:00	27.17	01/16/15	27.03
	01/17/15 06:00	27.17	01/17/15	26.86
	01/18/15 06:00	27.08	01/18/15	26.50
	01/19/15 06:00	26.85	01/19/15	26.44

01/20/15 06:00	26.44	01/20/15	26.45
01/21/15 06:00	26.78	01/21/15	26.69
01/22/15 06:00	26.93	01/22/15	26.73
01/23/15 06:00	26.78	01/23/15	27.00
01/24/15 06:00	27.35	01/24/15	27.11
01/25/15 06:00	25.34	01/25/15	26.82
01/26/15 06:00	26.39	01/26/15	26.43
01/27/15 06:00	26.59	01/27/15	26.74
01/28/15 06:00	26.78	01/28/15	26.75
01/29/15 06:00	26.88	01/29/15	26.50
01/30/15 06:00	26.85	01/30/15	26.38
01/31/15 06:00	26.88	01/31/15	26.54
02/01/15 06:00	26.78	02/01/15	26.59
02/02/15 06:00	26.66	02/02/15	26.58
02/03/15 06:00	26.85	02/03/15	26.53
02/04/15 06:00	27.10	02/04/15	26.56
02/05/15 06:00	27.08	02/05/15	26.60
02/06/15 06:00	26.46	02/06/15	26.43
02/07/15 06:00	25.90	02/07/15	26.32
02/08/15 06:00	26.34	02/08/15	26.23
02/09/15 06:00	26.68	02/09/15	26.33
02/10/15 06:00	26.51	02/10/15	26.28
02/11/15 06:00	26.27	02/11/15	26.16
02/12/15 06:00	26.68	02/12/15	26.28
02/13/15 06:00	26.78	02/13/15	26.33
02/14/15 06:00	26.68	02/14/15	26.17
02/15/15 06:00	26.39	02/15/15	26.04
02/16/15 06:00	26.39	02/16/15	25.99
02/17/15 06:00	26.39	02/17/15	26.22
02/18/15 06:00	27.10	02/18/15	26.45
02/19/15 06:00	25.76	02/19/15	26.04
02/20/15 06:00	26.29	02/20/15	25.77
02/21/15 06:00	25.90	02/21/15	25.76
02/22/15 06:00	26.10	02/22/15	25.98
02/23/15 06:00	26.29	02/23/15	26.19
02/24/15 06:00	26.49	02/24/15	26.31
02/25/15 06:00	26.88	02/25/15	26.50
02/26/15 06:00	26.98	02/26/15	26.78
02/27/15 06:00	27.37	02/27/15	27.15
02/28/15 06:00	27.20	02/28/15	26.90
03/01/15 06:00	27.08	03/01/15	26.87
03/02/15 06:00	26.98	03/02/15	26.74
03/03/15 06:00	27.03	03/03/15	26.81
03/04/15 06:00	27.00	03/04/15	26.84
03/05/15 06:00	27.03	03/05/15	26.85

03/06/15 06:00	27.00	03/06/15	26.71
03/07/15 06:00	27.67	03/07/15	26.67
03/08/15 06:00	26.85	03/08/15	26.74
03/09/15 06:00	26.88	03/09/15	26.56
03/10/15 06:00	26.90	03/10/15	26.66
03/11/15 06:00	27.03	03/11/15	26.75
03/12/15 06:00	27.08	03/12/15	26.88
03/13/15 06:00	27.17	03/13/15	26.96
03/14/15 06:00	27.27	03/14/15	26.91
03/15/15 06:00	27.30	03/15/15	26.98
03/16/15 06:00	27.15	03/16/15	26.62
03/17/15 06:00	27.17	03/17/15	26.50
03/18/15 06:00	27.27	03/18/15	26.55
03/19/15 06:00	27.37	03/19/15	26.81
03/20/15 06:00	27.08	03/20/15	26.95
03/21/15 06:00	27.03	03/21/15	27.26
03/22/15 06:00	27.22	03/22/15	27.23
03/23/15 06:00	27.37	03/23/15	27.12
03/24/15 06:00	27.96	03/24/15	27.65
03/25/15 06:00	28.26	03/25/15	27.90
03/26/15 06:00	27.91	03/26/15	27.82
03/27/15 06:00	27.67	03/27/15	27.47
03/28/15 06:00	27.89	03/28/15	27.29
03/29/15 06:00	27.37	03/29/15	27.06
03/30/15 06:00	27.37	03/30/15	26.82
03/31/15 06:00	27.27	03/31/15	26.89
04/01/15 06:00		04/01/15	26.83
04/02/15 06:00	27.17	04/02/15	26.69
04/03/15 06:00	27.37	04/03/15	27.04
04/04/15 06:00	27.22	04/04/15	27.20
04/05/15 06:00	27.27	04/05/15	27.40
04/06/15 06:00	27.30	04/06/15	27.29
04/07/15 06:00	27.11	04/07/15	27.30
04/08/15 06:00	27.17	04/08/15	27.32
04/09/15 06:00	27.37	04/09/15	27.38
04/10/15 06:00	27.57	04/10/15	27.46
04/11/15 06:00	27.63	04/11/15	27.52
04/12/15 06:00	27.70	04/12/15	27.59
04/13/15 06:00	27.83	04/13/15	27.49
04/14/15 06:00	27.73	04/14/15	27.34
04/15/15 06:00	27.76	04/15/15	27.43
04/16/15 06:00	27.67	04/16/15	27.63
04/17/15 06:00	27.73	04/17/15	27.66
04/18/15 06:00	27.83	04/18/15	27.64
04/19/15 06:00	27.86	04/19/15	27.93

04/20/15 06:00	27.99	04/20/15	28.01
04/21/15 06:00	28.06	04/21/15	28.19
04/22/15 06:00	28.16	04/22/15	27.83
04/23/15 06:00	28.16	04/23/15	27.44
04/24/15 06:00	28.13	04/24/15	27.68
04/25/15 06:00	28.62	04/25/15	28.22
04/26/15 06:00	28.19	04/26/15	28.54
04/27/15 06:00	28.16	04/27/15	28.24
04/28/15 06:00	28.46	04/28/15	27.94
04/29/15 06:00	29.42	04/29/15	27.83
04/30/15 06:00	28.03	04/30/15	27.81
05/01/15 06:00	27.53	05/01/15	27.59
05/02/15 06:00	28.09	05/02/15	27.61
05/03/15 06:00	27.73	05/03/15	27.50
05/04/15 06:00	27.96	05/04/15	27.87
05/05/15 06:00	28.52	05/05/15	28.34
05/06/15 06:00	28.56	05/06/15	28.53
05/07/15 06:00	28.42	05/07/15	28.42
05/08/15 06:00	28.62	05/08/15	28.24
05/09/15 06:00	28.46	05/09/15	28.10
05/10/15 06:00	28.42	05/10/15	27.80
05/11/15 06:00	28.36	05/11/15	27.77
05/12/15 06:00	28.36	05/12/15	27.82
05/13/15 06:00	28.16	05/13/15	27.86
05/14/15 06:00	28.29	05/14/15	27.78
05/15/15 06:00	28.32	05/15/15	27.79
05/16/15 06:00	28.26	05/16/15	27.97
05/17/15 06:00	28.26	05/17/15	28.08
05/18/15 06:00	28.06	05/18/15	27.94
05/19/15 06:00	28.26	05/19/15	27.99
05/20/15 06:00	28.66	05/20/15	28.46
05/21/15 06:00	28.85	05/21/15	28.62
05/22/15 06:00	29.02	05/22/15	28.60
05/23/15 06:00	28.62	05/23/15	28.52
05/24/15 06:00	28.42	05/24/15	28.27
05/25/15 06:00	28.36	05/25/15	28.24
05/26/15 06:00	28.42	05/26/15	28.07
05/27/15 06:00	28.42	05/27/15	28.00
05/28/15 06:00	28.32	05/28/15	28.13
05/29/15 06:00	28.85	05/29/15	28.31
05/30/15 06:00	28.66	05/30/15	28.25
05/31/15 06:00	28.66	05/31/15	28.21
06/01/15 06:00	28.36	06/01/15	28.26
06/02/15 06:00	28.13	06/02/15	28.25
06/03/15 06:00	28.26	06/03/15	28.30

06/04/15 06:00	28.46	06/04/15	28.68
06/05/15 06:00	29.42	06/05/15	28.86
06/06/15 06:00	29.42	06/06/15	28.94
06/07/15 06:00	29.75	06/07/15	28.93
06/08/15 06:00	29.89	06/08/15	28.93
06/09/15 06:00	28.95	06/09/15	28.72
06/10/15 06:00	28.82	06/10/15	28.60
06/11/15 06:00	28.72	06/11/15	28.58
06/12/15 06:00	28.66	06/12/15	28.65
06/13/15 06:00	28.16	06/13/15	28.71
06/14/15 06:00		06/14/15	28.45
06/15/15 06:00	28.56	06/15/15	28.34
06/16/15 06:00	28.46	06/16/15	28.24
06/17/15 06:00	28.36	06/17/15	28.31
06/18/15 06:00	28.46	06/18/15	28.14
06/19/15 06:00	28.46	06/19/15	27.93
06/20/15 06:00	28.46	06/20/15	27.80
06/21/15 06:00	28.36	06/21/15	27.61
06/22/15 06:00	28.66	06/22/15	27.80
06/23/15 06:00	28.70	06/23/15	28.12
06/24/15 06:00	28.85	06/24/15	28.45
06/25/15 06:00	28.56	06/25/15	28.19
06/26/15 06:00	28.11	06/26/15	27.94
06/27/15 06:00	28.16	06/27/15	27.88
06/28/15 06:00	28.06	06/28/15	27.87
06/29/15 06:00	28.26	06/29/15	28.14
06/30/15 06:00	28.66	06/30/15	27.96
07/01/15 06:00	28.26	07/01/15	27.94
07/02/15 06:00	28.46	07/02/15	27.97
07/03/15 06:00	28.36	07/03/15	27.69
07/04/15 06:00	28.36	07/04/15	27.81
07/05/15 06:00	28.66	07/05/15	28.14
07/06/15 06:00	28.66	07/06/15	28.27
07/07/15 06:00	28.61	07/07/15	28.16
07/08/15 06:00	28.85	07/08/15	28.32
07/09/15 06:00	28.75	07/09/15	27.99
07/10/15 06:00	28.66	07/10/15	27.88
07/11/15 06:00	28.51	07/11/15	28.10
07/12/15 06:00	28.75	07/12/15	27.90
07/13/15 06:00	29.25	07/13/15	28.73
07/14/15 06:00	29.20	07/14/15	28.87
07/15/15 06:00	29.05	07/15/15	29.09
07/16/15 06:00	28.75	07/16/15	28.63
07/17/15 06:00	28.61	07/17/15	28.29
07/18/15 06:00	28.66	07/18/15	28.51

07/19/15 06:00	28.85	07/19/15	28.59
07/20/15 06:00	28.75	07/20/15	28.50
07/21/15 06:00	28.61	07/21/15	28.59
07/22/15 06:00	28.56	07/22/15	28.65
07/23/15 06:00	28.75	07/23/15	28.55
07/24/15 06:00	28.95	07/24/15	28.34
07/25/15 06:00	29.05	07/25/15	28.34
07/26/15 06:00	28.95	07/26/15	28.89
07/27/15 06:00	29.20	07/27/15	28.89
07/28/15 06:00	29.20	07/28/15	28.87
07/29/15 06:00	29.15	07/29/15	28.63
07/30/15 06:00	29.05	07/30/15	28.82
07/31/15 06:00	28.90	07/31/15	28.66
08/01/15 06:00	29.70	08/01/15	28.96
08/02/15 06:00	29.60	08/02/15	29.20
08/03/15 06:00	29.25	08/03/15	28.89
08/04/15 06:00	29.05	08/04/15	29.04
08/05/15 06:00	29.40	08/05/15	29.11
08/06/15 06:00	29.25	08/06/15	28.79
08/07/15 06:00	29.50	08/07/15	28.94
08/08/15 06:00	29.60	08/08/15	29.05
08/09/15 06:00	29.35	08/09/15	29.39
08/10/15 06:00	29.55	08/10/15	29.31
08/11/15 06:00	29.45	08/11/15	29.54
08/12/15 06:00	29.40	08/12/15	29.22
08/13/15 06:00	29.25	08/13/15	29.32
08/14/15 06:00	29.55	08/14/15	29.33
08/15/15 06:00	29.85	08/15/15	29.59
08/16/15 06:00	29.65	08/16/15	29.30
08/17/15 06:00	29.75	08/17/15	29.47
08/18/15 06:00	29.65	08/18/15	29.41
08/19/15 06:00	29.55	08/19/15	29.43
08/20/15 06:00	29.55	08/20/15	29.45
08/21/15 06:00	29.75	08/21/15	29.72
08/22/15 06:00	30.20	08/22/15	29.88
08/23/15 06:00	30.41	08/23/15	29.92
08/24/15 06:00	30.36	08/24/15	30.00
08/25/15 06:00	31.06	08/25/15	29.67
08/26/15 06:00	30.66	08/26/15	29.70
08/27/15 06:00	30.56	08/27/15	30.08
08/28/15 06:00	30.96	08/28/15	29.84
08/29/15 06:00	30.61	08/29/15	29.73
08/30/15 06:00	31.06	08/30/15	29.91
08/31/15 06:00	31.06	08/31/15	30.27
09/01/15 06:00	30.56	09/01/15	30.25

09/02/15 06:00	30.46	09/02/15	30.10
09/03/15 06:00	30.36	09/03/15	29.84
09/04/15 06:00	30.36	09/04/15	29.91
09/05/15 06:00	30.46	09/05/15	30.10
09/06/15 06:00	30.41	09/06/15	30.02
09/07/15 06:00	29.70	09/07/15	29.77
09/08/15 06:00	30.31	09/08/15	29.55
09/09/15 06:00	29.70	09/09/15	29.30
09/10/15 06:00	29.90	09/10/15	29.67
09/11/15 06:00	29.90	09/11/15	29.41
09/12/15 06:00	29.65	09/12/15	29.35
09/13/15 06:00	29.90	09/13/15	29.63
09/14/15 06:00	29.85	09/14/15	29.64
09/15/15 06:00	29.90	09/15/15	29.57
09/16/15 06:00	30.41	09/16/15	29.68
09/17/15 06:00	30.71	09/17/15	29.71
09/18/15 06:00	30.86	09/18/15	29.81
09/19/15 06:00	30.96	09/19/15	29.61
09/20/15 06:00	30.96	09/20/15	29.66
09/21/15 06:00	29.35	09/21/15	29.70
09/22/15 06:00	28.90	09/22/15	29.57
09/23/15 06:00	29.50	09/23/15	29.54
09/24/15 06:00	29.65	09/24/15	29.48
09/25/15 06:00	29.55	09/25/15	29.57
09/26/15 06:00	28.31	09/26/15	29.54
09/27/15 06:00	28.26	09/27/15	29.48
09/28/15 06:00	29.45	09/28/15	29.30
09/29/15 06:00	29.75	09/29/15	29.59
09/30/15 06:00	30.36	09/30/15	29.53
10/01/15 06:00	30.61	10/01/15	29.60
10/02/15 06:00	30.61	10/02/15	29.75
10/03/15 06:00	29.35	10/03/15	29.52
10/04/15 06:00	29.55	10/04/15	29.39
10/05/15 06:00	29.60	10/05/15	29.30
10/06/15 06:00	29.65	10/06/15	29.44
10/07/15 06:00	29.65	10/07/15	29.47
10/08/15 06:00	29.60	10/08/15	29.68
10/09/15 06:00	29.55	10/09/15	29.66
10/10/15 06:00	30.20	10/10/15	29.70
10/11/15 06:00	30.05	10/11/15	29.77
10/12/15 06:00	30.51	10/12/15	30.07
10/13/15 06:00	30.56	10/13/15	30.16
10/14/15 06:00	30.46	10/14/15	30.01
10/15/15 06:00	29.65	10/15/15	29.61
10/16/15 06:00	29.40	10/16/15	29.40

10/17/15 06:00	29.35	10/17/15	29.18
10/18/15 06:00	28.75	10/18/15	28.99
10/19/15 06:00	27.37	10/19/15	28.92
10/20/15 06:00	28.66	10/20/15	29.02
10/21/15 06:00	28.66	10/21/15	28.82
10/22/15 06:00	28.61	10/22/15	28.64
10/23/15 06:00	29.15	10/23/15	28.89
10/24/15 06:00	29.35	10/24/15	29.00
10/25/15 06:00	29.65	10/25/15	29.06
10/26/15 06:00	29.40	10/26/15	28.85
10/27/15 06:00	29.55	10/27/15	29.23
10/28/15 06:00	29.90	10/28/15	29.31
10/29/15 06:00	30.15	10/29/15	29.63
10/30/15 06:00	30.05	10/30/15	29.41
10/31/15 06:00	29.80	10/31/15	29.41
11/01/15 06:00	29.55	11/01/15	29.36
11/02/15 06:00	29.45	11/02/15	29.27
11/03/15 06:00	29.65	11/03/15	29.21
11/04/15 06:00	29.50	11/04/15	29.24
11/05/15 06:00	29.10	11/05/15	29.18
11/06/15 06:00	29.25	11/06/15	28.89
11/07/15 06:00	28.95	11/07/15	29.08
11/08/15 06:00	29.45	11/08/15	28.99
11/09/15 06:00	29.60	11/09/15	29.05
11/10/15 06:00	29.30	11/10/15	29.12
11/11/15 06:00	29.15	11/11/15	28.94
11/12/15 06:00	28.95	11/12/15	28.63
11/13/15 06:00	28.80	11/13/15	28.61
11/14/15 06:00	28.75	11/14/15	28.56
11/15/15 06:00	28.51	11/15/15	28.15
11/16/15 06:00	28.66	11/16/15	28.12
11/17/15 06:00	28.66	11/17/15	28.10
11/18/15 06:00	28.80	11/18/15	28.22
11/19/15 06:00	28.90	11/19/15	28.56
11/20/15 06:00	28.95	11/20/15	28.72
11/21/15 06:00	28.85	11/21/15	28.48
11/22/15 06:00	28.80	11/22/15	28.35
11/23/15 06:00	29.00	11/23/15	28.60
11/24/15 06:00	29.15	11/24/15	28.68
11/25/15 06:00	28.80	11/25/15	28.58
11/26/15 06:00	28.61	11/26/15	28.22
11/27/15 06:00	28.21	11/27/15	28.02
11/28/15 06:00	28.46	11/28/15	28.02
11/29/15 06:00	28.16	11/29/15	28.16
11/30/15 06:00	28.31	11/30/15	27.93

	12/01/15 06:00	28.56	12/01/15	27.70
	12/02/15 06:00	28.70	12/02/15	27.94
	12/03/15 06:00	28.85	12/03/15	28.14
	12/04/15 06:00	28.21	12/04/15	27.85
	12/05/15 06:00	28.16	12/05/15	27.57
	12/06/15 06:00	28.46	12/06/15	27.44
	12/07/15 06:00	27.96	12/07/15	27.42
	12/08/15 06:00	28.01	12/08/15	27.45
	12/09/15 06:00	28.31	12/09/15	27.38
	12/10/15 06:00	28.46	12/10/15	27.45
	12/11/15 06:00	28.06	12/11/15	27.74
	12/12/15 06:00	28.16	12/12/15	27.91
	12/13/15 06:00	28.06	12/13/15	28.14
	12/14/15 06:00	27.96	12/14/15	28.06
	12/15/15 06:00	28.31	12/15/15	28.03
	12/16/15 06:00	28.21	12/16/15	28.08
	12/17/15 06:00	28.41	12/17/15	28.17
	12/18/15 06:00	28.61	12/18/15	28.12
	12/19/15 06:00	28.66	12/19/15	27.94
	12/20/15 06:00	28.26	12/20/15	27.76
	12/21/15 06:00	28.26	12/21/15	27.69
	12/22/15 06:00	28.11	12/22/15	27.79
	12/23/15 06:00	28.01	12/23/15	27.83
	12/24/15 06:00	28.11	12/24/15	27.95
	12/25/15 06:00	28.16	12/25/15	27.81
	12/26/15 06:00	27.91	12/26/15	27.79
	12/27/15 06:00	27.96	12/27/15	27.81
	12/28/15 06:00	28.06	12/28/15	27.81
	12/29/15 06:00	27.96	12/29/15	27.69
	12/30/15 06:00	28.06	12/30/15	27.75
	12/31/15 06:00	28.16	12/31/15	27.72
2016	01/01/16 06:00	27.76	01/01/16	27.82
	01/02/16 06:00	28.06	01/02/16	27.87
	01/03/16 06:00	28.01	01/03/16	27.85
	01/04/16 06:00	27.52	01/04/16	27.65
	01/05/16 06:00	27.71	01/05/16	27.25
	01/06/16 06:00	27.76	01/06/16	27.38
	01/07/16 06:00	27.62	01/07/16	27.21
	01/08/16 06:00	27.76	01/08/16	27.31
	01/09/16 06:00	28.21	01/09/16	27.51
	01/10/16 06:00	28.26	01/10/16	27.58
	01/11/16 06:00	27.17	01/11/16	27.18
	01/12/16 06:00	27.42	01/12/16	27.30
	01/13/16 06:00	28.06	01/13/16	27.29
	01/14/16 06:00	28.16	01/14/16	27.44

01/15/16 06:00	27.91	01/15/16	27.66
01/16/16 06:00	28.26	01/16/16	28.02
01/17/16 06:00	28.61	01/17/16	28.05
01/18/16 06:00	27.67	01/18/16	27.58
01/19/16 06:00	27.67	01/19/16	27.26
01/20/16 06:00	27.67	01/20/16	27.17
01/21/16 06:00	27.67	01/21/16	27.31
01/22/16 06:00	27.37	01/22/16	27.23
01/23/16 06:00	25.76	01/23/16	26.75
01/24/16 06:00	25.85	01/24/16	26.86
01/25/16 06:00	26.63	01/25/16	27.00
01/26/16 06:00	26.63	01/26/16	27.00
01/27/16 06:00	27.32	01/27/16	26.79
01/28/16 06:00	27.27	01/28/16	27.14
01/29/16 06:00	25.46	01/29/16	26.87
01/30/16 06:00	26.73	01/30/16	26.52
01/31/16 06:00	27.12	01/31/16	26.78
02/01/16 06:00	27.27	02/01/16	26.85
02/02/16 06:00	27.17	02/02/16	26.84
02/03/16 06:00	27.37	02/03/16	26.97
02/04/16 06:00	27.32	02/04/16	27.17
02/05/16 06:00	27.17	02/05/16	26.98
02/06/16 06:00	26.20	02/06/16	26.65
02/07/16 06:00	25.13	02/07/16	26.35
02/08/16 06:00	26.73	02/08/16	26.42
02/09/16 06:00	25.95	02/09/16	26.50
02/10/16 06:00	25.46	02/10/16	26.57
02/11/16 06:00	25.27	02/11/16	26.54
02/12/16 06:00	26.29	02/12/16	26.48
02/13/16 06:00	26.20	02/13/16	26.33
02/14/16 06:00	26.68	02/14/16	26.52
02/15/16 06:00	26.73	02/15/16	26.44
02/16/16 06:00	26.88	02/16/16	26.46
02/17/16 06:00	26.98	02/17/16	26.55
02/18/16 06:00	27.17	02/18/16	26.70
02/19/16 06:00	26.98	02/19/16	26.59
02/20/16 06:00	26.78	02/20/16	26.51
02/21/16 06:00	26.63	02/21/16	26.61
02/22/16 06:00	26.68	02/22/16	26.67
02/23/16 06:00	26.68	02/23/16	26.64
02/24/16 06:00	26.98	02/24/16	26.93
02/25/16 06:00	26.29	02/25/16	26.92
02/26/16 06:00	26.00	02/26/16	26.31
02/27/16 06:00	26.49	02/27/16	26.23
02/28/16 06:00	26.39	02/28/16	25.96

02/29/16 06:00	26.34	02/29/16	26.40
03/01/16 06:00	26.59	03/01/16	26.30
03/02/16 06:00	26.78	03/02/16	26.48
03/03/16 06:00	27.47	03/03/16	26.53
03/04/16 06:00	27.37	03/04/16	26.48
03/05/16 06:00	27.17	03/05/16	26.75
03/06/16 06:00	26.88	03/06/16	26.69
03/07/16 06:00	26.88	03/07/16	26.68
03/08/16 06:00	26.54	03/08/16	26.64
03/09/16 06:00	26.98	03/09/16	26.80
03/10/16 06:00	27.03	03/10/16	26.81
03/11/16 06:00	26.88	03/11/16	26.92
03/12/16 06:00	26.98	03/12/16	26.99
03/13/16 06:00	26.88	03/13/16	26.94
03/14/16 06:00	26.98	03/14/16	27.05
03/15/16 06:00	27.27	03/15/16	27.21
03/16/16 06:00	27.47	03/16/16	27.25
03/17/16 06:00	27.47	03/17/16	27.20
03/18/16 06:00	27.42	03/18/16	27.56
03/19/16 06:00	27.52	03/19/16	27.38
03/20/16 06:00	27.76	03/20/16	27.75
03/21/16 06:00	27.27	03/21/16	27.52
03/22/16 06:00	26.88	03/22/16	26.96
03/23/16 06:00	26.98	03/23/16	26.87
03/24/16 06:00	27.17	03/24/16	26.99
03/25/16 06:00	27.37	03/25/16	27.32
03/26/16 06:00	27.22	03/26/16	27.68
03/27/16 06:00	27.42	03/27/16	27.43
03/28/16 06:00	27.42	03/28/16	27.26
03/29/16 06:00	27.52	03/29/16	27.44
03/30/16 06:00	27.62	03/30/16	27.56
03/31/16 06:00	27.67	03/31/16	27.49
04/01/16 06:00	27.86	04/01/16	27.81
04/02/16 06:00	28.06	04/02/16	27.98
04/03/16 06:00	28.51	04/03/16	27.83
04/04/16 06:00	28.41	04/04/16	28.20
04/05/16 06:00	28.46	04/05/16	28.19
04/06/16 06:00	28.06	04/06/16	27.92
04/07/16 06:00	28.11	04/07/16	28.18
04/08/16 06:00	27.81	04/08/16	28.33
04/09/16 06:00	28.16	04/09/16	28.16
04/10/16 06:00	27.96	04/10/16	27.97
04/11/16 06:00	27.81	04/11/16	27.99
04/12/16 06:00	27.86	04/12/16	28.05
04/13/16 06:00	27.96	04/13/16	27.33

04/14/16 06:00	28.06	04/14/16	27.78
04/15/16 06:00	27.86	04/15/16	28.01
04/16/16 06:00	28.11	04/16/16	28.12
04/17/16 06:00	28.16	04/17/16	28.20
04/18/16 06:00	28.06	04/18/16	27.85
04/19/16 06:00	28.01	04/19/16	27.44
04/20/16 06:00	27.71	04/20/16	27.57
04/21/16 06:00	27.91	04/21/16	27.57
04/22/16 06:00	28.16	04/22/16	27.87
04/23/16 06:00	28.95	04/23/16	28.08
04/24/16 06:00	29.45	04/24/16	27.82
04/25/16 06:00	28.31	04/25/16	27.90
04/26/16 06:00	28.51	04/26/16	28.05
04/27/16 06:00	29.15	04/27/16	28.24
04/28/16 06:00	28.56	04/28/16	28.09
04/29/16 06:00	28.66	04/29/16	28.05
04/30/16 06:00	28.66	04/30/16	28.16
05/01/16 06:00	28.80	05/01/16	28.16
05/02/16 06:00	28.80	05/02/16	28.36
05/03/16 06:00	28.85	05/03/16	28.47
05/04/16 06:00	29.05	05/04/16	28.67
05/05/16 06:00	29.50	05/05/16	28.89
05/06/16 06:00	28.21	05/06/16	28.88
05/07/16 06:00	28.46	05/07/16	28.66
05/08/16 06:00	28.56	05/08/16	28.46
05/09/16 06:00	27.86	05/09/16	28.40
05/10/16 06:00	28.16	05/10/16	28.41
05/11/16 06:00	28.36	05/11/16	28.59
05/12/16 06:00	28.66	05/12/16	28.88
05/13/16 06:00	28.75	05/13/16	28.93
05/14/16 06:00	28.95	05/14/16	28.93
05/15/16 06:00	28.90	05/15/16	29.00
05/16/16 06:00	28.85	05/16/16	28.93
05/17/16 06:00	29.05	05/17/16	28.54
05/18/16 06:00	29.30	05/18/16	28.91
05/19/16 06:00	29.35	05/19/16	29.07
05/20/16 06:00	29.25	05/20/16	29.14
05/21/16 06:00	29.25	05/21/16	29.08
05/22/16 06:00	29.00	05/22/16	28.95
05/23/16 06:00	29.05	05/23/16	29.00
05/24/16 06:00	29.05	05/24/16	28.97
05/25/16 06:00	29.35	05/25/16	29.13
05/26/16 06:00	29.55	05/26/16	29.41
05/27/16 06:00	29.55	05/27/16	29.51
05/28/16 06:00	29.65	05/28/16	29.50

05/29/16 06:00	29.60	05/29/16	29.44
05/30/16 06:00	29.85	05/30/16	29.53
05/31/16 06:00	30.26	05/31/16	29.62
06/01/16 06:00	29.70	06/01/16	29.50
06/02/16 06:00	29.70	06/02/16	29.30
06/03/16 06:00	29.60	06/03/16	29.39
06/04/16 06:00	29.80	06/04/16	29.35
06/05/16 06:00	29.65	06/05/16	29.48
06/06/16 06:00	29.75	06/06/16	29.36
06/07/16 06:00	30.26	06/07/16	29.48
06/08/16 06:00	30.66	06/08/16	29.86
06/09/16 06:00	30.36	06/09/16	30.27
06/10/16 06:00	30.26	06/10/16	29.56
06/11/16 06:00		06/11/16	29.47
06/12/16 06:00	29.47	06/12/16	29.38
06/13/16 06:00	29.40	06/13/16	29.55
06/14/16 06:00	29.20	06/14/16	29.54
06/15/16 06:00	29.24	06/15/16	29.70
06/16/16 06:00	29.20	06/16/16	29.50
06/17/16 06:00	29.47	06/17/16	29.29
06/18/16 06:00	29.06	06/18/16	29.13
06/19/16 06:00	28.99	06/19/16	28.90
06/20/16 06:00	28.94	06/20/16	28.84
06/21/16 06:00	28.80	06/21/16	28.85
06/22/16 06:00	28.80	06/22/16	28.84
06/23/16 06:00	28.83	06/23/16	28.96
06/24/16 06:00	28.46	06/24/16	29.35
06/25/16 06:00	28.57	06/25/16	29.01
06/26/16 06:00	28.79	06/26/16	28.98
06/27/16 06:00	29.04	06/27/16	29.14
06/28/16 06:00	28.79	06/28/16	28.85
06/29/16 06:00	29.04	06/29/16	29.05
06/30/16 06:00	29.21	06/30/16	29.05
07/01/16 06:00	29.26	07/01/16	29.38
07/02/16 06:00	29.38	07/02/16	29.47
07/03/16 06:00	29.23	07/03/16	29.59
07/04/16 06:00	29.21	07/04/16	29.42
07/05/16 06:00	29.00	07/05/16	29.28
07/06/16 06:00	29.09	07/06/16	29.43
07/07/16 06:00	29.19	07/07/16	29.32
07/08/16 06:00	29.48	07/08/16	29.27
07/09/16 06:00	29.33	07/09/16	28.90
07/10/16 06:00	29.38	07/10/16	29.30
07/11/16 06:00	29.36	07/11/16	29.34
07/12/16 06:00	29.50	07/12/16	29.27

07/13/16 06:00	29.43	07/13/16	29.39
07/14/16 06:00	29.40	07/14/16	29.30
07/15/16 06:00	29.60	07/15/16	29.46
07/16/16 06:00	29.71	07/16/16	29.94
07/17/16 06:00	29.43	07/17/16	29.86
07/18/16 06:00	29.73	07/18/16	29.85
07/19/16 06:00	29.81	07/19/16	29.48
07/20/16 06:00	29.44	07/20/16	29.43
07/21/16 06:00	29.26	07/21/16	29.92
07/22/16 06:00	29.94	07/22/16	29.83
07/23/16 06:00	29.71	07/23/16	29.90
07/24/16 06:00	29.65	07/24/16	29.47
07/25/16 06:00	29.46	07/25/16	29.15
07/26/16 06:00	29.54	07/26/16	29.56
07/27/16 06:00	29.58	07/27/16	29.66
07/28/16 06:00	29.51	07/28/16	29.79
07/29/16 06:00	29.61	07/29/16	29.49
07/30/16 06:00	29.54	07/30/16	29.34
07/31/16 06:00	29.36	07/31/16	29.65
08/01/16 06:00	29.51	08/01/16	29.82
08/02/16 06:00	29.80	08/02/16	30.01
08/03/16 06:00	29.40	08/03/16	29.71
08/04/16 06:00	28.80	08/04/16	29.39
08/05/16 06:00	29.17	08/05/16	29.30
08/06/16 06:00	29.00	08/06/16	29.58
08/07/16 06:00	29.01	08/07/16	29.34
08/08/16 06:00	29.26	08/08/16	29.42
08/09/16 06:00	29.57	08/09/16	29.44
08/10/16 06:00	29.56	08/10/16	29.46
08/11/16 06:00	29.40	08/11/16	29.45
08/12/16 06:00	29.90	08/12/16	29.57
08/13/16 06:00	30.05	08/13/16	29.67
08/14/16 06:00	29.88	08/14/16	29.61
08/15/16 06:00	29.74	08/15/16	29.88
08/16/16 06:00	29.73	08/16/16	29.51
08/17/16 06:00	29.64	08/17/16	29.38
08/18/16 06:00	29.34	08/18/16	29.31
08/19/16 06:00	29.47	08/19/16	29.40
08/20/16 06:00	29.34	08/20/16	29.39
08/21/16 06:00	29.30	08/21/16	29.36
08/22/16 06:00	29.20	08/22/16	29.12
08/23/16 06:00	29.17	08/23/16	29.22
08/24/16 06:00	29.40	08/24/16	29.41
08/25/16 06:00	29.51	08/25/16	29.53
08/26/16 06:00	29.60	08/26/16	29.31

08/27/16 06:00	30.00	08/27/16	29.98
08/28/16 06:00	30.04	08/28/16	30.26
08/29/16 06:00	30.18	08/29/16	30.24
08/30/16 06:00	30.34	08/30/16	30.17
08/31/16 06:00	30.54	08/31/16	30.36
09/01/16 06:00	30.31	09/01/16	30.12
09/02/16 06:00	29.90	09/02/16	30.48
09/03/16 06:00	30.69	09/03/16	30.45
09/04/16 06:00	29.77	09/04/16	30.23
09/05/16 06:00	29.74	09/05/16	29.88
09/06/16 06:00	29.74	09/06/16	29.39
09/07/16 06:00	29.80	09/07/16	29.35
09/08/16 06:00	30.02	09/08/16	29.29
09/09/16 06:00	30.07	09/09/16	30.00
09/10/16 06:00	30.05	09/10/16	30.01
09/11/16 06:00	30.05	09/11/16	30.01
09/12/16 06:00	30.52	09/12/16	30.01
09/13/16 06:00	30.17	09/13/16	29.94
09/14/16 06:00	29.84	09/14/16	29.72
09/15/16 06:00	29.91	09/15/16	29.71
09/16/16 06:00	29.77	09/16/16	29.87
09/17/16 06:00	29.80	09/17/16	29.73
09/18/16 06:00	30.54	09/18/16	29.78
09/19/16 06:00	30.20	09/19/16	29.82
09/20/16 06:00	30.32	09/20/16	29.50
09/21/16 06:00	30.22	09/21/16	29.66
09/22/16 06:00	30.34	09/22/16	29.78
09/23/16 06:00	29.83	09/23/16	29.79
09/24/16 06:00	29.77	09/24/16	29.86
09/25/16 06:00	29.36	09/25/16	30.01
09/26/16 06:00	29.58	09/26/16	29.61
09/27/16 06:00	29.65	09/27/16	29.53
09/28/16 06:00	29.61	09/28/16	29.54
09/29/16 06:00	29.67	09/29/16	29.71
09/30/16 06:00	30.28	09/30/16	29.50
10/01/16 06:00	30.11	10/01/16	29.56
10/02/16 06:00	30.17	10/02/16	29.63
10/03/16 06:00	29.83	10/03/16	29.80
10/04/16 06:00	29.77	10/04/16	30.03
10/05/16 06:00	30.08	10/05/16	30.15
10/06/16 06:00	30.68	10/06/16	30.31
10/07/16 06:00	30.86	10/07/16	30.42
10/08/16 06:00	30.91	10/08/16	30.30
10/09/16 06:00	30.42	10/09/16	30.19
10/10/16 06:00	30.27	10/10/16	29.82

10/11/16 06:00	30.41	10/11/16	29.81
10/12/16 06:00	30.15	10/12/16	29.57
10/13/16 06:00	30.25	10/13/16	29.45
10/14/16 06:00	30.12	10/14/16	29.53
10/15/16 06:00	29.83	10/15/16	29.34
10/16/16 06:00	29.63	10/16/16	29.35
10/17/16 06:00	29.91	10/17/16	29.48
10/18/16 06:00	29.83	10/18/16	29.37
10/19/16 06:00	30.07	10/19/16	29.34
10/20/16 06:00	29.67	10/20/16	29.43
10/21/16 06:00	30.04	10/21/16	29.46
10/22/16 06:00	30.00	10/22/16	29.09
10/23/16 06:00	29.94	10/23/16	29.36
10/24/16 06:00	29.91	10/24/16	29.12
10/25/16 06:00	29.67	10/25/16	29.04
10/26/16 06:00	29.70	10/26/16	29.10
10/27/16 06:00	29.60	10/27/16	29.03
10/28/16 06:00	29.43	10/28/16	28.99
10/29/16 06:00	29.43	10/29/16	28.82
10/30/16 06:00	29.19	10/30/16	28.83
10/31/16 06:00	29.47	10/31/16	28.81
11/01/16 06:00	29.31	11/01/16	28.78
11/02/16 06:00	28.94	11/02/16	28.47
11/03/16 06:00	29.00	11/03/16	28.61
11/04/16 06:00	29.09	11/04/16	28.48
11/05/16 06:00	28.90	11/05/16	28.42
11/06/16 06:00	28.76	11/06/16	28.32
11/07/16 06:00	28.56	11/07/16	28.31
11/08/16 06:00	29.07	11/08/16	28.32
11/09/16 06:00	29.01	11/09/16	28.35
11/10/16 06:00	29.27	11/10/16	28.32
11/11/16 06:00	29.13	11/11/16	28.47
11/12/16 06:00	29.24	11/12/16	28.59
11/13/16 06:00	29.21	11/13/16	28.51
11/14/16 06:00	29.11	11/14/16	28.72
11/15/16 06:00	29.11	11/15/16	28.62
11/16/16 06:00	29.17	11/16/16	28.62
11/17/16 06:00	28.45	11/17/16	28.34
11/18/16 06:00	28.37	11/18/16	28.32
11/19/16 06:00	28.55	11/19/16	28.27
11/20/16 06:00	28.52	11/20/16	28.24
11/21/16 06:00	28.70	11/21/16	28.23
11/22/16 06:00	28.62	11/22/16	28.21
11/23/16 06:00	28.66	11/23/16	28.03
11/24/16 06:00	28.55	11/24/16	28.03

	11/25/16 06:00	28.60	11/25/16	28.01
	11/26/16 06:00	28.50	11/26/16	27.93
	11/27/16 06:00	28.10	11/27/16	27.74
	11/28/16 06:00	28.39	11/28/16	27.86
	11/29/16 06:00	27.96	11/29/16	27.88
	11/30/16 06:00	28.05	11/30/16	27.76
	12/01/16 06:00	28.67	12/01/16	27.62
	12/02/16 06:00	28.28	12/02/16	27.68
	12/03/16 06:00	28.19	12/03/16	27.91
	12/04/16 06:00	28.77	12/04/16	28.06
	12/05/16 06:00	28.60	12/05/16	27.48
	12/06/16 06:00	28.67	12/06/16	28.17
	12/07/16 06:00	28.79	12/07/16	28.35
	12/08/16 06:00	28.60	12/08/16	28.34
	12/09/16 06:00	28.83	12/09/16	28.17
	12/10/16 06:00	28.74	12/10/16	28.12
	12/11/16 06:00	28.74	12/11/16	28.09
	12/12/16 06:00	28.52	12/12/16	28.10
	12/13/16 06:00	28.37	12/13/16	27.95
	12/14/16 06:00	28.15	12/14/16	27.95
	12/15/16 06:00	27.95	12/15/16	27.85
	12/16/16 06:00	28.06	12/16/16	27.83
	12/17/16 06:00	28.15	12/17/16	27.86
	12/18/16 06:00	28.28	12/18/16	27.79
	12/19/16 06:00	28.28	12/19/16	27.82
	12/20/16 06:00	28.43	12/20/16	27.90
	12/21/16 06:00	28.39	12/21/16	27.87
	12/22/16 06:00	28.25	12/22/16	27.82
	12/23/16 06:00	28.37	12/23/16	27.67
	12/24/16 06:00	28.35	12/24/16	27.65
	12/25/16 06:00	28.23	12/25/16	27.58
	12/26/16 06:00	28.29	12/26/16	27.44
	12/27/16 06:00	28.09	12/27/16	27.44
	12/28/16 06:00	28.06	12/28/16	27.49
	12/29/16 06:00	28.02	12/29/16	27.47
	12/30/16 06:00	27.88	12/30/16	27.48
	12/31/16 06:00	27.95	12/31/16	27.60
2017	01/01/17 06:00	27.27	01/01/17	27.48
	01/02/17 06:00	27.37	01/02/17	27.44
	01/03/17 06:00	27.42	01/03/17	27.45
	01/04/17 06:00	27.47	01/04/17	27.38
	01/05/17 06:00	27.86	01/05/17	27.60
	01/06/17 06:00	27.96	01/06/17	27.74
	01/07/17 06:00	27.67	01/07/17	27.52
	01/08/17 06:00	27.12	01/08/17	27.22

01/09/17 06:00	27.27	01/09/17	27.27
01/10/17 06:00	27.37	01/10/17	27.17
01/11/17 06:00	27.17	01/11/17	27.01
01/12/17 06:00	26.78	01/12/17	27.01
01/13/17 06:00	26.68	01/13/17	26.90
01/14/17 06:00	26.88	01/14/17	26.97
01/15/17 06:00	26.88	01/15/17	27.00
01/16/17 06:00	27.17	01/16/17	26.86
01/17/17 06:00	26.88	01/17/17	26.82
01/18/17 06:00	26.93	01/18/17	26.75
01/19/17 06:00	26.93	01/19/17	26.75
01/20/17 06:00	26.78	01/20/17	26.98
01/21/17 06:00	27.08	01/21/17	27.09
01/22/17 06:00	27.47	01/22/17	27.28
01/23/17 06:00	27.22	01/23/17	27.31
01/24/17 06:00	25.81	01/24/17	26.73
01/25/17 06:00	26.54	01/25/17	26.75
01/26/17 06:00	26.73	01/26/17	26.81
01/27/17 06:00	27.32	01/27/17	26.74
01/28/17 06:00	27.37	01/28/17	27.04
01/29/17 06:00	27.47	01/29/17	27.00
01/30/17 06:00	25.66	01/30/17	26.77
01/31/17 06:00	26.34	01/31/17	26.64
02/01/17 06:00	26.59	02/01/17	26.83
02/02/17 06:00	26.73	02/02/17	26.70
02/03/17 06:00	26.83	02/03/17	26.72
02/04/17 06:00	26.88	02/04/17	26.75
02/05/17 06:00	26.93	02/05/17	26.64
02/06/17 06:00	26.98	02/06/17	26.71
02/07/17 06:00	26.73	02/07/17	26.63
02/08/17 06:00	26.73	02/08/17	26.74
02/09/17 06:00	27.27	02/09/17	26.91
02/10/17 06:00	27.37	02/10/17	26.97
02/11/17 06:00	26.78	02/11/17	26.84
02/12/17 06:00	26.78	02/12/17	26.76
02/13/17 06:00	26.78	02/13/17	26.74
02/14/17 06:00	26.88	02/14/17	26.79
02/15/17 06:00	27.03	02/15/17	26.99
02/16/17 06:00	27.47	02/16/17	27.18
02/17/17 06:00	27.27	02/17/17	27.15
02/18/17 06:00	26.98	02/18/17	26.98
02/19/17 06:00	27.08	02/19/17	27.11
02/20/17 06:00	27.17	02/20/17	27.01
02/21/17 06:00	26.88	02/21/17	26.97
02/22/17 06:00	27.57	02/22/17	26.86

02/23/17 06:00	26.29	02/23/17	26.85
02/24/17 06:00	26.54	02/24/17	26.68
02/25/17 06:00	26.88	02/25/17	26.83
02/26/17 06:00	27.37	02/26/17	27.13
02/27/17 06:00	27.62	02/27/17	27.11
02/28/17 06:00	27.08	02/28/17	27.23
03/01/17 06:00	26.98	03/01/17	27.16
03/02/17 06:00	27.08	03/02/17	27.12
03/03/17 06:00	27.22	03/03/17	27.26
03/04/17 06:00	27.27	03/04/17	27.19
03/05/17 06:00	27.22	03/05/17	27.02
03/06/17 06:00	27.08	03/06/17	26.93
03/07/17 06:00	27.12	03/07/17	27.03
03/08/17 06:00	27.17	03/08/17	26.94
03/09/17 06:00	26.78	03/09/17	26.92
03/10/17 06:00	26.78	03/10/17	26.90
03/11/17 06:00	26.78	03/11/17	26.87
03/12/17 06:00	26.73	03/12/17	27.07
03/13/17 06:00	27.27	03/13/17	26.87
03/14/17 06:00	27.76	03/14/17	26.95
03/15/17 06:00	27.08	03/15/17	26.87
03/16/17 06:00	26.49	03/16/17	26.66
03/17/17 06:00	26.78	03/17/17	26.88
03/18/17 06:00	26.78	03/18/17	26.83
03/19/17 06:00	26.78	03/19/17	26.71
03/20/17 06:00	26.73	03/20/17	26.62
03/21/17 06:00	26.88	03/21/17	26.60
03/22/17 06:00	26.98	03/22/17	26.77
03/23/17 06:00	27.27	03/23/17	26.83
03/24/17 06:00	26.73	03/24/17	26.84
03/25/17 06:00	26.59	03/25/17	26.76
03/26/17 06:00	27.17	03/26/17	26.84
03/27/17 06:00	27.08	03/27/17	26.84
03/28/17 06:00	27.03	03/28/17	26.81
03/29/17 06:00	26.78	03/29/17	26.93
03/30/17 06:00	26.98	03/30/17	26.91
03/31/17 06:00	27.27	03/31/17	27.21
04/01/17 06:00	27.96	04/01/17	27.56
04/02/17 06:00	27.96	04/02/17	27.23
04/03/17 06:00	27.96	04/03/17	27.27
04/04/17 06:00	27.86	04/04/17	27.01
04/05/17 06:00	27.96	04/05/17	27.06
04/06/17 06:00	27.76	04/06/17	27.20
04/07/17 06:00	28.06	04/07/17	27.50
04/08/17 06:00	27.96	04/08/17	28.01

04/09/17 06:00	28.11	04/09/17	27.76
04/10/17 06:00	27.81	04/10/17	27.82
04/11/17 06:00	27.91	04/11/17	27.91
04/12/17 06:00	27.37	04/12/17	27.78
04/13/17 06:00	27.27	04/13/17	27.81
04/14/17 06:00	27.47	04/14/17	27.67
04/15/17 06:00	27.52	04/15/17	27.74
04/16/17 06:00	27.47	04/16/17	27.61
04/17/17 06:00	27.57	04/17/17	27.66
04/18/17 06:00	27.62	04/18/17	27.51
04/19/17 06:00	27.47	04/19/17	27.47
04/20/17 06:00	27.47	04/20/17	27.90
04/21/17 06:00	27.81	04/21/17	27.81
04/22/17 06:00	28.31	04/22/17	27.84
04/23/17 06:00	28.95	04/23/17	27.98
04/24/17 06:00	28.80	04/24/17	28.30
04/25/17 06:00	28.16	04/25/17	28.33
04/26/17 06:00	27.96	04/26/17	28.24
04/27/17 06:00	28.36	04/27/17	28.12
04/28/17 06:00	28.66	04/28/17	28.21
04/29/17 06:00	28.56	04/29/17	28.10
04/30/17 06:00	28.16	04/30/17	28.06
05/01/17 06:00	28.16	05/01/17	28.07
05/02/17 06:00	28.41	05/02/17	28.02
05/03/17 06:00	28.56	05/03/17	28.34
05/04/17 06:00	28.56	05/04/17	28.10
05/05/17 06:00	28.51	05/05/17	28.23
05/06/17 06:00	28.06	05/06/17	28.08
05/07/17 06:00	27.91	05/07/17	27.72
05/08/17 06:00	27.86	05/08/17	27.87
05/09/17 06:00	28.06	05/09/17	28.02
05/10/17 06:00	28.26	05/10/17	28.13
05/11/17 06:00	28.21	05/11/17	28.14
05/12/17 06:00	28.26	05/12/17	28.38
05/13/17 06:00	28.46	05/13/17	28.60
05/14/17 06:00	29.05	05/14/17	28.62
05/15/17 06:00	29.05	05/15/17	28.94
05/16/17 06:00	29.35	05/16/17	29.04
05/17/17 06:00	29.35	05/17/17	29.06
05/18/17 06:00	30.00	05/18/17	29.05
05/19/17 06:00	29.25	05/19/17	28.83
05/20/17 06:00	29.05	05/20/17	28.92
05/21/17 06:00	28.95	05/21/17	28.69
05/22/17 06:00	28.85	05/22/17	28.84
05/23/17 06:00	28.66	05/23/17	28.85

05/24/17 06:00	28.95	05/24/17	28.76
05/25/17 06:00	29.05	05/25/17	28.90
05/26/17 06:00	29.35	05/26/17	29.02
05/27/17 06:00	29.65	05/27/17	29.09
05/28/17 06:00	29.25	05/28/17	28.96
05/29/17 06:00	29.15	05/29/17	29.29
05/30/17 06:00	29.05	05/30/17	29.09
05/31/17 06:00	29.10	05/31/17	29.15
06/01/17 06:00	29.10	06/01/17	29.15
06/02/17 06:00	29.05	06/02/17	29.01
06/03/17 06:00	29.05	06/03/17	28.97
06/04/17 06:00	28.85	06/04/17	29.09
06/05/17 06:00	28.85	06/05/17	29.21
06/06/17 06:00	29.15	06/06/17	29.37
06/07/17 06:00	29.35	06/07/17	29.73
06/08/17 06:00	30.15	06/08/17	29.62
06/09/17 06:00	30.46	06/09/17	29.69
06/10/17 06:00	29.95	06/10/17	29.57
06/11/17 06:00	29.55	06/11/17	29.58
06/12/17 06:00	29.25	06/12/17	29.34
06/13/17 06:00	29.10	06/13/17	29.35
06/14/17 06:00	29.05	06/14/17	29.17
06/15/17 06:00	28.80	06/15/17	29.00
06/16/17 06:00	29.05	06/16/17	28.95
06/17/17 06:00	28.90	06/17/17	28.87
06/18/17 06:00	29.05	06/18/17	28.81
06/19/17 06:00	29.25	06/19/17	28.65
06/20/17 06:00	29.15	06/20/17	28.60
06/21/17 06:00	28.85	06/21/17	28.68
06/22/17 06:00	28.90	06/22/17	28.92
06/23/17 06:00	28.75	06/23/17	28.87
06/24/17 06:00	28.95	06/24/17	28.96
06/25/17 06:00	28.85	06/25/17	28.82
06/26/17 06:00	28.95	06/26/17	28.74
06/27/17 06:00	29.25	06/27/17	29.16
06/28/17 06:00	29.25	06/28/17	29.11
06/29/17 06:00	29.20	06/29/17	28.90
06/30/17 06:00	29.15	06/30/17	28.91
07/01/17 06:00	29.15	07/01/17	29.28
07/02/17 06:00	29.15	07/02/17	29.01
07/03/17 06:00	29.20	07/03/17	28.94
07/04/17 06:00	29.40	07/04/17	29.05
07/05/17 06:00	29.20	07/05/17	29.25
07/06/17 06:00	28.75	07/06/17	29.21
07/07/17 06:00	28.75	07/07/17	29.04

07/08/17 06:00	28.90	07/08/17	28.81
07/09/17 06:00	29.00	07/09/17	28.74
07/10/17 06:00	29.25	07/10/17	29.02
07/11/17 06:00	29.25	07/11/17	29.07
07/12/17 06:00	29.20	07/12/17	28.95
07/13/17 06:00	29.30	07/13/17	28.77
07/14/17 06:00	29.45	07/14/17	28.89
07/15/17 06:00	29.65	07/15/17	29.05
07/16/17 06:00	29.75	07/16/17	29.58
07/17/17 06:00	29.65	07/17/17	29.71
07/18/17 06:00	29.45	07/18/17	29.16
07/19/17 06:00	29.65	07/19/17	29.33
07/20/17 06:00	29.30	07/20/17	29.38
07/21/17 06:00	29.35	07/21/17	29.10
07/22/17 06:00	29.30	07/22/17	29.06
07/23/17 06:00	29.35	07/23/17	29.06
07/24/17 06:00	29.35	07/24/17	29.09
07/25/17 06:00	29.65	07/25/17	29.21
07/26/17 06:00	29.40	07/26/17	29.28
07/27/17 06:00	29.70	07/27/17	29.32
07/28/17 06:00	29.95	07/28/17	29.23
07/29/17 06:00	30.15	07/29/17	29.43
07/30/17 06:00	30.15	07/30/17	30.07
07/31/17 06:00	30.66	07/31/17	29.89
08/01/17 06:00	29.85	08/01/17	30.04
08/02/17 06:00	29.50	08/02/17	29.72
08/03/17 06:00	29.60	08/03/17	29.48
08/04/17 06:00	30.26	08/04/17	29.83
08/05/17 06:00	29.80	08/05/17	29.86
08/06/17 06:00	29.95	08/06/17	29.81
08/07/17 06:00	30.08	08/07/17	30.13
08/08/17 06:00	29.45	08/08/17	29.82
08/09/17 06:00	29.50	08/09/17	29.71
08/10/17 06:00	29.45	08/10/17	29.71
08/11/17 06:00	29.45	08/11/17	29.69
08/12/17 06:00	29.45	08/12/17	29.62
08/13/17 06:00	29.75	08/13/17	29.92
08/14/17 06:00	30.05	08/14/17	29.71
08/15/17 06:00	29.83	08/15/17	29.80
08/16/17 06:00	29.85	08/16/17	29.80
08/17/17 06:00	30.03	08/17/17	29.79
08/18/17 06:00	30.15	08/18/17	29.64
08/19/17 06:00	30.15	08/19/17	29.59
08/20/17 06:00	30.31	08/20/17	30.17
08/21/17 06:00	29.95	08/21/17	29.68

08/22/17 06:00	29.65	08/22/17	29.75
08/23/17 06:00	30.23	08/23/17	29.49
08/24/17 06:00	30.26	08/24/17	29.71
08/25/17 06:00	29.83	08/25/17	29.62
08/26/17 06:00	29.88	08/26/17	29.75
08/27/17 06:00	29.85	08/27/17	29.66
08/28/17 06:00	29.75	08/28/17	29.96
08/29/17 06:00	29.93	08/29/17	30.21
08/30/17 06:00	29.85	08/30/17	30.15
08/31/17 06:00	29.78	08/31/17	29.80
09/01/17 06:00	29.83	09/01/17	29.58
09/02/17 06:00	29.78	09/02/17	29.58
09/03/17 06:00	29.40	09/03/17	29.67
09/04/17 06:00	29.58	09/04/17	29.52
09/05/17 06:00	30.00	09/05/17	29.79
09/06/17 06:00	30.20	09/06/17	29.61
09/07/17 06:00	30.71	09/07/17	30.64
09/08/17 06:00	30.96	09/08/17	30.41
09/09/17 06:00	31.32	09/09/17	30.45
09/10/17 06:00	31.47	09/10/17	30.13
09/11/17 06:00	31.06	09/11/17	30.46
09/12/17 06:00	31.01	09/12/17	30.21
09/13/17 06:00	31.04	09/13/17	30.36
09/14/17 06:00	30.18	09/14/17	30.69
09/15/17 06:00	30.05	09/15/17	30.17
09/16/17 06:00	30.18	09/16/17	29.96
09/17/17 06:00	30.56	09/17/17	30.15
09/18/17 06:00	30.73	09/18/17	30.19
09/19/17 06:00	30.28	09/19/17	30.24
09/20/17 06:00	30.38	09/20/17	30.30
09/21/17 06:00	29.88	09/21/17	30.16
09/22/17 06:00	30.48	09/22/17	30.26
09/23/17 06:00	30.66	09/23/17	30.26
09/24/17 06:00	30.94	09/24/17	30.18
09/25/17 06:00	30.46	09/25/17	30.25
09/26/17 06:00	30.56	09/26/17	30.00
09/27/17 06:00	30.36	09/27/17	30.24
09/28/17 06:00	30.33	09/28/17	30.13
09/29/17 06:00	30.36	09/29/17	30.10
09/30/17 06:00	30.66	09/30/17	29.89
10/01/17 06:00	30.05	10/01/17	29.88
10/02/17 06:00	30.13	10/02/17	29.75
10/03/17 06:00	29.95	10/03/17	29.76
10/04/17 06:00	29.65	10/04/17	29.92
10/05/17 06:00	30.05	10/05/17	29.73

10/06/17 06:00	29.48	10/06/17	29.59
10/07/17 06:00	29.20	10/07/17	29.42
10/08/17 06:00	29.75	10/08/17	29.52
10/09/17 06:00	30.36	10/09/17	29.78
10/10/17 06:00	29.65	10/10/17	30.05
10/11/17 06:00	30.03	10/11/17	30.04
10/12/17 06:00	29.78	10/12/17	29.86
10/13/17 06:00	29.28	10/13/17	29.54
10/14/17 06:00	29.58	10/14/17	29.22
10/15/17 06:00	29.55	10/15/17	29.87
10/16/17 06:00	29.58	10/16/17	29.31
10/17/17 06:00	29.85	10/17/17	29.49
10/18/17 06:00	29.95	10/18/17	29.32
10/19/17 06:00	29.50	10/19/17	29.27
10/20/17 06:00	28.58	10/20/17	29.30
10/21/17 06:00	28.93	10/21/17	29.37
10/22/17 06:00	28.66	10/22/17	29.24
10/23/17 06:00	28.95	10/23/17	29.32
10/24/17 06:00	29.05	10/24/17	29.39
10/25/17 06:00	28.61	10/25/17	29.31
10/26/17 06:00	28.06	10/26/17	29.14
10/27/17 06:00	28.06	10/27/17	28.87
10/28/17 06:00	28.63	10/28/17	28.86
10/29/17 06:00	28.46	10/29/17	28.82
10/30/17 06:00	28.80	10/30/17	28.76
10/31/17 06:00	28.75	10/31/17	28.74
11/01/17 06:00	28.66	11/01/17	28.54
11/02/17 06:00	28.75	11/02/17	28.52
11/03/17 06:00	28.80	11/03/17	28.35
11/04/17 06:00	28.31	11/04/17	28.42
11/05/17 06:00	28.13	11/05/17	28.41
11/06/17 06:00	28.38	11/06/17	28.44
11/07/17 06:00	28.56	11/07/17	28.64
11/08/17 06:00	28.16	11/08/17	28.55
11/09/17 06:00	28.46	11/09/17	28.44
11/10/17 06:00	28.26	11/10/17	28.52
11/11/17 06:00	28.56	11/11/17	28.29
11/12/17 06:00	28.66	11/12/17	28.30
11/13/17 06:00	28.31	11/13/17	28.04
11/14/17 06:00	28.46	11/14/17	28.17
11/15/17 06:00	28.46	11/15/17	28.09
11/16/17 06:00	28.38	11/16/17	28.14
11/17/17 06:00	28.18	11/17/17	28.17
11/18/17 06:00	28.18	11/18/17	28.11
11/19/17 06:00	28.46	11/19/17	28.16

	11/20/17 06:00	27.42	11/20/17	28.00
	11/21/17 06:00	28.26	11/21/17	28.15
	11/22/17 06:00	28.26	11/22/17	28.06
	11/23/17 06:00	27.67	11/23/17	28.08
	11/24/17 06:00	27.89	11/24/17	27.81
	11/25/17 06:00	27.86	11/25/17	27.72
	11/26/17 06:00	27.71	11/26/17	27.81
	11/27/17 06:00	27.91	11/27/17	27.82
	11/28/17 06:00	27.79	11/28/17	27.84
	11/29/17 06:00	27.94	11/29/17	27.48
	11/30/17 06:00	27.96	11/30/17	27.36
	12/01/17 06:00	27.96	12/01/17	27.47
	12/02/17 06:00	27.91	12/02/17	27.53
	12/03/17 06:00	27.71	12/03/17	27.59
	12/04/17 06:00	27.52	12/04/17	27.55
	12/05/17 06:00	27.52	12/05/17	27.62
	12/06/17 06:00	27.57	12/06/17	27.60
	12/07/17 06:00	28.08	12/07/17	27.49
	12/08/17 06:00	27.62	12/08/17	27.54
	12/09/17 06:00	27.35	12/09/17	27.40
	12/10/17 06:00	27.07	12/10/17	27.24
	12/11/17 06:00	27.22	12/11/17	26.96
	12/12/17 06:00	27.27	12/12/17	27.22
	12/13/17 06:00	27.15	12/13/17	27.19
	12/14/17 06:00	27.17	12/14/17	27.08
	12/15/17 06:00	27.12	12/15/17	26.79
	12/16/17 06:00	26.85	12/16/17	27.03
	12/17/17 06:00	27.27	12/17/17	26.99
	12/18/17 06:00	27.25	12/18/17	26.96
	12/19/17 06:00	27.22	12/19/17	26.97
	12/20/17 06:00	27.20	12/20/17	27.15
	12/21/17 06:00	27.08	12/21/17	27.17
	12/22/17 06:00	27.17	12/22/17	27.19
	12/23/17 06:00	27.00	12/23/17	27.07
	12/24/17 06:00	27.08	12/24/17	27.15
	12/25/17 06:00	27.03	12/25/17	27.10
	12/26/17 06:00	27.17	12/26/17	26.91
	12/27/17 06:00	26.98	12/27/17	26.88
	12/28/17 06:00	27.10	12/28/17	27.01
	12/29/17 06:00	27.17	12/29/17	27.12
	12/30/17 06:00	27.12	12/30/17	27.04
	12/31/17 06:00	27.17	12/31/17	26.83
2018	01/01/18 06:00	27.27	01/01/18	26.80
	01/02/18 06:00	26.29	01/02/18	26.87
	01/03/18 06:00	25.95	01/03/18	26.78

01/04/18 06:00	26.10	01/04/18	26.86
01/05/18 06:00	25.66	01/05/18	26.84
01/06/18 06:00	26.39	01/06/18	26.79
01/07/18 06:00	26.59	01/07/18	26.50
01/08/18 06:00	26.78	01/08/18	26.51
01/09/18 06:00	26.68	01/09/18	26.52
01/10/18 06:00	26.59	01/10/18	26.36
01/11/18 06:00	26.78	01/11/18	26.19
01/12/18 06:00	26.98	01/12/18	26.19
01/13/18 06:00	25.88	01/13/18	25.98
01/14/18 06:00	25.51	01/14/18	26.06
01/15/18 06:00	25.85	01/15/18	26.06
01/16/18 06:00	25.90	01/16/18	26.11
01/17/18 06:00	25.59	01/17/18	26.02
01/18/18 06:00	26.00	01/18/18	26.08
01/19/18 06:00	25.95	01/19/18	25.94
01/20/18 06:00	26.00	01/20/18	25.89
01/21/18 06:00	26.10	01/21/18	25.77
01/22/18 06:00	26.39	01/22/18	26.10
01/23/18 06:00	26.39	01/23/18	26.29
01/24/18 06:00	26.63	01/24/18	26.40
01/25/18 06:00	26.59	01/25/18	26.29
01/26/18 06:00	26.49	01/26/18	26.30
01/27/18 06:00	26.49	01/27/18	26.23
01/28/18 06:00	26.39	01/28/18	26.05
01/29/18 06:00	26.46	01/29/18	26.09
01/30/18 06:00	25.93	01/30/18	26.27
01/31/18 06:00	25.90	01/31/18	25.96
02/01/18 06:00	26.00	02/01/18	25.88
02/02/18 06:00	26.02	02/02/18	26.00
02/03/18 06:00	25.98	02/03/18	25.94
02/04/18 06:00	26.29	02/04/18	26.08
02/05/18 06:00	26.32	02/05/18	26.29
02/06/18 06:00	26.51	02/06/18	26.42
02/07/18 06:00	26.68	02/07/18	26.39
02/08/18 06:00	26.68	02/08/18	26.60
02/09/18 06:00	26.49	02/09/18	26.48
02/10/18 06:00	26.59	02/10/18	26.58
02/11/18 06:00	26.49	02/11/18	26.47
02/12/18 06:00	26.68	02/12/18	26.59
02/13/18 06:00	26.63	02/13/18	26.68
02/14/18 06:00	26.61	02/14/18	26.53
02/15/18 06:00	26.46	02/15/18	26.43
02/16/18 06:00	26.37	02/16/18	26.36
02/17/18 06:00	26.22	02/17/18	26.41

02/18/18 06:00	26.29	02/18/18	26.43
02/19/18 06:00	26.32	02/19/18	26.39
02/20/18 06:00	26.49	02/20/18	26.57
02/21/18 06:00	26.39	02/21/18	26.41
02/22/18 06:00	26.39	02/22/18	26.47
02/23/18 06:00	26.46	02/23/18	26.48
02/24/18 06:00	26.59	02/24/18	26.44
02/25/18 06:00	26.59	02/25/18	26.67
02/26/18 06:00	26.59	02/26/18	26.69
02/27/18 06:00	26.54	02/27/18	26.72
02/28/18 06:00	26.63	02/28/18	26.66
03/01/18 06:00	26.29	03/01/18	26.65
03/02/18 06:00	26.73	03/02/18	26.65
03/03/18 06:00	26.78	03/03/18	26.84
03/04/18 06:00	26.76	03/04/18	26.82
03/05/18 06:00	26.68	03/05/18	26.75
03/06/18 06:00	26.32	03/06/18	26.67
03/07/18 06:00	26.61	03/07/18	26.67
03/08/18 06:00	26.88	03/08/18	26.66
03/09/18 06:00	26.98	03/09/18	26.85
03/10/18 06:00	26.85	03/10/18	26.89
03/11/18 06:00	26.88	03/11/18	26.73
03/12/18 06:00	26.98	03/12/18	27.02
03/13/18 06:00	27.69	03/13/18	27.30
03/14/18 06:00	27.00	03/14/18	26.98
03/15/18 06:00	26.78	03/15/18	26.72
03/16/18 06:00	26.51	03/16/18	26.64
03/17/18 06:00	26.88	03/17/18	26.69
03/18/18 06:00	26.85	03/18/18	26.84
03/19/18 06:00	27.00	03/19/18	27.22
03/20/18 06:00	27.05	03/20/18	27.01
03/21/18 06:00	27.08	03/21/18	27.18
03/22/18 06:00	27.27	03/22/18	27.26
03/23/18 06:00	27.15	03/23/18	27.07
03/24/18 06:00	27.12	03/24/18	26.96
03/25/18 06:00	27.08	03/25/18	26.85
03/26/18 06:00	27.20	03/26/18	26.83
03/27/18 06:00	27.27	03/27/18	26.91
03/28/18 06:00	27.17	03/28/18	26.94
03/29/18 06:00	27.17	03/29/18	27.04
03/30/18 06:00	27.27	03/30/18	26.95
03/31/18 06:00	26.98	03/31/18	27.27
04/01/18 06:00	27.71	04/01/18	27.27
04/02/18 06:00	27.35	04/02/18	27.29
04/03/18 06:00	27.67	04/03/18	27.54

04/04/18 06:00	27.67	04/04/18	27.51
04/05/18 06:00	27.57	04/05/18	27.53
04/06/18 06:00	27.91	04/06/18	27.62
04/07/18 06:00	28.26	04/07/18	27.29
04/08/18 06:00	28.11	04/08/18	27.33
04/09/18 06:00	28.36	04/09/18	27.40
04/10/18 06:00	28.16	04/10/18	27.22
04/11/18 06:00	28.06	04/11/18	27.37
04/12/18 06:00	27.74	04/12/18	27.42
04/13/18 06:00	27.69	04/13/18	27.49
04/14/18 06:00	27.91	04/14/18	27.74
04/15/18 06:00	27.76	04/15/18	27.75
04/16/18 06:00	26.12	04/16/18	27.34
04/17/18 06:00	26.59	04/17/18	27.19
04/18/18 06:00	27.22	04/18/18	27.36
04/19/18 06:00	27.42	04/19/18	27.51
04/20/18 06:00	27.52	04/20/18	27.35
04/21/18 06:00	27.67	04/21/18	27.36
04/22/18 06:00	27.67	04/22/18	27.46
04/23/18 06:00	27.76	04/23/18	27.53
04/24/18 06:00	28.21	04/24/18	27.75
04/25/18 06:00	28.46	04/25/18	28.13
04/26/18 06:00	28.46	04/26/18	28.16
04/27/18 06:00	28.95	04/27/18	28.10
04/28/18 06:00	28.80	04/28/18	28.11
04/29/18 06:00	28.46	04/29/18	28.42
04/30/18 06:00	28.63	04/30/18	28.39
05/01/18 06:00	28.63	05/01/18	28.43
05/02/18 06:00	28.56	05/02/18	28.46
05/03/18 06:00	28.56	05/03/18	28.52
05/04/18 06:00	28.56	05/04/18	28.62
05/05/18 06:00	28.66	05/05/18	28.69
05/06/18 06:00	28.58	05/06/18	28.72
05/07/18 06:00	28.95	05/07/18	28.77
05/08/18 06:00	28.85	05/08/18	28.55
05/09/18 06:00	28.66	05/09/18	28.62
05/10/18 06:00	28.78	05/10/18	28.59
05/11/18 06:00	29.15	05/11/18	29.02
05/12/18 06:00	29.33	05/12/18	29.06
05/13/18 06:00	28.90	05/13/18	28.87
05/14/18 06:00	29.00	05/14/18	28.81
05/15/18 06:00	29.20	05/15/18	28.97
05/16/18 06:00	29.43	05/16/18	29.16
05/17/18 06:00	29.33	05/17/18	29.15
05/18/18 06:00	28.98	05/18/18	29.04

05/19/18 06:00	28.95	05/19/18	29.03
05/20/18 06:00	28.95	05/20/18	29.07
05/21/18 06:00	29.25	05/21/18	29.10
05/22/18 06:00	29.45	05/22/18	29.48
05/23/18 06:00	29.55	05/23/18	29.16
05/24/18 06:00	29.35	05/24/18	29.13
05/25/18 06:00	28.85	05/25/18	29.01
05/26/18 06:00	29.25	05/26/18	29.04
05/27/18 06:00	29.63	05/27/18	29.78
05/28/18 06:00	29.38	05/28/18	29.44
05/29/18 06:00	29.95	05/29/18	29.42
05/30/18 06:00	29.45	05/30/18	29.28
05/31/18 06:00	29.65	05/31/18	29.41
06/01/18 06:00	29.45	06/01/18	29.52
06/02/18 06:00	28.85	06/02/18	29.19
06/03/18 06:00	28.68	06/03/18	28.99
06/04/18 06:00	28.66	06/04/18	28.87
06/05/18 06:00	28.58	06/05/18	28.79
06/06/18 06:00	28.61	06/06/18	28.85
06/07/18 06:00	29.05	06/07/18	28.90
06/08/18 06:00	29.05	06/08/18	28.93
06/09/18 06:00	29.05	06/09/18	28.87
06/10/18 06:00	29.05	06/10/18	28.81
06/11/18 06:00	28.95	06/11/18	28.91
06/12/18 06:00	28.93	06/12/18	28.78
06/13/18 06:00	28.99	06/13/18	28.61
06/14/18 06:00	28.85	06/14/18	28.75
06/15/18 06:00	28.63	06/15/18	28.75
06/16/18 06:00	28.58	06/16/18	28.57
06/17/18 06:00	28.56	06/17/18	28.44
06/18/18 06:00	28.48	06/18/18	28.55
06/19/18 06:00	28.56	06/19/18	28.40
06/20/18 06:00	28.66	06/20/18	28.58
06/21/18 06:00	28.75	06/21/18	28.71
06/22/18 06:00	28.78	06/22/18	28.57
06/23/18 06:00	28.70	06/23/18	28.63
06/24/18 06:00	28.73	06/24/18	28.86
06/25/18 06:00	28.83	06/25/18	28.98
06/26/18 06:00	28.85	06/26/18	28.99
06/27/18 06:00	28.95	06/27/18	28.64
06/28/18 06:00	28.75	06/28/18	28.54
06/29/18 06:00	28.73	06/29/18	27.90
06/30/18 06:00	28.78	06/30/18	28.02
07/01/18 06:00	28.90	07/01/18	28.29
07/02/18 06:00	28.85	07/02/18	28.40

07/03/18 06:00	28.95	07/03/18	28.47
07/04/18 06:00	29.35	07/04/18	28.74
07/05/18 06:00	29.03	07/05/18	28.68
07/06/18 06:00	28.61	07/06/18	28.80
07/07/18 06:00	28.61	07/07/18	28.77
07/08/18 06:00	28.66	07/08/18	28.39
07/09/18 06:00	28.58	07/09/18	28.70
07/10/18 06:00	28.66	07/10/18	28.47
07/11/18 06:00	28.75	07/11/18	28.27
07/12/18 06:00	28.80	07/12/18	28.56
07/13/18 06:00	29.00	07/13/18	28.51
07/14/18 06:00	28.98	07/14/18	28.55
07/15/18 06:00	28.98	07/15/18	28.43
07/16/18 06:00	28.90	07/16/18	28.91
07/17/18 06:00	29.13	07/17/18	28.90
07/18/18 06:00	29.13	07/18/18	29.12
07/19/18 06:00	29.15	07/19/18	28.96
07/20/18 06:00	29.20	07/20/18	28.38
07/21/18 06:00	29.33	07/21/18	29.21
07/22/18 06:00	29.38	07/22/18	29.14
07/23/18 06:00	29.43	07/23/18	28.69
07/24/18 06:00	29.55	07/24/18	28.83
07/25/18 06:00	29.65	07/25/18	29.21
07/26/18 06:00	30.20	07/26/18	29.61
07/27/18 06:00	29.50	07/27/18	29.64
07/28/18 06:00	29.83	07/28/18	29.91
07/29/18 06:00	30.05	07/29/18	29.79
07/30/18 06:00	29.75	07/30/18	29.77
07/31/18 06:00	29.75	07/31/18	29.67
08/01/18 06:00	29.73	08/01/18	28.97
08/02/18 06:00	29.75	08/02/18	29.71
08/03/18 06:00	29.45	08/03/18	29.48
08/04/18 06:00	29.35	08/04/18	29.52
08/05/18 06:00	29.35	08/05/18	29.40
08/06/18 06:00	29.45	08/06/18	29.39
08/07/18 06:00	29.75	08/07/18	29.16
08/08/18 06:00	29.75	08/08/18	29.30
08/09/18 06:00	29.45	08/09/18	29.28
08/10/18 06:00	29.43	08/10/18	28.99
08/11/18 06:00	29.38	08/11/18	28.68
08/12/18 06:00	29.33	08/12/18	29.04
08/13/18 06:00	29.15	08/13/18	29.06
08/14/18 06:00	29.00	08/14/18	29.04
08/15/18 06:00	29.15	08/15/18	29.23
08/16/18 06:00	29.60	08/16/18	29.43

08/17/18 06:00	29.23	08/17/18	29.20
08/18/18 06:00	29.00	08/18/18	29.08
08/19/18 06:00	29.05	08/19/18	28.98
08/20/18 06:00	29.43	08/20/18	28.99
08/21/18 06:00	29.20	08/21/18	28.99
08/22/18 06:00	29.45	08/22/18	29.09
08/23/18 06:00	29.50	08/23/18	29.19
08/24/18 06:00	29.58	08/24/18	29.50
08/25/18 06:00	29.58	08/25/18	29.31
08/26/18 06:00	29.43	08/26/18	29.38
08/27/18 06:00	29.15	08/27/18	29.36
08/28/18 06:00	29.25	08/28/18	29.32
08/29/18 06:00	29.20	08/29/18	29.33
08/30/18 06:00	29.15	08/30/18	29.47
08/31/18 06:00	29.35	08/31/18	29.39
09/01/18 06:00	29.30	09/01/18	29.42
09/02/18 06:00	29.65	09/02/18	29.54
09/03/18 06:00	29.55	09/03/18	29.19
09/04/18 06:00	29.25	09/04/18	29.23
09/05/18 06:00	29.35	09/05/18	29.51
09/06/18 06:00	29.60	09/06/18	29.66
09/07/18 06:00	29.78	09/07/18	29.63
09/08/18 06:00	29.95	09/08/18	29.64
09/09/18 06:00	29.70	09/09/18	29.29
09/10/18 06:00	29.50	09/10/18	29.40
09/11/18 06:00	29.93	09/11/18	29.48
09/12/18 06:00	29.98	09/12/18	29.27
09/13/18 06:00	29.15	09/13/18	29.42
09/14/18 06:00	29.35	09/14/18	29.65
09/15/18 06:00	29.23	09/15/18	29.59
09/16/18 06:00	30.20	09/16/18	29.71
09/17/18 06:00	29.73	09/17/18	29.70
09/18/18 06:00	30.13	09/18/18	29.67
09/19/18 06:00	30.18	09/19/18	29.53
09/20/18 06:00	29.98	09/20/18	29.71
09/21/18 06:00	29.88	09/21/18	29.77
09/22/18 06:00	29.70	09/22/18	29.85
09/23/18 06:00	29.63	09/23/18	29.76
09/24/18 06:00	29.40	09/24/18	29.67
09/25/18 06:00	29.15	09/25/18	29.57
09/26/18 06:00	29.20	09/26/18	29.58
09/27/18 06:00	29.18	09/27/18	29.49
09/28/18 06:00	29.00	09/28/18	29.37
09/29/18 06:00	29.45	09/29/18	29.36
09/30/18 06:00	29.25	09/30/18	29.45

10/01/18 06:00	29.83	10/01/18	29.35
10/02/18 06:00	29.23	10/02/18	29.46
10/03/18 06:00	29.45	10/03/18	29.38
10/04/18 06:00	29.33	10/04/18	29.31
10/05/18 06:00	29.35	10/05/18	29.21
10/06/18 06:00	29.35	10/06/18	29.29
10/07/18 06:00	28.83	10/07/18	29.10
10/08/18 06:00	28.66	10/08/18	29.07
10/09/18 06:00	29.18	10/09/18	29.09
10/10/18 06:00	29.58	10/10/18	29.20
10/11/18 06:00	29.65	10/11/18	29.24
10/12/18 06:00	29.85	10/12/18	29.35
10/13/18 06:00	30.15	10/13/18	29.62
10/14/18 06:00	30.05	10/14/18	29.52
10/15/18 06:00	30.15	10/15/18	29.71
10/16/18 06:00	29.70	10/16/18	29.44
10/17/18 06:00	29.38	10/17/18	29.37
10/18/18 06:00	29.30	10/18/18	29.51
10/19/18 06:00	29.25	10/19/18	29.20
10/20/18 06:00	29.15	10/20/18	28.99
10/21/18 06:00	29.05	10/21/18	29.03
10/22/18 06:00	29.25	10/22/18	29.01
10/23/18 06:00	29.28	10/23/18	29.01
10/24/18 06:00	29.40	10/24/18	29.01
10/25/18 06:00	29.43	10/25/18	29.15
10/26/18 06:00	29.50	10/26/18	29.05
10/27/18 06:00	29.55	10/27/18	29.10
10/28/18 06:00	29.25	10/28/18	29.26
10/29/18 06:00	29.15	10/29/18	29.11
10/30/18 06:00	28.75	10/30/18	28.92
10/31/18 06:00	29.03	10/31/18	28.98
11/01/18 06:00	28.98	11/01/18	28.89
11/02/18 06:00	28.75	11/02/18	29.18
11/03/18 06:00	29.05	11/03/18	28.97
11/04/18 06:00	29.05	11/04/18	28.75
11/05/18 06:00	28.93	11/05/18	28.76
11/06/18 06:00	29.05	11/06/18	28.90
11/07/18 06:00	29.00	11/07/18	28.88
11/08/18 06:00	29.08	11/08/18	28.90
11/09/18 06:00	28.95	11/09/18	28.81
11/10/18 06:00	29.03	11/10/18	28.90
11/11/18 06:00	29.55	11/11/18	28.94
11/12/18 06:00	28.95	11/12/18	28.92
11/13/18 06:00	28.66	11/13/18	28.85
11/14/18 06:00	28.90	11/14/18	28.90

11/15/18 06:00	26.59	11/15/18	28.53
11/16/18 06:00	27.99	11/16/18	28.63
11/17/18 06:00	27.99	11/17/18	28.38
11/18/18 06:00	28.36	11/18/18	28.33
11/19/18 06:00	28.46	11/19/18	28.17
11/20/18 06:00	28.56	11/20/18	28.16
11/21/18 06:00	28.46	11/21/18	28.14
11/22/18 06:00	28.48	11/22/18	28.12
11/23/18 06:00	28.51	11/23/18	28.21
11/24/18 06:00	28.26	11/24/18	28.22
11/25/18 06:00	28.38	11/25/18	28.21
11/26/18 06:00	28.16	11/26/18	28.28
11/27/18 06:00	28.46	11/27/18	28.27
11/28/18 06:00	27.37	11/28/18	28.14
11/29/18 06:00	27.99	11/29/18	27.95
11/30/18 06:00	27.96	11/30/18	28.07
12/01/18 06:00	27.86	12/01/18	27.98
12/02/18 06:00	27.86	12/02/18	27.92
12/03/18 06:00	27.96	12/03/18	27.93
12/04/18 06:00	27.86	12/04/18	27.91
12/05/18 06:00	28.13	12/05/18	27.91
12/06/18 06:00	28.11	12/06/18	28.00
12/07/18 06:00	27.94	12/07/18	27.93
12/08/18 06:00	28.04	12/08/18	27.87
12/09/18 06:00	27.57	12/09/18	27.87
12/10/18 06:00	28.16	12/10/18	27.97
12/11/18 06:00	26.02	12/11/18	27.76
12/12/18 06:00	27.05	12/12/18	27.49
12/13/18 06:00	27.57	12/13/18	27.49
12/14/18 06:00	27.49	12/14/18	27.43
12/15/18 06:00	26.85	12/15/18	27.47
12/16/18 06:00	26.95	12/16/18	27.19
12/17/18 06:00	27.10	12/17/18	27.10
12/18/18 06:00	27.17	12/18/18	26.99
12/19/18 06:00	27.37	12/19/18	27.06
12/20/18 06:00	27.42	12/20/18	27.05
12/21/18 06:00	26.51	12/21/18	26.92
12/22/18 06:00	26.61	12/22/18	26.85
12/23/18 06:00	26.37	12/23/18	26.75
12/24/18 06:00	26.68	12/24/18	26.77
12/25/18 06:00	26.78	12/25/18	26.85
12/26/18 06:00	26.85	12/26/18	26.84
12/27/18 06:00	26.98	12/27/18	26.91
12/28/18 06:00	26.90	12/28/18	27.01
12/29/18 06:00	27.08	12/29/18	26.92

	12/30/18 06:00	27.08	12/30/18	27.05
	12/31/18 06:00	27.08	12/31/18	27.15
2019	01/01/19 06:00	27.16	01/01/19	27.11
	01/02/19 06:00	27.08	01/02/19	26.96
	01/03/19 06:00	27.00	01/03/19	27.07
	01/04/19 06:00	26.93	01/04/19	26.89
	01/05/19 06:00	27.25	01/05/19	27.08
	01/06/19 06:00	27.22	01/06/19	27.05
	01/07/19 06:00	26.98	01/07/19	26.97
	01/08/19 06:00	26.78	01/08/19	26.94
	01/09/19 06:00	26.88	01/09/19	26.96
	01/10/19 06:00	26.39	01/10/19	26.90
	01/11/19 06:00	26.88	01/11/19	26.91
	01/12/19 06:00	27.08	01/12/19	26.85
	01/13/19 06:00	26.88	01/13/19	26.91
	01/14/19 06:00	27.17	01/14/19	26.94
	01/15/19 06:00	27.17	01/15/19	26.89
	01/16/19 06:00	27.05	01/16/19	26.75
	01/17/19 06:00	26.98	01/17/19	26.78
	01/18/19 06:00	26.98	01/18/19	26.71
	01/19/19 06:00	27.17	01/19/19	26.72
	01/20/19 06:00	27.15	01/20/19	26.71
	01/21/19 06:00	26.15	01/21/19	26.56
	01/22/19 06:00	26.19	01/22/19	26.54
	01/23/19 06:00	26.71	01/23/19	26.58
	01/24/19 06:00	26.78	01/24/19	26.55
	01/25/19 06:00	26.59	01/25/19	26.62
	01/26/19 06:00	26.59	01/26/19	26.49
	01/27/19 06:00	26.98	01/27/19	26.64
	01/28/19 06:00	25.61	01/28/19	26.41
	01/29/19 06:00	26.68	01/29/19	26.40
	01/30/19 06:00	26.88	01/30/19	26.41
	01/31/19 06:00	26.88	01/31/19	26.51
	02/01/19 06:00	26.76	02/01/19	26.49
	02/02/19 06:00	26.78	02/02/19	26.64
	02/03/19 06:00	26.68	02/03/19	26.64
	02/04/19 06:00	26.49	02/04/19	26.69
	02/05/19 06:00	26.49	02/05/19	26.77
	02/06/19 06:00	26.68	02/06/19	26.74
	02/07/19 06:00	26.37	02/07/19	26.72
	02/08/19 06:00	26.59	02/08/19	26.73
	02/09/19 06:00	26.59	02/09/19	26.72
	02/10/19 06:00	26.59	02/10/19	26.65
	02/11/19 06:00	26.78	02/11/19	26.65
	02/12/19 06:00	26.88	02/12/19	26.55

02/13/19 06:00	26.83	02/13/19	26.66
02/14/19 06:00	26.63	02/14/19	26.57
02/15/19 06:00	26.88	02/15/19	26.68
02/16/19 06:00	26.78	02/16/19	26.83
02/17/19 06:00	26.93	02/17/19	26.93
02/18/19 06:00	27.05	02/18/19	27.16
02/19/19 06:00	27.08	02/19/19	27.04
02/20/19 06:00	26.81	02/20/19	27.02
02/21/19 06:00	26.95	02/21/19	26.86
02/22/19 06:00	26.98	02/22/19	26.85
02/23/19 06:00	26.88	02/23/19	26.84
02/24/19 06:00	26.88	02/24/19	26.86
02/25/19 06:00	26.98	02/25/19	27.02
02/26/19 06:00	26.95	02/26/19	27.10
02/27/19 06:00	27.10	02/27/19	27.08
02/28/19 06:00	27.08	02/28/19	26.99
03/01/19 06:00	26.98	03/01/19	26.92
03/02/19 06:00	26.93	03/02/19	26.97
03/03/19 06:00	26.83	03/03/19	27.03
03/04/19 06:00	27.03	03/04/19	26.89
03/05/19 06:00	26.93	03/05/19	27.36
03/06/19 06:00	27.74	03/06/19	27.38
03/07/19 06:00	27.37	03/07/19	27.09
03/08/19 06:00	27.12	03/08/19	26.84
03/09/19 06:00	26.90	03/09/19	27.04
03/10/19 06:00	27.17	03/10/19	27.18
03/11/19 06:00	27.00	03/11/19	27.09
03/12/19 06:00	26.98	03/12/19	27.04
03/13/19 06:00	27.10	03/13/19	27.09
03/14/19 06:00	27.08	03/14/19	27.07
03/15/19 06:00	27.12	03/15/19	27.18
03/16/19 06:00	27.17	03/16/19	27.19
03/17/19 06:00	27.47	03/17/19	27.34
03/18/19 06:00	27.47	03/18/19	27.52
03/19/19 06:00	27.42	03/19/19	27.54
03/20/19 06:00	27.35	03/20/19	27.62
03/21/19 06:00	27.44	03/21/19	27.67
03/22/19 06:00	26.81	03/22/19	27.45
03/23/19 06:00	26.93	03/23/19	27.14
03/24/19 06:00	26.88	03/24/19	27.13
03/25/19 06:00	27.37	03/25/19	27.28
03/26/19 06:00	27.86	03/26/19	27.53
03/27/19 06:00	27.67	03/27/19	27.44
03/28/19 06:00	27.08	03/28/19	27.24
03/29/19 06:00	27.17	03/29/19	27.05

03/30/19 06:00	27.17	03/30/19	27.12
03/31/19 06:00	27.08	03/31/19	26.90
04/01/19 06:00	27.47	04/01/19	27.23
04/02/19 06:00	27.42	04/02/19	27.49
04/03/19 06:00	27.76	04/03/19	27.46
04/04/19 06:00	27.67	04/04/19	27.45
04/05/19 06:00	27.52	04/05/19	27.10
04/06/19 06:00	27.59	04/06/19	27.61
04/07/19 06:00	27.71	04/07/19	27.36
04/08/19 06:00	27.57	04/08/19	27.18
04/09/19 06:00	27.57	04/09/19	27.18
04/10/19 06:00	27.17	04/10/19	26.65
04/11/19 06:00	27.27	04/11/19	27.71
04/12/19 06:00	27.59	04/12/19	27.78
04/13/19 06:00	27.71	04/13/19	27.77
04/14/19 06:00	27.86	04/14/19	27.83
04/15/19 06:00	28.21	04/15/19	28.04
04/16/19 06:00	27.99	04/16/19	27.84
04/17/19 06:00	28.06	04/17/19	27.89
04/18/19 06:00	27.99	04/18/19	28.04
04/19/19 06:00	28.06	04/19/19	28.03
04/20/19 06:00	26.37	04/20/19	27.59
04/21/19 06:00	27.27	04/21/19	27.53
04/22/19 06:00	27.47	04/22/19	27.67
04/23/19 06:00	27.49	04/23/19	27.64
04/24/19 06:00	28.06	04/24/19	27.72
04/25/19 06:00	27.94	04/25/19	27.62
04/26/19 06:00	27.86	04/26/19	27.61
04/27/19 06:00	27.79	04/27/19	27.82
04/28/19 06:00	27.86	04/28/19	27.92
04/29/19 06:00	28.56	04/29/19	28.12
04/30/19 06:00	28.16	04/30/19	28.15
05/01/19 06:00	28.11	05/01/19	28.09
05/02/19 06:00	28.41	05/02/19	28.03
05/03/19 06:00	28.43	05/03/19	27.72
05/04/19 06:00	28.33	05/04/19	27.86
05/05/19 06:00	28.31	05/05/19	28.05
05/06/19 06:00	28.31	05/06/19	27.97
05/07/19 06:00	28.21	05/07/19	28.16
05/08/19 06:00	28.46	05/08/19	28.21
05/09/19 06:00	28.51	05/09/19	28.21
05/10/19 06:00	28.46	05/10/19	28.11
05/11/19 06:00	28.46	05/11/19	27.95
05/12/19 06:00	28.38	05/12/19	27.87
05/13/19 06:00	28.46	05/13/19	28.03

05/14/19 06:00	28.66	05/14/19	27.85
05/15/19 06:00	28.75	05/15/19	28.10
05/16/19 06:00	28.85	05/16/19	28.23
05/17/19 06:00	29.00	05/17/19	28.25
05/18/19 06:00	29.03	05/18/19	28.21
05/19/19 06:00	28.93	05/19/19	28.19
05/20/19 06:00	28.90	05/20/19	28.31
05/21/19 06:00	28.78	05/21/19	28.46
05/22/19 06:00	28.75	05/22/19	28.52
05/23/19 06:00	28.66	05/23/19	28.73
05/24/19 06:00	28.66	05/24/19	28.63
05/25/19 06:00	28.78	05/25/19	28.63
05/26/19 06:00	28.85	05/26/19	28.77
05/27/19 06:00	28.95	05/27/19	28.95
05/28/19 06:00	29.20	05/28/19	28.98
05/29/19 06:00	29.25	05/29/19	29.13
05/30/19 06:00	29.55	05/30/19	29.37
05/31/19 06:00	29.60	05/31/19	29.44
06/01/19 06:00	29.50	06/01/19	29.21
06/02/19 06:00	29.15	06/02/19	29.44
06/03/19 06:00	29.15	06/03/19	28.83
06/04/19 06:00	29.08	06/04/19	29.19
06/05/19 06:00	29.05	06/05/19	29.09
06/06/19 06:00	28.95	06/06/19	28.99
06/07/19 06:00	28.93	06/07/19	28.91
06/08/19 06:00	29.15	06/08/19	29.00
06/09/19 06:00	29.35	06/09/19	29.23
06/10/19 06:00	29.33	06/10/19	28.92
06/11/19 06:00	29.55	06/11/19	29.16
06/12/19 06:00	29.35	06/12/19	29.20
06/13/19 06:00	29.35	06/13/19	29.46
06/14/19 06:00	29.25	06/14/19	29.26
06/15/19 06:00	28.70	06/15/19	29.20
06/16/19 06:00	29.00	06/16/19	29.27
06/17/19 06:00	29.10	06/17/19	29.22
06/18/19 06:00	29.23	06/18/19	29.06
06/19/19 06:00	29.13	06/19/19	29.16
06/20/19 06:00	29.15	06/20/19	29.05
06/21/19 06:00	29.20	06/21/19	29.10
06/22/19 06:00	29.38	06/22/19	29.04
06/23/19 06:00	29.33	06/23/19	28.80
06/24/19 06:00	29.20	06/24/19	28.90
06/25/19 06:00	29.15	06/25/19	28.84
06/26/19 06:00	29.15	06/26/19	28.97
06/27/19 06:00	29.25	06/27/19	28.99

06/28/19 06:00	29.28	06/28/19	29.23
06/29/19 06:00	29.45	06/29/19	29.27
06/30/19 06:00	29.70	06/30/19	29.06
