# Supplementary material for: Coral Gardens Reef, Belize: An Acropora spp. refugium under threat in a warming world
Source: PLoS One. 2023 Feb 8;18(2):e0280852. doi: 10.1371/journal.pone.0280852 (PMC9907857; doi:10.1371/journal.pone.0280852)
Supplement: S4 Table — (PDF) [file pone.0280852.s004.pdf]

Table S4. Sediment thickness data from Coral Gardens from 2014.

2019 Sed Thickness (cm)

	Mean (cm)	1.4	3.85
Sample #	Site Location	Min (cm)	Max (cm)
1	5E	0	0
2	5N	0	0.5
3	5W	3	10
4	5S	2	3
5	N Side Patch	1	5
6	2E	3	8
7	1E	5	8
8	1W	0	2
9	1N	0	1
10	1S	0	1
