# Supplementary material for: Coral Gardens Reef, Belize: An Acropora spp. refugium under threat in a warming world
Source: PLoS One. 2023 Feb 8;18(2):e0280852. doi: 10.1371/journal.pone.0280852 (PMC9907857; doi:10.1371/journal.pone.0280852)
Supplement: S5 Table — (PDF) [file pone.0280852.s005.pdf]

Table S5. Coral canopy height data from transects 1 and 5 at Coral Gardens in 2014.

2014 Coral Canopy Height (m)

Max	3.9	4.3
Min	2.8	3.5
Mean	3.3	4.0
**Transect 1**	Canopy Height	Depth to Hardground
1	3.5	3.8
2	3.2	
3	2.75	
4	2.75	
5	3.4	
6	3.45	4.2
7	3.25	
8	3.25	
9	3.5	
10	3.5	
11	3.5	4.1
12	3.55	
13	3.6	
14	3.25	
15	2.85	
16	3	4
17	2.75	
18	3.9	
19	2.9	
20	3	
21	3.4	4.2
22	3.2	
23	3.25	
24	3.5	
25	3.5	
26	3.5	4.3
27	3.5	
28	3.5	
29	3.55	
30	3.25	
31	3	3.5
32	3.2	
33		
34		
35		
36		
37		

2014 Coral Canopy Height (m)

Max	2.8	3.9
Min	1.7	2.8
Mean	2.2	3.3
**Transect 5**	Canopy Height	Depth to Hardground
1	2.5	3.7
2	2.75	
3	2.25	
4	2	
5	2	2.8
6	2.1	
7	2	
8	1.8	
9	1.7	
10	1.75	2.95
11	1.75	
12	1.75	
13	1.7	
14	1.7	
15	1.9	2.8
16	2	
17	2	
18	2	
19	2	
20	2.2	3.45
21	2.2	
22	2.25	
23	2.2	
24	2.1	
25	2.05	3.4
26	2	
27	2.1	
28	2.05	
29	2	
30	1.95	3.25
31	2.05	
32	2.4	
33	2.45	
34	2.5	
35	2.75	3.85
36	3.35	
37	3.65	
