# Supplementary material for: Coral Gardens Reef, Belize: An Acropora spp. refugium under threat in a warming world
Source: PLoS One. 2023 Feb 8;18(2):e0280852. doi: 10.1371/journal.pone.0280852 (PMC9907857; doi:10.1371/journal.pone.0280852)
Supplement: S6 Table — (PDF) [file pone.0280852.s006.pdf]

Table S6. Urchin abundance data (n) per quadrat at Coral Gardens in 2014 and 2019.

2014 **Mean of Means: 20.4**

Mean	29.98	14.39	20.87	36.46	0.42
Year	2014	2014	2014	2014	2014
Transect	T1	T2	T3	T4	T5
Quadrat #	Urchin	Urchin	Urchin	Urchin	Urchin
1	1.5	10	0	10	0
2	23	14	5	7.5	1
3	28.5	13	26	5.5	0
4	27.5	10	38	26	0.5
5	24	16	26.5	57.5	0
6	47	24.5	37.5	46	1
7	48	19.5	22.5	60	1
8	45	3	16.5	57	1.5
9	48.5	37	20	42	0.5
10	51	23.5	21.5	46	2.5
11	42	20.5	21.5	38	1.5
12	36	22	31	57.5	2
13	34	26	11	37	1
14	24.5	23	15	20.5	0
15	16	23	31.5		0
16	19	31.5	19.5		0
17	21.5	16.5	17		0.5
18	46.5	10	20		0
19	17.5	13	16.5		1.5
20	7	6.5			0
21	9.5	4			0
22	16.5	4.5			0
23	36	6.5			0
24	44.5	11.5			0
25	43.5	15			0
26	38.5	16.5			0
27	20.5	7.5			0
28	25.5	3			0
29	16	0			0
30	32	5.5			0
31	36.5	9.5			0
32	32.5				0
33					1
34					0
35					0
36					0
37					0

2019 **Mean of Means: 24.1**

Mean	26.60	23.48	35.05	34.00	1.61
Year	2019	2019	2019	2019	2019
Transect	T1	T2	T3	T4	T5
Quadrat #	Urchin	Urchin	Urchin	Urchin	Urchin
1	4.5	0	1	4	1
2	0	3.5	2	5	0
3	2	11	21	33	0
4	4	32	26.5	41	0
5	3	22	37	46	0
6	41	20	48.5	52	1
7	20	19.5	45.5	70.5	1
8	15.5	13	32.5	70.5	0
9	13.5	5	16.5	56	2
10	13.5	3	37.5	36	0.5
11	36.5	2.5	66.5	37	2
12	35	7	38	37	1
13	40	21	58	8	2
14	30	49.5	43	2	2.5
15	30	43.5	44	12	1
16	48	25	55.5		2.5
17	43	29.5	42.5		0
18	42.5	43.5	45		1
19	43.5	40	34		0.5
20	29.5	45	30.5		1.5
21	36	47	11		2
22	49	40.5			3
23	40	44			5
24	26	36			4
25	32.5	2			2.5
26	21	37.5			3.5
27	37.5	16.5			3.5
28	30	17.5			2
29	28	34.5			6
30	48.5	11.5			2.5
31	32	5.5			1.5
32	38				1
33	25.5				0.5
34	14.5				0.5
35	2				1
36	2				1
37					0.5
