# Supplementary material for: Coral Gardens Reef, Belize: An Acropora spp. refugium under threat in a warming world
Source: PLoS One. 2023 Feb 8;18(2):e0280852. doi: 10.1371/journal.pone.0280852 (PMC9907857; doi:10.1371/journal.pone.0280852)
Supplement: S7 Table — (PDF) [file pone.0280852.s007.pdf]

Table S7. Damselfish abundance data (n per quadrat) at Coral Gardens in 2014 and 2019.

2014 **Mean: 2.0**

Mean	2.51	1.94	2.50	2.31	1.21
Year	2014	2014	2014	2014	2014
Transect	T1	T2	T3	T4	T5
Quadrat #	Damsel	Damsel	Damsel	Damsel	Damsel
1	1	1	2	0	0
2	2	3	0	0	0
3	5	1	2	0	1
4	4	2	4	2	1
5	2	3	3	3	0
6	3	1	5	4	1
7	3	3	4	5	1
8	1	0	2	1	2
9	4	3	3	4	2
10	3	3	3	2	1
11	4	5	4	2	1
12	2	1	3	3	1
13	3	4	2	4	1
14	4	2	1		3
15	3	4	1		1
16	4	3	2		2
17	3	0	2		1
18	2	3	3		1
19	4	3	3		1
20	2	1	1		2
21	1	2			1
22	3	1			2
23	3	1			2
24	4	0			1
25	3	1			0
26	2	2			1
27	3	1			2
28	3	2			2
29	3	1			1
30	2	1			2
31	2	2			1
32	1				3
33	1				1
34	2				1
35	2				2
36	1				1
37	1				0
38	2				0
39	0				

2019 **Mean: 2.3**

Mean	2.78	2.47	2.30	2.03	1.84
Year	2019	2019	2019	2019	2019
Transect	T1	T2	T3	T4	T5
Quadrat #	Damsel	Damsel	Damsel	Damsel	Damsel
1	0	3	0	0	1.5
2	0	2	0	0	2
3	0	3	3	2	2
4	0	1	3	2	1
5	2	1.5	3	3.5	2
6	1	2	2	3	2
7	0	2	3	3	2
8	1	2	3	2.5	2
9	1	1	2.5	2.5	2
10	1	1	2	2.5	1
11	1	3	2	2.5	1.5
12	2	2	1.5	2.5	2
13	3	3	2	2.5	1
14	4	3	3	2	1
15	5	3	3	0	2
16	7	2.5	2		2
17	5.5	3.5	2.5		3
18	5.5	3.5	3		2
19	4	3.5	3		2
20	4.5	3	2.5		2
21	4	2.5			1
22	3	4.5			1
23	4	2			1.5
24	5	2			3
25	4	3			2
26	4	4			3
27	2.5	3.5			2
28	4	2			1.5
29	2.5	2			2
30	1	0			2.5
31	2	0			3.5
32	3.5				2
33	3.5				2.5
34	3.5				3
35	3				2
36	2				3
37	3.5				2
38	3				0
39					0
