# Supplementary material for: Coral Gardens Reef, Belize: An Acropora spp. refugium under threat in a warming world
Source: PLoS One. 2023 Feb 8;18(2):e0280852. doi: 10.1371/journal.pone.0280852 (PMC9907857; doi:10.1371/journal.pone.0280852)
Supplement: S8 Table — (PDF) [file pone.0280852.s008.pdf]

Table S8. Substrate point count data at Coral Gardens in 2014 and 2019.

**2014 Point Count at T1**		
ID	*N*	*%*
Sediment	1	1.5
Bare Rock (dead coral)	24	36.4
Macroalgae	4	6.1
Acropora cervicornis	29	43.9
Porites astreoides	2	3.0
Agaricia agaracites	0	0.0
Millepora complenata	6	9.1
Porites porites	0	0.0
Millepora alcicornis	0	0.0
Orbicella annularis	0	0.0
Orbicella faveolata	0	0.0
Gorgonia ventalina	0	0.0
Agaricia tenufolia	0	0.0
Plexaurella	0	0.0
Porites divericata	0	0.0

**2014 Point Count at T2**		
ID	*N*	*%*
Sediment	1	1.5
Bare Rock (dead coral)	14	20.9
Macroalgae	3	4.5
Acropora cervicornis	43	64.2
Porites astreoides	1	1.5
Agaricia agaracites	0	0.0
Millepora complenata	2	3.0
Porites porites	1	1.5
Millepora alcicornis	0	0.0
Orbicella annularis	0	0.0
Orbicella faveolata	0	0.0
Gorgonia ventalina	2	3.0
Agaricia tenufolia	0	0.0
Plexaurella	0	0.0
Porites divericata	0	0.0

**2014 Point Count at T3**		
ID	*N*	*%*
Sediment	3	7.3
Bare Rock (dead coral)	11	26.8
Macroalgae	1	2.4
Acropora cervicornis	26	63.4
Porites astreoides	0	0.0
Agaricia agaracites	0	0.0
Millepora complenata	0	0.0
Porites porites	0	0.0
Millepora alcicornis	0	0.0
Orbicella annularis	0	0.0
Orbicella faveolata	0	0.0
Gorgonia ventalina	0	0.0
Agaricia tenufolia	0	0.0
Plexaurella	0	0.0
Porites divericata	0	0.0

**2014 Point Count at T4**		
ID	*N*	*%*
Sediment	1	3.1
Bare Rock (dead coral)	10	31.3
Macroalgae	2	6.3
Acropora cervicornis	15	46.9
Porites astreoides	0	0.0
Agaricia agaracites	0	0.0
Millepora complenata	1	3.1
Porites porites	0	0.0
Millepora alcicornis	0	0.0
Orbicella annularis	2	6.3
Orbicella faveolata	0	0.0
Gorgonia ventalina	0	0.0
Agaricia tenufolia	0	0.0
Plexaurella	0	0.0
Porites divericata	1	3.1

**2014 Point Count at T5**		
ID	*N*	*%*
Sediment	5	6.4
Bare Rock (dead coral)	8	10.3
Macroalgae	6	7.7
Acropora cervicornis	59	75.6
Porites astreoides	0	0.0
Agaricia agaracites	0	0.0
Millepora complenata	0	0.0
Porites porites	0	0.0
Millepora alcicornis	0	0.0
Orbicella annularis	0	0.0
Orbicella faveolata	0	0.0
Gorgonia ventalina	0	0.0
Agaricia tenufolia	0	0.0
Plexaurella	0	0.0
Porites divericata	0	0.0

**2019 Point Count at T1**		
ID	*N*	*%*
Sediment	4	6.3
Bare Rock (dead coral)	18	28.6
Macroalgae	3	4.8
Acropora cervicornis	20	31.7
Porites astreoides	3	4.8
Agaricia agaracites	9	14.3
Millepora complenata	1	1.6
Porites porites	2	3.2
Millepora alcicornis	1	1.6
Orbicella annularis	0	0.0
Orbicella faveolata	0	0.0
Gorgonia ventalina	0	0.0
Agaricia tenufolia	0	0.0
Plexaurella	2	3.2
Porites divericata	0	0.0

**2019 Point Count at T2**		
ID	*N*	*%*
Sediment	1	1.6
Bare Rock (dead coral)	23	35.9
Macroalgae	6	9.4
Acropora cervicornis	24	37.5
Porites astreoides	0	0.0
Agaricia agaracites	3	4.7
Millepora complenata	0	0.0
Porites porites	0	0.0
Millepora alcicornis	0	0.0
Orbicella annularis	0	0.0
Orbicella faveolata	0	0.0
Gorgonia ventalina	1	1.6
Agaricia tenufolia	0	0.0
Plexaurella	6	9.4
Porites divericata	0	0.0

**2019 Point Count at T3**		
ID	*N*	*%*
Sediment	1	2.1
Bare Rock (dead coral)	21	44.7
Macroalgae	1	2.1
Acropora cervicornis	14	29.8
Porites astreoides	0	0.0
Agaricia agaracites	4	8.5
Millepora complenata	0	0.0
Porites porites	2	4.3
Millepora alcicornis	0	0.0
Orbicella annularis	0	0.0
Orbicella faveolata	0	0.0
Gorgonia ventalina	0	0.0
Agaricia tenufolia	4	8.5
Plexaurella	0	0.0
Porites divericata	0	0.0

**2019 Point Count at T4**		
ID	*N*	*%*
Sediment	5	15.6
Bare Rock (dead coral)	8	25.0
Macroalgae	0	0.0
Acropora cervicornis	15	46.9
Porites astreoides	0	0.0
Agaricia agaracites	2	6.3
Millepora complenata	1	3.1
Porites porites	0	0.0
Millepora alcicornis	0	0.0
Orbicella annularis	1	3.1
Orbicella faveolata	0	0.0
Gorgonia ventalina	0	0.0
Agaricia tenufolia	0	0.0
Plexaurella	0	0.0
Porites divericata	0	0.0

**2019 Point Count at T5**		
ID	*N*	*%*
Sediment	0	0.0
Bare Rock (dead coral)	32	41.0
Macroalgae	5	6.4
Acropora cervicornis	39	50.0
Porites astreoides	1	1.3
Agaricia agaracites	0	0.0
Millepora complenata	0	0.0
Porites porites	0	0.0
Millepora alcicornis	0	0.0
Orbicella annularis	0	0.0
Orbicella faveolata	1	1.3
Gorgonia ventalina	0	0.0
Agaricia tenufolia	0	0.0
Plexaurella	0	0.0
Porites divericata	0	0.0
