# Supplementary material for: Coral Gardens Reef, Belize: An Acropora spp. refugium under threat in a warming world
Source: PLoS One. 2023 Feb 8;18(2):e0280852. doi: 10.1371/journal.pone.0280852 (PMC9907857; doi:10.1371/journal.pone.0280852)
Supplement: S9 Table — (PDF) [file pone.0280852.s009.pdf]

Table S9. MC ICP-MS ^{230}Th age data for 35 dead coral samples collected from Coral Gardens, Belize (from Greer et al., 2020).

Sample name	Sample location	Sample wt.(g)	U (ppm)	^{232}Th (ppb)	(^{230}Th/^{232}Th)	(^{230}Th/ ^{238}U)	Corr. δ^{238}U[1]	Uncorr. ^{230}Th Age (ka)	Date of chemistry	Corr. year (AD)[2]
T5Sb-BZ-CG-TW15	Modern Canopy	0.18573	3.2080 ± 0.0028	0.05258 ± 0.00029	52.09 ± 0.91	0.0002814 ± 0.0000047	148.6 ± 1.1	0.02671 ± 0.00044	2015.58	1993.6 ± 1.0
T5Sd-BZ-CG-TW15	Modern Canopy	0.27624	3.2696 ± 0.0019	0.06616 ± 0.00042	53.54 ± 1.02	0.0003571 ± 0.0000064	146.4 ± 1.2	0.03397 ± 0.00061	2015.58	1986.3 ± 1.1
A2a-BZ-CG-TW15	Pit C	0.20141	3.4614 ± 0.0015	0.13827 ± 0.00025	18.91 ± 0.49	0.0002490 ± 0.0000064	148.6 ± 0.9	0.02365 ± 0.00061	2015.58	1996.8 ± 1.2
A6a-BZ-CG-TW15	Pit C	0.16644	3.4903 ± 0.0015	0.14287 ± 0.00028	26.51 ± 0.40	0.0003576 ± 0.0000053	146.3 ± 1.2	0.03402 ± 0.00051	2015.58	1986.4 ± 1.1
F10c_BZ-CG-TW15	Modern Canopy	0.15188	3.2473 ± 0.0025	0.01524 ± 0.00014	60.30 ± 1.72	0.0000933 ± 0.0000025	150.5 ± 0.8	0.00884 ± 0.00024	2015.84	2011.4 ± 0.9
F10a_BZ-CG-TW15	Modern Canopy	0.1522	3.1943 ± 0.0015	0.01717 ± 0.00024	60.25 ± 1.57	0.0001067 ± 0.0000024	148.1 ± 1.3	0.01014 ± 0.00023	2015.84	2010.2 ± 0.9
T5Nc_BZ-CG-TW15	Modern Canopy	0.15589	3.0778 ± 0.0019	0.06231 ± 0.00016	17.46 ± 0.38	0.0001165 ± 0.0000025	148.1 ± 1.2	0.01106 ± 0.00024	2015.84	2009.7 ± 1.0
F8a_BZ-CG-TW15	Modern Canopy	0.15505	3.2975 ± 0.0018	0.02876 ± 0.00020	40.39 ± 1.07	0.0001161 ± 0.0000030	147.3 ± 1.3	0.01104 ± 0.00028	2015.84	2009.2 ± 0.9
F3a_BZ-CG-TW15	Modern Canopy	0.15345	3.4656 ± 0.0021	0.12088 ± 0.00022	10.64 ± 0.28	0.0001223 ± 0.0000032	146.9 ± 1.1	0.01163 ± 0.00030	2015.84	2008.9 ± 1.0
T5Ed_BZ-CG-TW15	Modern Canopy	0.15781	3.1612 ± 0.0022	0.04637 ± 0.00017	26.31 ± 0.83	0.0001272 ± 0.0000040	146.6 ± 1.1	0.01210 ± 0.00038	2015.84	2008.5 ± 1.0
T5Ee_BZ-CG-TW15	Modern Canopy	0.15237	3.1812 ± 0.0019	0.06522 ± 0.00015	19.14 ± 0.49	0.0001294 ± 0.0000033	147.8 ± 1.1	0.01229 ± 0.00031	2015.84	2008.4 ± 1.0
T5Ef_BZ-CG-TW15	Modern Canopy	0.15232	3.0888 ± 0.0022	0.08873 ± 0.00026	14.03 ± 0.31	0.0001328 ± 0.0000029	149.2 ± 1.1	0.01260 ± 0.00028	2015.84	2008.3 ± 1.1
T5Eb_BZ-CG-TW15	Modern Canopy	0.15289	3.1770 ± 0.0020	0.07202 ± 0.00018	21.20 ± 0.59	0.0001584 ± 0.0000044	149.8 ± 1.2	0.01502 ± 0.00042	2015.84	2005.7 ± 1.1
F9a_BZ-CG-TW15	Modern Canopy	0.16957	3.2427 ± 0.0017	0.03285 ± 0.00021	48.59 ± 1.15	0.0001622 ± 0.0000037	148.2 ± 0.9	0.01541 ± 0.00035	2015.84	2005.0 ± 1.0
T5Wa_BZ-CG-TW15	Modern Canopy	0.15424	3.1664 ± 0.0015	0.14451 ± 0.00042	12.50 ± 0.25	0.0001880 ± 0.0000038	150.2 ± 0.9	0.01783 ± 0.00036	2015.84	2003.3 ± 1.1
T5Na_BZ-CG-TW15	Modern Canopy	0.16181	3.0933 ± 0.0020	0.19431 ± 0.00036	10.19 ± 0.23	0.0002109 ± 0.0000047	147.5 ± 1.3	0.02005 ± 0.00045	2015.84	2001.5 ± 1.2
F1a_BZ-CG-TW15	Modern Canopy	0.15291	3.1376 ± 0.0018	0.11831 ± 0.00028	21.01 ± 0.56	0.0002611 ± 0.0000070	146.3 ± 1.3	0.02484 ± 0.00067	2015.84	1996.2 ± 1.3
T5We_BZ-CG-TW15	Modern Canopy	0.16096	3.2549 ± 0.0019	0.09496 ± 0.00026	28.80 ± 0.61	0.0002769 ± 0.0000058	150.0 ± 0.8	0.02626 ± 0.00055	2015.84	1994.5 ± 1.1
T5Wc_BZ-CG-TW15	Modern Canopy	0.15198	3.2097 ± 0.0019	0.08761 ± 0.00030	30.89 ± 0.53	0.0002779 ± 0.0000047	148.4 ± 1.0	0.02639 ± 0.00044	2015.84	1994.4 ± 1.1
F1b_BZ-CG-TW15	Modern Canopy	0.15895	3.1918 ± 0.0015	0.14726 ± 0.00032	18.98 ± 0.36	0.0002887 ± 0.0000055	147.5 ± 1.0	0.02744 ± 0.00052	2015.84	1993.7 ± 1.2
A1b_BZ-CG-TW15	Pit C	0.15179	3.3969 ± 0.0030	0.14366 ± 0.00029	20.87 ± 0.48	0.0002909 ± 0.0000066	146.3 ± 1.4	0.02768 ± 0.00063	2015.84	1993.1 ± 1.2
T5Wb_BZ-CG-TW15	Modern Canopy	0.17223	3.2901 ± 0.0015	0.10452 ± 0.00036	28.52 ± 0.53	0.0002986 ± 0.0000054	147.9 ± 1.1	0.02837 ± 0.00052	2015.84	1992.4 ± 1.1
T5Sf_BZ-CG-TW15	Modern Canopy	0.15047	3.2681 ± 0.0012	0.26047 ± 0.00055	12.41 ± 0.22	0.0003259 ± 0.0000057	147.1 ± 0.9	0.03098 ± 0.00054	2015.84	1990.7 ± 1.3
T5Wd_BZ-CG-TW15	Modern Canopy	0.16769	3.2350 ± 0.0028	0.13326 ± 0.00030	25.06 ± 0.40	0.0003402 ± 0.0000054	148.1 ± 1.1	0.03232 ± 0.00051	2015.84	1988.7 ± 1.2
T5Sd_BZ-CG-TW15	Modern Canopy	0.15314	3.2571 ± 0.0036	0.06729 ± 0.00028	55.82 ± 1.09	0.0003801 ± 0.0000073	150.9 ± 1.3	0.03602 ± 0.00069	2015.84	1984.5 ± 1.2
T5Si_BZ-CG-TW15	Modern Canopy	0.1535	3.2507 ± 0.0030	0.26151 ± 0.00051	15.49 ± 0.22	0.0004107 ± 0.0000058	149.7 ± 1.0	0.03896 ± 0.00055	2015.84	1982.7 ± 1.3

Ratios in parentheses are activity ratios calculated from atomic ratios using ISOPLOT 3.75 (Ludwig 2012) using decay constants λ_{238}= 1.55125 × 10^{-10} yr^{-1} (Jaffey et al. 1971), λ_{234} = (2.8262±0.0057) × 10^{-6} yr^{-1}, λ_{230} = (9.158±0.028) × 10^{-6} yr^{-1} (Cheng et al. 2000). All values have been calculated after mean laboratory blank extraction. All errors reported in this table are quoted as 2σ. *Note the high ^{232}Th value for sample f6 and for D4a.

1. $\delta^{234}U(T)=\delta^{234}U(O)e^{\lambda 234T}$ where $\delta^{234}U=[(^{234}U/^{238}U)-1] \times 1000$

2. To account for both hydrogenous and terrestrially derived $^{230}Th_0$, the corrected (corr.) ^{230}Th age of each sample was calculated using a sample specific non-radiogenic (^{230}Th/^{232}Th) value using the following equation in Clark et al. (2014):

$$\left(\frac{^{230}Th}{^{232}Th}\right)_{mix} = \left(\left(\frac{^{232}Th_{live}}{^{232}Th_{dead}}\right) \times \left(\frac{^{230}Th}{^{232}Th}\right)_{live}\right) + \left(\left(\frac{^{232}Th_{dead} - ^{232}Th_{live}}{^{232}Th_{dead}}\right) \times \left(\frac{^{230}Th}{^{232}Th}\right)_{sed}\right)$$

where $^{232}Th_{dead}$ is the measured ^{232}Th value (ppb) in the individual dead coral sample. Hydrogenous and detrital values used in the equation reflect values obtained from *Porites* corals collected from the Palm Islands region, Great Barrier Reef (Clark et al. 2014), due to the unavailability of locally derived Belize coral sample values. $^{232}Th_{live}$ is the mean ^{232}Th value (ppb) measured in live *Porites* spp. coral samples collected from the Palm Islands, determined to be 0.95 ppb (N=12). $^{230}Th/^{232}Th_{live}$ represents or approximates the isotopic composition of the hydrogenous component in the live *Porites* spp. coral skeleton during growth with an activity value of 1.08 ± 20% (atomic ratio 5.85×10^{-6} ± 20%) $^{230}Th/^{232}Th_{sed}$ represents the detrital component incorporated into the coral skeleton post-mortem with a mean activity value of 0.61 ± 20% (atomic ratio 3.53 ×10^{-6} ± 20%) based on y-intercept values of ^{230}Th/^{232}Th versus ^{238}U/^{232}Th isochrons obtained from dead *Porites* collected from the Palm Islands.
