# Supplementary material for: Coral Gardens Reef, Belize: An Acropora spp. refugium under threat in a warming world
Source: PLoS One. 2023 Feb 8;18(2):e0280852. doi: 10.1371/journal.pone.0280852 (PMC9907857; doi:10.1371/journal.pone.0280852)

PLOS ONE

Coral Gardens Reef, Belize: An Acropora spp. refugium under threat in a warming world

--Manuscript Draft--

Manuscript Number:	PONE-D-22-24875
Article Type:	Research Article
Full Title:	Coral Gardens Reef, Belize: An Acropora spp. refugium under threat in a warming world
Short Title:	Coral Gardens Reef, Belize: An Acropora spp. refugium under threat
Corresponding Author:	Lisa Greer<br>Washington and Lee University<br>Lexington, Virginia UNITED STATES
Keywords:	Acropora; Belize; coral; coral reef; refugium; Caribbean; sea surface temperature; climate change; hurricanes
Abstract:	Live coral cover has declined precipitously on Caribbean reefs in recent years. *Acropora cervicornis* coral has been particularly decimated, and few Western Atlantic *Acropora* spp. refugia remain. Coral Gardens, Belize, was identified in 2020 as a long-term refugium for this species. This study assessed live *A. cervicornis* coral abundance over time at Coral Gardens to monitor the stability of *A. cervicornis* corals, and to explore potential threats to this important refugium. Live coral cover was documented annually from 2012-2019 along five permanent transects. *In situ* sea-surface temperature data were collected at Coral Gardens throughout the study period and data on bathymetry, sediment, substrate, herbivore abundance, and macroalgal abundance were collected in 2014 and 2019 to assess potential threats to Coral Gardens. Live coral cover declined at all five transect sites over the study period. The greatest loss of live coral occurred between 2016 and 2017, coincident with the earliest and highest warm-season temperatures recorded at the study site and the passage of a hurricane in 2016. Yet Degree Heating Weeks (DHW), a measure of threshold temperature for coral bleaching, did not accumulate during the study period, and structural storm damage was not observed at Coral Gardens. Uranium-thorium (230 Th) dating of 26 dead *in situ* fragments of *A. cervicornis* collected in 2015 from Coral Gardens further revealed no correlation between coral mortality and tropical storms and hurricanes in the recent past. Our data suggest that several other common drivers for coral decline (i.e. herbivory, predation, sedimentation, pH) may likely be ruled out for Coral Gardens. At the end of the study period, Coral Gardens satisfied most criteria for refugium status. However, the early onset, higher mean, and longer duration of above-average temperatures as well as intermittent temperature anomalies likely play a critical role in the stability of this refugium, even when DHW do not accumulate. We suggest that sub-lethal temperature stress in 2016 may have increased coral tissue vulnerability at Coral Gardens to a passing hurricane, threatening the status of this unique refugium.
Order of Authors:	Lisa Greer
	H. Allen Curran
	Karl Wirth
	Robert Humston
	Ginny Johnson
	Lauren McManus
	Candice Stefanic
	Tara Clark
	Halard Lescinsky
	Kirah Forman-Castillo

Powered by Editorial Manager® and ProduXion Manager® from Aries Systems Corporation

Additional Information:	
**Question**	**Response**
**Financial Disclosure**<br><br>Enter a financial disclosure statement that describes the sources of funding for the work included in this submission. Review the submission guidelines for detailed requirements. View published research articles from *PLOS ONE* for specific examples.<br><br>This statement is required for submission and **will appear in the published article** if the submission is accepted. Please make sure it is accurate.<br><br>**Unfunded studies**<br>Enter: *The author(s) received no specific funding for this work.*<br><br>**Funded studies**<br>Enter a statement with the following details:<br>• Initials of the authors who received each award<br>• Grant numbers awarded to each author<br>• The full name of each funder<br>• URL of each funder website<br>• Did the sponsors or funders play any role in the study design, data collection and analysis, decision to publish, or preparation of the manuscript?<br>• **NO** - Include this sentence at the end of your statement: *The funders had no role in study design, data collection and analysis, decision to publish, or preparation of the manuscript.*<br>• **YES** - Specify the role(s) played.<br><br>* typeset	This work was supported by the National Science Foundation and the Keck Geology Consortium under Grant No. NSF-REU #1358987 to LG, KW and HL and Grant No. NSF-REU #1659322 to LG and KW (https://keckgeology.org/). Support also was received from The Washington and Lee University: Johnson Opportunity Grant, Summer Research Scholar Program, Lenfest summer research grant, R. Preston Hawkins IV Award in Geology, and Department of Geology to LG, GJ, LM, CS, and ARC DECRA Fellowship support for TC (https://www.arc.gov.au/grants/discovery-program/discovery-early-career-researcher-award-decra). The funders had no role in study design, data collection and analysis, decision to publish, or preparation of the manuscript.
**Competing Interests**<br><br>Use the instructions below to enter a competing interest statement for this submission. On behalf of all authors, disclose any competing interests that could be perceived to bias this work—acknowledging all financial support and any other relevant financial or non-financial competing interests.	The authors have declared that no competing interests exist.

This statement is required for submission and **will appear in the published article** if the submission is accepted. Please make sure it is accurate and that any funding sources listed in your Funding Information later in the submission form are also declared in your Financial Disclosure statement.<br><br>View published research articles from *PLOS ONE* for specific examples.<br><br>**NO authors have competing interests**<br><br>Enter: *The authors have declared that no competing interests exist.*<br><br>**Authors with competing interests**<br><br>Enter competing interest details beginning with this statement:<br><br>*I have read the journal's policy and the authors of this manuscript have the following competing interests: [insert competing interests here]*<br><br>* typeset	
**Ethics Statement**<br><br>Enter an ethics statement for this submission. This statement is required if the study involved:<br><br>• Human participants<br>• Human specimens or tissue<br>• Vertebrate animals or cephalopods<br>• Vertebrate embryos or tissues<br>• Field research<br><br>Write "N/A" if the submission does not require an ethics statement.<br><br>General guidance is provided below. Consult the submission guidelines for detailed instructions. **Make sure that all information entered here is included in the**	All necessary permits were obtained for the described study, which complied with all relevant regulations from the Belize Fisheries Department.

**Methods section of the manuscript.**<br><br>**Format for specific study types**<br><br>**Human Subject Research (involving human participants and/or tissue)**<br>• Give the name of the institutional review board or ethics committee that approved the study<br>• Include the approval number and/or a statement indicating approval of this research<br>• Indicate the form of consent obtained (written/oral) or the reason that consent was not obtained (e.g. the data were analyzed anonymously)<br><br>**Animal Research (involving vertebrate animals, embryos or tissues)**<br>• Provide the name of the Institutional Animal Care and Use Committee (IACUC) or other relevant ethics board that reviewed the study protocol, and indicate whether they approved this research or granted a formal waiver of ethical approval<br>• Include an approval number if one was obtained<br>• If the study involved *non-human primates*, add *additional details* about animal welfare and steps taken to ameliorate suffering<br>• If anesthesia, euthanasia, or any kind of animal sacrifice is part of the study, include briefly which substances and/or methods were applied<br><br>**Field Research**<br><br>Include the following details if this study involves the collection of plant, animal, or other materials from a natural setting:<br>• Field permit number<br>• Name of the institution or relevant body that granted permission	
**Data Availability**<br><br>Authors are required to make all data underlying the findings described fully available, without restriction, and from the time of publication. PLOS allows rare exceptions to address legal and ethical concerns. See the PLOS Data Policy and FAQ for detailed information.	Yes - all data are fully available without restriction

Powered by Editorial Manager® and ProduXion Manager® from Aries Systems Corporation

A Data Availability Statement describing where the data can be found is required at submission. Your answers to this question constitute the Data Availability Statement and **will be published in the article**, if accepted.<br><br>**Important:** Stating 'data available on request from the author' is not sufficient. If your data are only available upon request, select 'No' for the first question and explain your exceptional situation in the text box.<br><br>Do the authors confirm that all data underlying the findings described in their manuscript are fully available without restriction?	
**Describe where the data may be found in full sentences. If you are copying our sample text, replace any instances of XXX with the appropriate details.**<br><br>• If the data are **held or will be held in a public repository**, include URLs, accession numbers or DOIs. If this information will only be available after acceptance, indicate this by ticking the box below. For example: *All XXX files are available from the XXX database (accession number(s) XXX, XXX.*).<br>• If the data are all contained **within the manuscript and/or Supporting Information files**, enter the following: *All relevant data are within the manuscript and its Supporting Information files.*<br>• If neither of these applies but you are able to provide **details of access elsewhere**, with or without limitations, please do so. For example:<br><br>*Data cannot be shared publicly because of [XXX]. Data are available from the XXX Institutional Data Access / Ethics Committee (contact via XXX) for researchers who meet the criteria for access to confidential data.*<br><br>*The data underlying the results*	All relevant data are within the manuscript and its Supporting Information files.

presented in the study are available from (include the name of the third party and contact information or URL).<br>• This text is appropriate if the data are owned by a third party and authors do not have permission to share the data.<br><br>* typeset	
Additional data availability information:	

Manuscript
Click here to access/download;Manuscript;Greer et al Coral Gardens PLOS manuscript.docx

Coral Gardens Reef, Belize: An *Acropora* spp. refugium under threat in a warming world

Lisa Greer[1*], H. Allen Curran[2], Karl Wirth[3], Robert Humston[4], Ginny Johnson[1], Lauren McManus[1,5], Candice Stefanic[1,6], Tara Clark[7,8], Halard Lescinsky[9], Kirah Forman-Castillo[10]

[1]Department of Earth and Environmental Geoscience, Washington and Lee University, Lexington, VA, USA 24450

[2]Department of Geosciences, Smith College, Northampton, MA, USA 01063

[3]Department of Geology, Macalester College, St. Paul, MN, USA 55105

[4]Department of Biology, Washington and Lee University, Lexington, VA, USA 24450

[5]Kogod School of Business, American University, Washington, DC, USA 20016

[6]Department of Anatomical Sciences, Stony Brook University, Stony Brook, NY, USA 11794

[7]School of Earth, Atmospheric, and Life Sciences, University of Wollongong, NSW, Australia 2522

[8]Radiogenic Isotope Facility, School of Earth and Environmental Sciences, The University of Queensland, QLD, Australia 4072

[9]Department of Biology and Earth Science, Otterbein University, Westerville, OH, USA 43081

[10]Hol Chan Marine Reserve, San Pedro, Belize

*Corresponding author: greerl@wlu.edu (LG)

Keywords: *Acropora*, Belize, coral, reef, refugium, Caribbean, sea surface temperature, climate change, hurricanes

Abstract

Live coral cover has declined precipitously on Caribbean reefs in recent years. *Acropora cervicornis* coral has been particularly decimated, and few Western Atlantic *Acropora* spp. refugia remain. Coral Gardens, Belize, was identified in 2020 as a long-term refugium for this species. This study assessed live *A. cervicornis* coral abundance over time at Coral Gardens to monitor the stability of *A. cervicornis* corals, and to explore potential threats to this important refugium. Live coral cover was documented annually from 2012-2019 along five permanent transects. *In situ* sea-surface temperature data were collected at Coral Gardens throughout the study period and data on bathymetry, sediment, substrate, herbivore abundance, and macroalgal abundance were collected in 2014 and 2019 to assess potential threats to Coral Gardens. Live coral cover declined at all five transect sites over the study period. The greatest loss of live coral occurred between 2016 and 2017, coincident with the earliest and highest warm-season temperatures recorded at the study site and the passage of a hurricane in 2016. Yet Degree Heating Weeks (DHW), a measure of threshold temperature for coral bleaching, did not accumulate during the study period, and structural storm damage was not observed at Coral Gardens. Uranium-thorium (^{230}Th) dating of 26 dead *in situ* fragments of *A. cervicornis* collected in 2015 from Coral Gardens further revealed no correlation between coral mortality and tropical storms and hurricanes in the recent past. Our data suggest that several other common drivers for coral decline (i.e. herbivory, predation, sedimentation, pH) may likely be ruled out for Coral Gardens. At the end of the study period, Coral Gardens satisfied most criteria for refugium status. However, the early onset, higher mean, and longer duration of above-average temperatures as well as intermittent temperature anomalies likely play a critical role in the stability of this refugium, even when DHW

do not accumulate. We suggest that sub-lethal temperature stress in 2016 may have increased coral tissue vulnerability at Coral Gardens to a passing hurricane, threatening the status of this unique refugium.

Introduction and background

Acropora corals (*A. cervicornis* and *A. palmata*) have historically provided the structural support for many Caribbean Pleistocene and Holocene reefs, creating complex architectural framework and substrate for shallow-water reef dwellers (Mesolella, 1967; Jackson, 1992). The more fragile of the two acroporids, *A. cervicornis* (staghorn coral), is generally found living in shallow waters (5-20 m) on fore- and patch reefs (NMFS, 2015) and has a growth rate of between three to over 11 cm per year (Shinn, 1966; Gladfelter et al. 1978). The fossil record shows that acroporid corals are a well-documented component of pre-modern reefs in Belize (Aronson and Precht, 1997; Gischler and Hudson, 2004; Gischler, 2007), and studies suggest that acroporids have persisted as an important component of Caribbean reefs in the face of climate and environmental changes in the geologic past (Greenstein et al., 1998; Wapnick et al., 2004; Pandolfi and Jackson, 2006; Greer et al., 2009; Precht et al., 2014). Nonetheless, some estimates suggest that acroporids have declined by up to 98% since the 1980s, or a few decades prior (Precht et al., 2002; Cramer et al., 2020). Coral Gardens, Belize has been identified as a refugia for these endangered species (Greer et al., 2020).

While other extant acroporid-dominant coral communities have been reported in the last decade or so (Vargas-Angel et al., 2003; Keck et al., 2005; D'Antonio et al., 2016), few of these are reported to be extensive and/or true reef-building structures (Purkis et al., 2006; Lirman et

al., 2010a). The degree to which any of these sites may also qualify as an *Acropora* spp. refugium remains in question, making Coral Gardens a valuable site for monitoring this species. Coral reef refugia were first recognized by Glynn (1996) as habitats protected from rapid temperature rise and coral bleaching, but have been defined in numerous ways using differing criteria (Riegl et al., 2009; Keppel et al., 2012; Kavousi and Keppel, 2017). In general, a refugium is a place where a species may retreat to, persist in, and expand from (Keppel et al., 2012). Low exposure to stressors can be key to refugium maintenance. Habitats that provide a buffer for organisms from climate stressors on timescales shorter than decades, healthy reefs that are located in remote locations distant from anthropogenic impacts, and reefs primarily composed of species that are tolerant to extreme climatic conditions should not be counted as true refugia according to Kavousi and Keppel (2017). Longevity is key to refugium designation but assessing how long a reef has existed in a given area can be particularly challenging unless it has been monitored for decades.

In 2017, the International Union for Conservation of Nature (IUCN) listed the Belize Barrier Reef system as an area of "significant concern". This report cited habitat shifts, sea-level rise, temperature extremes, and potential increase in hurricanes as a "very high threat", and overfishing, coastal development, tourism, sedimentation, and ocean acidification as a "high threat" to the future integrity of Belize reefs (IUCN, 2017). Contrary to these concerns, living coral communities within Coral Gardens are comprised of up to 75,000 m^2 of extensive framework-building acroporid thickets (Busch et al. 2016). Irwin et al. (2017) used a novel genetic aging technique (Devlin-Durante et al. 2016) to show that some genets of *A. cervicornis*, *A. palmata*, and even the F1 hybrid *A. prolifera* are long-lived (up to hundreds of years in age) at Coral

Gardens. Greer et al. (2020) assessed reef longevity using more traditional radiometric dating techniques to show that *A. cervicornis* presence at Coral Gardens at least pre-dated and persisted through the widespread Caribbean acroporid die-offs of the 1980s and earlier. This site therefore provides a unique opportunity to assess and monitor the continued viability of a long-standing endangered coral species refugium.

Numerous local and global factors are known to threaten coral reefs (Hoegh-Guldberg, 1999; Carilli et al., 2009; Hughes et al., 2017). Coral reefs are not uniform in the pressures they face or how they respond to these threats (Guest et al., 2018). Rising sea-surface temperatures (SST), particularly in years where El Niño-related warming compounds the effects of global warming, increasingly stress corals and can induce coral bleaching (Glynn and D'Croz, 1990; Eakin et al. 2010). Excessive macroalgae growth due to overfishing, eutrophication, or loss of key reef herbivores can inhibit coral recruitment and the ability of reefs to recover from disturbance (Pandolfi et al., 2005; Birrell et al. 2008). Tropical storms and hurricanes have always impacted reefs, but these events may be increasing in strength or frequency as a result of changing climate (Bender et al., 2010). Land-based pollution, sedimentation, and contaminants resulting from coastal development and land use can also negatively impact reefs (Fabricius, 2005). Unmitigated or poorly managed marine tourism may result in harm to reefs through boat groundings, anchor damage, or snorkeler/diver damage (Barker and Roberts, 2004), and even when Marine Protected Areas are designated, location, size, lack of connectivity with other safe spaces, or inadequate enforcement can exacerbate problems for coral reef communities at these sites (Roberts, 1997). Excessive bioerosion, predation, or loss of carbonate accretion can have detrimental impacts to the structural integrity of reefs (Fang et al., 2013). Finally, one of the most

damaging effects on acroporid coral abundance throughout the wider Caribbean is White Band Disease (WBD), which has been devasting *Acropora* spp. corals for at least the last few decades (Gladfelter, 1982; Aronson and Precht, 2001).

This study aims to evaluate the potential threats to the Coral Gardens refugium based on available data. In this study, we surveyed live coral abundance at an existing *A. cervicornis* refugium annually from 2012 through summer 2019. Our data show that while this refuge still persists, it remains under threat. High resolution calculations of live coral cover, in situ SST data, ^{230}Th dating of dead coral fragments, macroalgae and herbivore abundance, sediment analysis, and observational data were used to evaluate potential drivers of declining coral cover at Coral Gardens. Using a heuristic process of elimination approach, we suggest that while most 'threat variables' did not change at Coral Gardens during the study, sub-lethal thermal stress (below bleaching thresholds) may have increased the vulnerability of *A. cervicornis* to storms.

Study area

Coral Gardens Reef (17°50'00.36"N, 87°59'32.45"W) is located inshore of the Mesoamerican Barrier Reef between the islands of Ambergris Caye and Caye Caulker. It sits between two marine protected areas, the Hol Chan and the Caye Caulker Marine Reserves. Coral Gardens is a system of shallow water (<7 m) patch reefs dominated by ~7.5 hectares of *A. cervicornis* and *A. palmata*, and it contains one of the largest recorded acroporid populations in the Caribbean (Fig 1; Busch et al., 2016). All three Caribbean *Acropora* species (*A. cervicornis*, *A. palmata*, and *A. prolifera*) are present at Coral Gardens in substantive thickets that are interspersed between mixed massive and finger corals (e.g., *Millepora*, *Porites*, *Orbicella*, and

Agaricia spp.) and sandy areas. The branching nature of acroporids provides habitat to a number of herbivore species, notably *Echinometra viridis* urchins and several species of damselfish (e.g., *Stegastes adustus, Stegastes planifrons,* and *Microspathodon chrysurus*). The mostly monospecific thickets of branching acroporids vary in size and can reach 35 m in diameter. The study site has periodically experienced the passage of tropical storms and hurricanes, but it is not located in the outflow path of any major rivers. Rainfall and SST variability at Coral Gardens are presumed to be roughly similar to other sites in Belize where acroporids are less abundant.

Fig 1. Location map for Coral Gardens showing the location of transects used in the study. Coordinates are UTM northings and eastings in Zone 16.

Materials and methods

Site selection

Coral Gardens was first surveyed in 2011 when reconnaissance studies were conducted across the wider perimeter of the site in search of monitoring locations. The first transect site (T1) was established and marked with rebar stakes placed in hardground or non-living coral framework (Fig 1). To best represent potential variability across the reef and collect data from a wider area, four additional transects (T2, T3, T4, and T5) were established in 2012. All five transect locations were documented with high-resolution GPS measurements taken using a Geoexplorer 6000 series Trimble GPS unit. Transects vary in length from 12-36 m in length. To explore patterns in live coral over time, we treated each transect as an independent replicate

sample from a distinct coral patch. Live coral cover in each transect was calculated as the average of quadrat samples within the transect (see below). Mean annual coral cover at Coral Gardens was calculated as the average across transects, with each transect weighted equally.

Field documentation

Acropora cervicornis was surveyed at all five transect locations using the same protocol from 2012-2019 during each summer (excluding 2015) for 7 years. Transects 1 and 5 were also surveyed in October 2016. To survey coral abundance quantitatively, a measuring tape was placed between transect markers at the beginning and end of each transect. Using a 1m^2 scaled quadrat constructed from PVC pipes multiple photographs were taken from overhead (map view) at 1 m intervals by divers using at least two cameras to optimize image quality options for post-processing and in case of camera malfunction (Fig 2). This process was replicated at each meter along each transect. A variety of underwater cameras were used over the years, including Panasonic Lumix, Olympus Tough, FujiFilm XP, and Nikon Coolpix. In 2013, the perimeters of *A. cervicornis* patches were mapped using the handheld Geoexplorer 6000 Trimble GPS unit by having a small boat closely follow a swimmer (within ~3 m) who followed the contour of all areas of the reef (Fig 1).

Fig 2. Live coral assessment methods. (A) Diver placing a m^2 quadrat above reef acroporids: (B) Raw image taken from above the quadrat; (C) Image rectified (cropped and stretched) using a MATLAB script; (D) Same image with live coral manually traced

using Adobe Photoshop; (E) Same area (live coral now in black) that was used to quantify the area of live coral tissue per m^2 using a MATLAB script.

Image processing

Multiple photographic images were obtained for each individual 1 m^2 quadrat and assessed for angle, focus, lighting, and spatial completeness (showing all four corners of the quadrat scale). Images that best enabled discrimination between coral species, live coral, encrusting algae, macroalgae, and other hard rubble substrates was selected for processing. Individual quadrat images were cropped and rectified using MATLAB software, with scripts developed by Stough et al. (2012) (Fig 2). The calibrated photographs were manually segmented (traced) by individual evaluators using Adobe Illustrator (Fig 2). Evaluators outlined all live *A. cervicornis* tissue by using the Pen Tool to click along the contours of living branches and enclose all visible live coral within polygons that were then filled in solid black. A white rectangle in a separate, posterior layer was used to create a completely black and white image which was exported as a Portable Network Graphic (PNG) image file (Fig 2). A MATLAB script was used to calculate percent live coral (i.e., pixel percentage of black in the image). Every completed image was assessed and edited by a second evaluator for completeness and accuracy to ensure consistency between evaluators. Inter-evaluator variability was assessed during training until a standard deviation of less than 0.01% per image was achieved. Because photographs only represent a two-dimensional map view of three-dimensional branching thickets, live coral quantified by this method is a conservative measure of actual live *A. cervicornis* tissue biomass but well represents 2D coverage on the reef (Nylander et al., 2020).

Temperature

Temperature data were collected at T1 and T5 in Coral Gardens at 1 to 60- minute intervals from 5/9/12 to 7/2/19 using HOBO Pendant Temperature and Temperature/Light Data Loggers (UA-001 and UA-002) manufactured by the Onset Computer Corporation. Multiple loggers were deployed each year and compared for cross calibration and/or drift. Loggers were deployed 2-3 m below mean sea level and attached with zip ties to non-living reef substrate adjacent to *A. cervicornis* thickets. A single time series was constructed from validated loggers. SST data were averaged to daily, monthly, and annual means for data analysis. Maximum monthly mean (MMM) temperatures were evaluated for each year to calculate Degree Heating Weeks (DHW), a cumulative measure over a moving 3-month window as defined by weeks above the MMM (for example, 2 DHW may equal 2 weeks above MMM or 2°C over MMM for 1 week). Global SST and land-based temperature data used in this study were accessed from the National Oceanic and Atmospheric Administration (NOAA) National Centers for Environmental Information "Climate at a Glance" Global Time Series, published June 2020 (retrieved on June 18, 2020 from https://www.ncdc.noaa.gov/cag/).

pH

In 2019, sixty-four temperature and pH measurements were collected with two PTTestr 35 instruments at 1 m depth using a LaMotte water sampler. A 3-point calibration was performed daily, and samples were collected over a five-day period (June 29-July 3, 2019) at four locations off the southern end of Ambergris Caye. Samples were collected at least 50 m from shore to avoid

the localized effects of decomposing *Sargassum* sp. near shore that in recent years has become an annual spring and summer event. The mean pH of 8.1 (n=64) was obtained, with a range of 7.7-8.3. Only eight measurements fell below a pH of 8.0.

Sediment, substrate and macroalgal abundance

A point count method was used to estimate the composition of substrate along the center of each transect at 0.5 m intervals from all quadrat photographs in 2014 and at the same sample interval in the field in 2019. Live coral, bare rock, sediment, and macroalgae were noted, with the number of points per transect serving as a low-resolution proxy for percent substrate. Nineteen sediment samples were collected from the upper 1-2 cm of seafloor around the edges of coral thickets. Samples were dried, sieved, weighed, and examined for grain size analysis and composition. A thin steel measuring probe was used to measure canopy height, sediment thickness, and depth to hardground along transects 1 and 5 in 2014.

Herbivory

Urchin counts were performed in the field for every transect quadrat in both 2014 and 2019. All urchins were counted twice per m^2 by two evaluators and averaged. Over 99% of the urchins counted were identified as *Echinometra* spp. Visual surveys of damselfish (*Stegastes adustus, Stegastes planifrons,* and *Microspathodon chrysurus*) were conducted in 2014 and 2019. Fish were observed for every m^2 of the transects for ~1 minute by two evaluators hovering at least 1 m above the substrate. Fish were recorded if they occupied the area for over 3 seconds and individual fish species were not differentiated.

Results

Live coral abundance

In total, over 1000 individual quadrat images were collected between 2012-2019 at the five transect sites. Over the course of the study, live coral coverage declined at all transect locations (Table 1, Fig. 3A). Between 2012 and 2019, percent live coral cover (averaged across the five transects) declined by 16.2% per m^2. Significance of the temporal decline was confirmed by repeated-measures ANOVA ($P<0.01$). The site with the most abundant live *A. cervicornis* (T5) suffered a loss of 32.6% and serves as an outlier among the replicate transects (Figure 3A), with the greatest loss between 2016 and 2017. Pairwise comparisons of sequential annual means indicate that only the difference in live coral cover between 2016 (mean = 22.8, SD = 9.6) and 2017 (mean = 14.4, SD = 6.4) is significant (pairwise t-test with sequential Bonferroni adjustment; $p = 0.05$). Only two sites (T1 and T5) were surveyed in both summer and fall of 2016. Both sites showed a significant decline in live coral between June and October of that year (Figs 3C and Table 1), with a mean loss of live coral tissue of 7.1% at T1 and 16.9% at T5 and an overall decline of 11.9% living coral for the two sites. Live coral tissue declined at all but one comparable quadrat along T1 and T5 over the four months.

Year	n	T1	T2	T3	T4	T5	Mean
2012	133	19.61	29.69	24.21	23.73	58.05	31.06
2013	129	19.31	25.98	23.05	32.68	56.16	31.43
2014	130	14.54	28.28	21.05	19.39	51.67	26.99
2015	0						
2016	131	13.70	28.78	13.39	22.52	35.55	22.79
2016 Oct	67	6.63				18.65	12.64

2017	133	8.70	15.81	6.97	22.27	18.09	14.37
2018	140	9.85	16.48	4.63	16.45	19.52	13.39
2019	141	12.31	14.92	5.33	16.07	25.48	14.82

Table 1. Number of m^2 quadrats and percent mean live coral abundance per year from 2012-2019 at Coral Gardens. Transect locations are indicated with T1-T5, and n is the number of m^2 photographed quadrats analyzed. The mean is the percentage mean of five equally weighted transect populations.

Fig 3. Summary of mean live coral per m^2 at each of the Coral Gardens transect sites from 2012-2019. (A) Mean percent live coral per year per transect with 1-sigma errors; (B) Boxplot of live coral percentage averaged across all five transects shown by year (representing treatment as 5 replicates). Shaded rectangles indicate the interquartile range, the whiskers indicate the minimum and maximum values, the thick horizontal lines are at the median values, and filled circles are statistical outliers; (C) Boxplot of live coral at Transects 1 and 5 in June and October of 2016. Symbols as in 3B. A and B do not include October 2016 data.

Temperature

The hottest year on modern record prior to the end of this study occurred in 2016, an El Niño year, with global temperatures 1.0°C above the 20th century average for land and ocean (LO) temperatures combined and 0.79°C above average ocean temperature (SST; NOAA, 2020). That year was also the hottest year on average at Coral Gardens (Table 2, and Figures 4 and 5).

The year 2016 averaged 29.0°C, 0.2°C above 2014, 2017, and 2015, 0.4°C warmer than 2013, and almost half a degree warmer than 2018 (Table 2). In most years, the warmest months were between July and October, and the lowest SSTs were recorded between December and March. Mean monthly SSTs heated sooner in 2016 than in any other year except 2014, surpassing 28°C in April 2016. Coral Gardens experienced the hottest May, January, June, July, and October of the study period in 2016, and the core temperature (inter-quartile) range was elevated for 2016 as well (Fig 5). There were notable cold spells in March 2013 (dipping to 25.4°C on March 2) and late January/early February 2016 (with a low of 25.6°C on February 6) and heat anomalies in 2015, 2016, and 2017 (above 31°C) (Fig 4). Lowest monthly temperatures varied in timing from March in 2013 to January in 2018 (Fig 4). The lowest mean annual temperatures occurred in 2018, a year that also experienced the lowest maximum temperatures in the study period. One metric for heat stress (or bleaching threshold) for corals is defined as an event (i.e., day) that is 1°C warmer than the maximum monthly mean (MMM) temperature for that year (Glynn and D'Croz, 1990). At Coral Gardens, SSTs exceeded that threshold on August 30, 2015 and came within 0.01°C of reaching that level on October 7, 2016. These conditions were not met in any other years, therefore DHW did not accumulate at this site during the study period.

Year	Mean Annual Temp °C	Max Recorded Temp °C	Min Recorded Temp °C	Min Temp °C J-O	Annual Temp Range °C
2013	28.64	30.84	25.40	28.56	5.44
2014	28.83	31.00	26.41	27.45	4.59
2015	28.81	31.42	26.27	27.97	5.15
2016	29.04	31.24	25.59	28.97	5.66
2017	28.82	31.46	26.64	28.46	4.82
2018	28.58	30.62	25.72	28.59	4.90

Table 2. Summary of annual (calendar year) temperature data at Coral Gardens. J-O indicates June through October (warm season).

Fig 4. Mean daily and monthly temperature data at Coral Gardens from 2012-2019.

Fig 5. Summary of temperature data in form of a violin plot of daily average temperature by year with inset box plots. Outer lines depict distribution of observations within the sample year. Box plots depict the standard quartiles and data range.

Sediment analysis, substrate, and bathymetry

The seafloor sediment was composed entirely of carbonate and organic material ranging from *in situ* micrite to coarse sand-sized skeletal and calcareous algae grains. Sediment surrounding coral thickets was coarse, with fines (<64 μm) not exceeding 2% of the cumulative sediment sample weight at any location. Sediment thickness above hardground averaged roughly only 2-3 cm in depth proximal to transect locations and rarely exceeded 8 cm thickness in small pockets around the perimeter of the *A. cervicornis* patches. Sediment thickness beneath coral canopy was generally on the order of a cm or less. Coral canopy height varied from ~3.9-1.7 m below mean sea level at transects 1 and 5 in 2014, and depth to hardground varied from ~4.3-2.8 m below sea level, resulting in an approximate average coral canopy height of 0.7 m above substrate at T1 and 1.1 m above substrate at T5. A total of 284 points were evaluated for substrate composition along the transects in 2014 and 2019.

Macroalgae and herbivory

Echinometra spp. urchins were abundant at Coral Gardens. The mean number of urchins per m^2 was 20.4 (SD = 14) in 2014 and 24.1 (SD = 13.5) in 2019. The average damselfish abundance was 2.0 (SD = 1.3) per m^2 in 2014 and 2.3 (SD = 1.2) in 2019. Statistical comparisons indicate these are not significant differences. Likewise, there was no significant change in macroalgae abundance between 2014 (mean = 5.4%, SD = 2%) and 2019 (mean = 4.5%, SD = 3.7%) ($p = 0.66$) although point count data indicate a significant decline in *A. cervicornis* between these years (pairwise t-test; $P = 0.03$).

Discussion

Our data show that live *Acropora cervicornis* cover declined significantly at Coral Gardens during the study period. While live coral abundance decreased overall at every transect, healthy *A. cervicornis* colonies remained at all sites. The greatest loss in total live coral tissue in the study period occurred between June 2016 and June 2017. This loss coincides with a year when temperatures warmed earlier, stayed higher, and lasted longer than in other years of the study (Table 2, Figures 4 and 5), as well as the passage of Hurricane Earl in August 2016. However, temperatures did not exceed Maximum Monthly Mean (MMM) or reach the Degree Heating Weeks (DHW) bleaching threshold, and significant physical damage from the storm was not observed. Below we discuss temperature, storms, and additional potential factors contributing to both decline and persistence of *A. cervicornis* at Coral Gardens and, by process of elimination, attempt to discern the most likely factors for changing coral abundance in this unique refugium.

Candidates for *Acropora cervicornis* Decline at Coral Gardens

Herbivory, Macroalgal Abundance, and Predation: The detrimental effects of macroalgal occupation of potential recruitment and growth locations for corals has been well recognized (Pandolfi et al., 2005; Birrell et al., 2008). Urchins and damselfish are important herbivores that feed on the algae that covers reef framework, and serve as a critical control on macroalgal abundance (NMFS, 2015; Precht et al., 2010). There is no evidence of a significant change in macroalgal abundance, urchin density, or damselfish density at Coral Gardens during the study period. Although no nutrient data was collected, the reef site is not adjacent to large tracts of fertilized farmland, point-source pollution, or riverine influx. The IUCN Report (2017) lists water pollution as a low threat to the Belize Barrier Reef, and the low percentage of macroalgae substrate and lack of an increase in algae over the study period indirectly support the conclusion that eutrophication was unlikely to be a leading factor in coral decline over the study period.

Excess predation and bioerosion are also potential problems for acroporid reefs. Urchins and damselfish may contribute to bioerosion and grazing of live coral tissue, but they also provide superior habitat for coral settlement by providing clear space for coral recruits so the benefit to corals is usually thought to outweigh cost in terms of space provision (Brown-Saracino et al., 2007; O'Leary et al., 2013). Even if bioerosion was prevalent at Coral Gardens, the web-like canopy for *A. cervicornis* 'props up' coral branches that have become detached at the bottom, and no canopy collapse was observed during the study. Predators known to cause tissue damage to acroporid corals include fireworms (*Hermodice carunculata*) and prosobranch gastropods (e.g., *Coralliophila abbreviata;* Ott and Lewis, 1972), but fireworms and prosobranch gastropod

sightings have been rare to absent at the transect locations (Greer, personal observation) during the study. We conclude that there is no evidence to support predation as a driving factor for coral decline at Coral Gardens.

Sea Level rise, Ocean acidification, and White Band Disease: The decline of coral cover at Coral Gardens cannot be attributed to current rates of sea level rise. *A. cervicornis* is one of the fastest growing Caribbean corals with linear extension rates of 3-11 cm per year that well outpace current rates of sea level rise (Shinn, 1966; Gladfelter et al. 1978). Although ocean acidification is increasingly suggested to provide future challenges for reefs, as well as for certain reefs at present (van der Zande et al., 2020), there is not clear evidence to suggest that acidification already poses a threat to live coral tissue or is currently a threat to all reefs (McCulloch et al, 2012). We do not have reliable long-term pH data for Coral Gardens, but average measurements of 8.1 in June 2019 mirror current normal marine values.

White Band Disease (WBD) has been devastating to acroporids across the Caribbean (Aronson and Precht, 2001). While we cannot accurately distinguish WBD from bleaching of acroporids without histopathology, visual observation of white *A. cervicornis* (i.e., clear tissue revealing white skeleton beneath) at Coral Gardens was minimal in all years of the study period, with few potential WBD cases observed on, or in close proximity to, the actual transects. This does not mean that bleaching or WBD were wholly absent, but evidence of either was only very rarely observed during data collection periods.

Storms: No two tropical storm events are exactly alike and clearly the impacts to reefs may vary in nature and scope. The direction of impact, distance from storm epicenter, intensity, duration, and storm surge all govern impact. This means that the 'category' of the storm is not

the only measure of influence to a reef site. While not perfectly correlative, major storm and hurricane events can damage coral reefs in several ways. Excessive inland rain can cause freshwater flooding (i.e., salinity stress), the influx of terrestrial organic matter and clouding of water (i.e., low light), and runoff of contaminants like fertilizers from farmland to reef (i.e., nutrient influx). Coral Gardens is not located near significant riverine or land-based runoff sources and is therefore unlikely to be impacted by runoff. The physical impacts of wave damage can result in reef flattening, especially of the shallowest coral stands that reach closest to the water's surface. Even when canopy collapse does not occur during a storm, loose branches may experience some abrasion if mobilized. No canopy collapse or major structural damage was observed at Coral Gardens during the study period.

To explore whether storms have resulted in recognizable mortality events prior to the study period at Coral Gardens, we compared ^{230}Th dates from a previous analysis of dead corals excavated from Coral Gardens (Greer et al., 2020; Fig 6) with known storm events that passed in close proximity from 1980-2019 (NOAA, 2015; Consejo, 2020). All necessary permits (CITES and Belize Fisheries Management Unit) were obtained for the collection of all samples in this study, which complied with all relevant regulations and ages were obtained using methods from Clark et al. (2014a,b). The ^{230}Th dates from dead coral samples revealed peaks in ages at around ~1994 and ~2009. No tropical storms or hurricanes passed near Coral Gardens from 1981-1998, or in 2009. Tropical storms and hurricanes with potential for damage did pass in close proximity to the site in 1980, 1998, 2000, 2001, 2007, 2008, 2010, 2012, 2016, and 2017. However, there appears to be no clear correlation between known storms events and coral mortality dates.

Fig 6. *Acropora cervicornis* ^{230}Th mortality events and known storms. (A) *Acropora cervicornis* ^{230}Th ages (±1s error bars) determined from 26 coral fragments collected from the substrate of transect T5 and excavated proximal to the transect. In this plot, significant mortality events would be indicated by several samples with the same or similar ages (e.g., forming a horizontal array); (B) Histogram and relative probability curve of *Acropora cervicornis* ^{230}Th mortality events. The relative probability curve (black) shows peaks in coral mortality centered at 1986, 1994, and 2009. Diamonds indicate number of known tropical storms and hurricanes in proximity of Coral Gardens in a given year.

Strong storms passed near Coral Gardens with no coincident mortality dates, yet other dates suggest mortality coincident with milder storms or no storms at all. No tropical storms or hurricanes impacted the field area in Belize between 1981 and 1998 which bracketed 14 of the 26 ^{230}Th ages (Fig. 6). Hurricane Mitch, one of the deadliest Atlantic hurricanes on record, passed south of Belize as a Category 5 storm in 1998. Extensive damage to reefs was reported at Glovers Atoll to the south, with up to 90% mortality of *Acropora palmata* (Mumby, 1999) at the site. Personal storm accounts reported significant hurricane damage to infrastructure and the coastline of Ambergris Caye with high waves at the reef crest. Furthermore, Mitch was coincident with a significant bleaching event (Mumby, 1999). Yet no ^{230}Th ages coincide with this year. In 2000, Hurricane Keith arrived just offshore of Ambergris Caye as a Category 4 hurricane with peak winds of 225 km/h on October 1, causing considerable damage on land (Bevan, 2001). No coral ^{230}Th ages coincide with this event and only one does so in the next year. In 2007, Hurricane Dean

landed in Mexico about 65 km north of Belize as a Category 5 storm, and major damage was reported in Corozal (Cohrs, 2007), but the San Pedro Sun reported that "Early estimates and initial hotel reports indicate very minimal damage to areas such as San Pedro and Caye Caulker" (https://ambergriscaye.com/sanpedrosun/old/07-331.html). Tropical Storm Arthur arrived in northern Belize from the Pacific in late May of 2008, reportedly causing heavy surf near Ambergris Caye (Ysaquirre, 2008), but no major damage. No coral mortality dates match 2007, but 3 of the T5 coral samples died in 2008, and two the year after when no storms passed Coral Gardens.

In 2016, Tropical Storm Earl strengthened to a Category 1 hurricane with 147 km/h winds before making landfall about 10 km south of Belize City on August 4 (Bevan, 2016). This storm caused damage to docks and coastal infrastructure on Ambergris Caye with heavy rainfall and maximum storm surge estimated to be between 1.2-1.8 m (Bevan, 2016; Stewart, 2017). Yet the coral canopy remained intact at Coral Gardens and significant structural damage (reef flattening, denuded patches along the transects) was not apparent. Live coral declined across all areas of the reef with the decrease in abundance distributed across all areas of the surveyed sites (cover declined in all but one individual m^2 in T1 and T5 from June to November) in 2016. The only other storm to pass near Coral Gardens was Tropical Storm Franklin, which passed just north of Belize on August 8, 2017 (Bevan, 2018). Again, no structural damage was noted at the site. Although Hurricane Earl cannot be ruled out as a contributing force for coral mortality between the June and October surveys in 2016, precedent suggests that *A. cervicornis* at Coral Gardens persisted despite close proximity to several tropical storms and hurricanes in the recent past.

Sedimentation: Excess sedimentation or mobilization of sediment can pose a significant threat to coral reefs (Risk, 2014). Little loose sediment is present at Coral Gardens and most of the sediment is not fine enough to remain in suspension for long under normal conditions. The sediment surface sits 3-4 m below the surface at Coral Gardens, and the coral canopy rises between 0.7-1.1 m above substrate on average, so it is unlikely that sedimentation stress is a driving threat to the corals under ordinary conditions. However, sediment can be resuspended at great depths during storm wave circumstances (Bianucci et al, 2018), potentially leading to abrasion of coral tissue and an energetic cost of sediment removal for the coral animal.

Additional threats to reefs: Recreational activity can certainly be detrimental to the health of a reef and has been cited as a contributing factor to the threatened status of *Acropora* spp. reefs (NMFS, 2015). Anchor and chain damage, vessel groundings, careless snorkelers or divers, lost fishing gear, and interference in herbivore population densities can create physical as well as ecosystem damage unfavorable to coral growth, especially for branching *Acropora* spp. in shallow habitats (Donohoe et al., 2001; NMFS, 2015). Tourist visitation metrics have not been assessed at Coral Gardens, but there were no reported groundings in the vicinity during the study period and we did not observe any obvious structural damage at the transect locations. Although Coral Gardens is visited by commercial and recreational fishers and snorkelers, the area is situated within a few kilometers of more established recreation sites, including the Hol Chan Marine Reserve, Shark and Ray Alley, and the Caye Caulker Marine Reserve, all of which draw heavier tourist attention than Coral Gardens.

Temperature: It does not appear that the factors discussed above are likely to be significant drivers of coral decline at Coral Gardens, with the possible exception being sediment-

derived abrasion of live coral tissue during Hurricane Earl. Using a heuristic process of elimination approach, this leads to temperature as a leading candidate contributing to mortality of *A. cervicornis* at Coral Gardens during the study period. The coincidence of both anomalous temperatures and the passage of Hurricane Earl in 2016 may be especially significant. Both the intensity and the duration of heat anomalies can contribute to coral stress (Glynn and D'Croz, 1990). Global data and data from Coral Gardens show that 2016 particularly exceeds the next-ranked heat years during warmer months and sustained anomalous heating is evident throughout the full calendar year with normally cooler months (i.e., January through May) being particularly warm on average in 2016 (Figures 4 and 5).

Temperature variability within a year and the timescale of variation are also potentially important factors impacting coral health. Variability can be measured as the range in temperature in a given year or season, average standard deviation within a given time frame, or isolated hot or cold events. At Coral Gardens, 2016 was the year with the highest range in daily temperature (5.7^{o}C) in a calendar year; 0.2^{o}C–1.1^{o}C greater than in other years of this study (Table 2), primarily due to substantial cold events (> 1°C colder than the coldest monthly average) in January and February (Fig. 4). The temperatures warmed sooner, were on average much higher, stayed higher, and had a wider range in 2016. Coral Gardens did not accumulate DHW during the study period; however, this does not account for the potential heat stress that accumulates with rising temperatures below that bleaching threshold.

Corals and their algal symbionts are physiologically subject to heat stress prior to the onset of outward physical expression, as evidenced by certain biomarkers for heat stress prior to bleaching (Kenkel et al., 2013; Rosic et al., 2014; Louis et al., 2017). Genetic markers linked to

coral metabolism, heat shock protein response, immune response, and the transport of Ca^{2+} across cell membranes (key to calcification) have been documented (Kenkel et al., 2013; Louis et al., 2017). During thermal stress, photosynthetic dysfunction can damage nucleic acids, oxidize cellular membranes, and denature proteins within the cell (Lesser, 2006; Weis, 2008). Paradis et al. (2019) showed that important physiological changes in the ratio of photosynthesis to respiration (P:R) occur in *A. cervicornis* corals with increasing temperature above the thermal optimum. They measured a significant decline in net photosynthesis at 28°C and 30°C temperatures (in their mesocosm study, optimal P:R was at 25°C), and respiration increased linearly with temperature. They noted that these physiological changes at high temperature were visually undetectable prior to bleaching but resulted in a negative energy balance for the corals. Paradis et al. (2019) also noted that even when heat-stressed corals retain algal symbionts, they may have already entered a state of malnutrition, which could lead to increased vulnerability to additional stressors.

With no visual indication, potential heat stress above optimal might be accumulating during days or weeks, as opposed to accepted “threshold” temperatures (Paradis et al., 2019); however, it is not easy to determine optimal temperature in a field setting because corals in different locations may be acclimatized to different temperatures. We suggest that consecutive days above average temperatures (but below a 1°C threshold) might also induce sustained heat stress. Coral Gardens did not accumulate DHW in any year, but the timing of onset to above-average daily temperatures, duration of above-average temperatures, and the stability of temperatures in warm months varied by year. The highest annual mean SST, highest number of days above that mean, and the longest extending consecutive days 1°C above annual daily

average (27 days above 30 ^{o}C into late October) occurred in 2016. It is certainly possible that partial bleaching did occur in that time. While Belize corals have been known to recover from partial bleaching in the past (Mumby, 1999), perhaps 2016 temperatures prohibited greater recovery.

Paradis et al. (2019) also documented a dramatic compounding impact on the ratio of photosynthesis to respiration (P:R) in *A. cervicornis* corals subject to abrasion. They inflicted corals with wounds from ~1.5-5.0 cm^2 in size and compared the metabolic responses of abraded versus non-abraded corals. Their results showed that photosynthesis decreased dramatically, and significantly more in abraded corals at higher temperatures (28°C and 30°C), with up to 90% less oxygen production, even in non-damaged coral tissue in the wounded specimens. While the authors abraded corals as a proxy for diver damage, we suggest it is possible that storms can also cause abrasion damage at Coral Gardens and elsewhere. The energetic cost of sediment removal from coral tissue may compound this impact. Tropical storm and hurricane sediment mobilization may also cause abrasion even when the structural foundation is maintained. In addition to sediment suspension, we have observed that even when architectural integrity remains sound, loose branches may move and touch each other during high wave activity. Under optimal temperatures corals may be able to heal, but at higher temperatures they might not. We suggest that although storms may not have resulted in recognizable dated mortality events of the past at Coral Gardens, temperature conditions in 2016 could have resulted in an increased vulnerability of corals to the passage of Hurricane Earl.

Resilience, Persistence, and the Refugium concept

Despite the decrease in overall live coral cover from 2012 to 2019, *A. cervicornis* stabilized at Coral Gardens after 2016, and statistical comparisons indicate no significant change in live coral abundance after 2017. The lack of coral decline after 2016-2017 coincided with lower mean annual SST values in 2018 and 2019 (0.2^{o}C and 0.5^{o}C lower than 2016, respectively), fewer months that averaged above 29°C in 2018, and a significantly lower range in SST values in 2017 and 2018 (Table 2) in the study period. In 2019 there were still areas of Coral Gardens with abundant *A. cervicornis* coral cover that exceed reports of abundance in most areas of the Caribbean that we know of (Fig 7). The very lack of the coral stressors discussed here, excluding temperature, and perhaps in combination with a passing storm, may in part explain the apparent resistance of *A. cervicornis* to collapse (even if compromised in 2016) at Coral Gardens.

Fig 7: *A. cervicornis* at Transect 5 in 2019.

Connectivity to adjacent vibrant, healthy, or protected reef areas can be an important factor in reef health (Roberts, 1997). Coral Gardens is situated between two marine protected areas: the 15.4 km^2 Hol Chan Marine Reserve established in 1987 and the 39.1 km^2 Caye Caulker Marine Reserve established in 1998 (http://protectedareas.gov.bz/marine-reserves/). The heavily protected reef portions of these reserves are no-take, no-touch, and they both include ranger surveilance. Visitors are required to be accompanied by a licensed Belize tour guide employed by a licensed tour operator for water entry. The operators and guides at Hol Chan are required to attend an annual training on coral reef etiquette and best practices, provided by the reserve. Theoretically, Coral Gardens may benefit from the protection of these adjacent coral

reef habitats, as well as the training provided to guides who frequent the area. Herbivore control of macroalgal abundance is robust, reef habitat is conserved, and coral abundance is monitored at these marine reserves, so spawning and recruitment spillover effects could be benefitting Coral Gardens (Williamson et al. 2004). Additionally, concentrated tourism at these locations promotes decreased pressure for Coral Gardens.

This study has documented that *Acropora cervicornis* is *persistent* at Coral Gardens. Whether *A. cervicornis* remains *resilient* at this refugium is less certain. Coral Gardens continued to satisfy most criteria for coral reef refugia reviewed by Kavousi and Keppel (2017, and references therein). Long-term buffering (i.e., favorable environmental conditions persisting for decades to centuries) is key to refugium status, and Coral Gardens meets this criterion. *A. cervicornis* has been present at Coral Gardens for more than several decades (Irwin et al., 2017; Greer et al., 2020) and has persisted despite a decline in live coral cover in the present study period. It likely meets the accessibility criterium (i.e., target taxon can easily reach the site), as the site may benefit from connectivity with two protected areas.

Coral Gardens is the largest documented extant site for *A. cervicornis* in the Caribbean (Busch et al., 2016) that we know of, and larger sites support more adjacent species, larger populations, and greater genetic diversity (Kavousi and Keppel, 2017). This study documents that Coral Gardens also satisfies the important criterium of low exposure to other disturbances (i.e., stressors not related to climate change). It is more difficult to assess whether the site meets the criterium of protection from multiple climatic stressors, particularly warming ocean temperatures, intensified storms, and the increasing threat of ocean acidification. Our data suggest that in the past, *A. cervicornis* at Coral Gardens has been persistent up to the present.

What we cannot predict is how resilient this coral may remain in the face of future climate change, as temperature change appears to be the most likely cause for live coral decline during our study period.

The Future of Coral Gardens

Based on our assessment of potential stressors, we suggest that temperature range, annual mean, and duration of above-average daily temperatures most likely pose the greatest threat to Coral Gardens, assuming status quo for local development factors. Nonetheless, there are many assumptions embedded in this projected future. Any of the known stressors to coral reefs that are not currently an apparent threat to Coral Gardens could be activated. Many of these factors are primarily linked to human development of coastal landscapes. Poorly managed coastal development and infrastructure projects could result in increased sedimentation, pollution, or even nutrient loading, although this is less likely since Coral Gardens is not near potential agricultural sites. While herbivores such as urchins and damselfish are protected in Belize, loss of herbivores to natural causes could result in enhanced macroalgal abundance. Sewage pollution from nearby San Pedro could result in eutrophication. Ocean acidification poses an increasing potential threat to reefs and could affect Coral Gardens in the future. Most importantly, an increase in storm frequency or intensity coupled with other climate change factors could threaten an environment brought to the brink by global and regional warming and/or temperature variability. Managing local stressors at the systems level may help mitigate, delay, or buffer negative change, which highlights the importance of the Northern Belize Coastal Complex in managing marine protected areas and reef connectivity in this region.

Conclusions

Acropora cervicornis coral continues to persist at Coral Gardens despite dramatic losses elsewhere in the Caribbean prior to our study. Our data suggest that Coral Gardens remains a refugium for the species for now, but significant decline during the study period, particularly between 2016 and 2017, threatens this designation. The lack of local stressors may have been key in allowing this exceptional *A. cervicornis* coral reef to thrive, and we found no clear correlation between historical data of storm events and *A. cervicornis* mortality at this site. Although refugia can be an important lifeline for coral species in decline, and may persist for hundreds of years, these habitats are not immune to the effects of *future* climate change. Our data indicate that even in the absence of most reef threats, rising and more variable SSTs may threaten the refugium status of Coral Gardens. We suggest that sub-lethal thermal stress due to higher than normal temperatures, sustained heat, and broader temperature ranges in 2016 may have made *A. cervicornis* at Coral Gardens more vulnerable to other stressors, including the passage of Hurricane Earl, than would have been the case in prior years. With temperatures rising and storms predicted to increase in frequency or duration, we suggest that the future of Coral Gardens as a refugium is uncertain. It seems critical that local stressors remain diminished at this site, but this will not necessarily guarantee sustained live coral abundance. The fate of Coral Gardens, and perhaps similar refugia, will remain increasingly uncertain if storms become more common in concert with rising and more variable temperatures in a warming world.

Acknowledgments

We thank E. Falls, N. Valdez, K. Mattes, and M. Gannon and the Hol Chan Marine Reserve, and F. Cruz at Belize Fisheries Department for logistical and analytical support. We also thank the following students for field and laboratory support: MF White, H Lustig, A Irwin, E Elium, C Walker, A Stier, T W, J Busch, J Abcug, W Benson, D Norvell, S Bender, Z Martin, G Mak, G Garcia Greco, E Peeling, S Dillon, S Holmes, I DeCorte, N An, C Caterham, M Horne, A Mabaka, W Riley, J Villegas, S Walters, R Waters, P Zuñiga, H. Culbertson, R. Abraham.

This work was supported by the National Science Foundation and the Keck Geology Consortium under Grant No. NSF-REU #1358987 to LG, KW and HL and Grant No. NSF-REU #1659322 to LG and KW. Support also was received from The Washington and Lee University: Johnson Opportunity Grant, Summer Research Scholar Program, Lenfest summer research grant, R. Preston Hawkins IV Award in Geology, and Department of Geology to LG, GJ, LM, CS.

References

Aronson RB, Precht WF, Murdoch TJT, Robbart ML. Long-term persistence of coral assemblages on the Flower Gardens Banks, Northwestern Gulf of Mexico: Implications for science and management. Gulf Mexico Sci. 2005b; 23(1):84-94.

Aronson RB, Precht WF. Stasis, biological disturbance, and community structure of a Holocene coral reef. Paleobiology. 1997; 23:326-346.

Aronson RB, Precht WF. White-band disease and the changing face of Caribbean coral reefs. Hydrobiologia. 2001; 460:25-38. doi:10.1023/A:1013103928980

Barker NHL, Roberts M. Scuba diver behaviour and the management of diving impacts on coral reefs. Biological Conservation. 2004; 120(4):481-489. https://doi.org/10.1016/j.biocon.2004.03.021

Bender MA, Knutson TR, Tuleya RE, Sirutis JJ, Vecchi GA, Garner ST et al. Modeled impact of anthropogenic warming on the frequency of intense Atlantic hurricanes. Science. 2010; 327(5964):454-458. DOI: 10.1126/science.1180568

Beven J, (January 21, 2001). Tropical Cyclone Report: Hurricane Keith (Report). National Hurricane Center. Retrieved June 22, 2020. https://www.nhc.noaa.gov/data/tcr/AL152000_Keith.pdf

Beven J (August 4, 2016). Hurricane Earl Tropical Cyclone Update (Report). National Hurricane Center. Retrieved June 22, 2020. https://www.nhc.noaa.gov/archive/2016/al05/al052016.update.08040453.shtml?

Bevan J (March 12, 2018). Hurricane Franklin. National Hurricane Center. Retrieved June 22, 2020. https://www.nhc.noaa.gov/data/tcr/AL072017_Franklin.pdf

Bianucci L, Balaguru K, Smith RW, Leung LR, Moriarty JM. Contribution of hurricane-induced sediment resuspension to coastal oxygen dynamics. Nature Scientific Reports. 2018; 8: 15740. doi:10.1038/s41598-018-33640-3

Birrell CL, McCook LJ, Willis BL, Diaz-Pulido GA. Effects of benthic algae on the replenishment of corals and the implications for the resilience of coral reefs. Oceanography and Marine Biology: an Annual Review. 2008; 46: 25-63.

Brown-Saracino J, Peckol P, Curran HA, Robbart MJ. Spatial variation in sea urchins, fish predators, and bioerosion rates on coral reefs of Belize. Coral Reefs. 2007; 26:71-78.

Busch J, Greer L, Harbor D, Wirth K, Lescinsky H, Curran HA. Quantifying exceptionally large populations of *Acropora* spp. corals in Belize using sub-meter satellite imagery classification. Bull Mar Sci. 2016; 92:265-83.

Carilli JE, Norris RD, Black BA, Walsh SM, McField M. Local stressors reduce coral resilience to bleaching. PLoS ONE. 2009; 4(7): e6324. doi:10.1371/journal.pone.0006324CDIAC. Data available at: http://cdiac.ornl.gov/oceans/doc.html

Clark TR, Roff G, Zhao J, Feng Y, Done TJ, Pandolfi JM. Testing the precision and accuracy of the U-Th chronometer for dating coral mortality events in the last 100 years. Quat. Geochronol. 2014a; 23:35-45.

Clark TR, Zhao J, Roff G, Feng Y, Done TJ, Nothdurft LD et al. Discerning the timing and cause of historical mortality events in modern Porites from the Great Barrier Reef. Geochim Cosmochim Acta. 2014b; 138:57-80.

Cohrs, S (August 22, 2007). U.S. Assessment Team Arrives in Belize Behind Hurricane Dean. American Forces Press Service, News Articles. 2008. Retrieved June 22, 2020. https://web.archive.org/web/20080710231237/http://www.defenselink.mil/news/newsarticle.aspx?id=47139

Consejo, 2020, Hurricanes and Tropical Storms Affecting Belize since 1930. http://consejo.bz/weather/storms.html

Cramer KL, Jackson JBC, Donovan MK, Greenstein BJ, Korpanty CA, Cook GM et al. Widespread loss of Caribbean acroporid corals was underway before coral bleaching and disease outbreaks. Science Advances. 2020; 6(17). Doi: 10.1126/sciadv.aax9395

D’Antonio NL, Gilliam DS, Walker BK. Investigating the spatial distribution and effects of nearshore topography on *Acropora cervicornis* abundance in South-east Florida. PeerJ. 2016; 4:e2473 DOI 10.7717/peerj.2473

Devlin-Durante MK, Miller MM, Caribbean *Acropora* Research Group (Carne L, Smith T, Banaszak AT, Greer L, Irwin A, Fogarty N, Williams D, Precht WF, Baums IB). How old are you? Age estimates in a clonal animal. Molecular Ecol. 2016; 25:5628-5646. doi: 10.1111/mec.13865

Donohue MJ, Boland RC, Sramek CM, Antonelis GA. Derelict fishing gear in the Northwestern Hawaiian Islands: Diving surveys and debris removal in 1999 confirm threat to coral reef ecosystems. Marine Pollution Bulletin. 2001; 42(12): 1301-1312.

Eakin CM, Morgan JA, Heron SF, Smith TB, Liu G, Alvarez-Filip L et al. Caribbean Corals in Crisis: Record Thermal Stress, Bleaching, and Mortality in 2005. PLoS ONE. 2010; 5(11):e13969. doi: 10.1371/journal.pone.0013969

Fabricius KE. Effects of terrestrial runoff on the ecology of corals and coral reefs: review and synthesis. Marine Pollution Bulletin. 2005; 50(2):125-146.

Fang JKH, Mello-Athayde MA, Schonberg CHL, Kline DI, Hoegh-Guldberg O, Dove S. Sponge biomass and bioerosion rates increase under ocean warming and acidification. Global Change Biology. 2013; 19:3581–3591. doi: 10.1111/gcb.12334

Gischler E, Hudson JH. Holocene development of the Belize Barrier Reef. Sedimentary Geology. 2004; 164(3-4):223-236. https://doi.org/10.1016/j.sedgeo.2003.10.006

Gischler E. Pleistocene facies of Belize barrier and atoll reefs. Facies. 2007; 53:27–41. https://doi.org/10.1007/s10347-006-0086-9

Gladfelter EH, Monahan RK, Gladfelter WB. Growth rates of five reef-building corals in the northeastern Caribbean. Bulletin of Marine Science. 1978; 28(4): 728-734.

Gladfelter WB. White-Band Disease in *Acropora palmata*-Implications for the structure and growth of shallow reefs. Bull Mar Sci. 1982; 32:639-43.

Glynn, PW. Coral reef bleaching: facts, hypotheses and implications. Global change biology. 1996; 2(6):495-509.

Glynn PW, D'Croz L. Experimental evidence for high temperature stress as the cause of El Niño coincident coral mortality. Coral Reefs. 1990; 8:181-191.

Greenstein BJ, Curran HA, Pandolfi JM. Shifting ecological baselines and the demise of *Acropora cervicornis* in the western north atlantic and caribbean province: A Pleistocene perspective. Coral Reefs. 1998; 17(3):249-61. doi: 10.1007/s003380050125

Greer L, Jackson JE, Curran HA, Guilderson TP, Teneva L. How vulnerable is *Acropora cervicornis* to environmental change? Lessons from the early to middle Holocene. Geology. 2009; 37:263-66.

Greer L, Clark T, Waggoner T, Busch J, Guilderson TP, Wirth K et al. Coral Gardens Reef, Belize: A refugium in the face of Caribbean-wide *Acropora* spp. decline. PLoS ONE. 2020; 15(9):e0239267.doi.org/10.1371/journal.pone.0239267

Guest JR, Edmunds PJ, Gates R, Kuffner IB, Andersson AJ, Barnes BB et al. A framework for identifying and characterizing coral reef ”oases” against a backdrop of degradation. J Appl Ecol. 2018; 55:2865-2875.

Hoegh-Guldberg O. Climate change, coral bleaching and the future of the world's coral reefs. Marine and Freshwater Research. 1999; 50:839-866.

Hughes TP, Barnes ML, Bellwood DR, Cinner JE, Cumming GS, Jackson JBC et al. Coral reefs in the Anthropocene. Nature. 2017a; 546:82-90.

Irwin A, Greer L, Humston R, Devlin-Durante M, Baums I, Lescinsky H et al. Age and intraspecific diversity of resilient *Acropora* communities in Belize. Coral Reefs. 2017; 36(4):1111-20. doi:10.1007/s00338-017-1602-9

IUCN (International Union for Conservation of Nature), (2017). Belize Barrier Reef Reserve System Conservation Outlook Assessment. The IUCN World Heritage Outlook [website].

<https://worldheritageoutlook.iucn.org/explore-sites/wdpaid/124383>. Finalised 10 November 2017. Accessed 17 August 2020.

Jackson, JBC. Pleistocene Perspectives on Coral Reef Community Structure. Am Zool. 1992; 32(6):719–31.

Kavousi J, Keppel G. Clarifying the concept of climate change refugia for coral reefs. ICES Journal of Marine Science. 2018; 75(1):43-49.

Keck J, Houston RS, Purkis S, Riegl BM. Unexpectedly high cover of *Acropora cervicornis* on offshore reefs in Roatán (Honduras). Coral Reefs. 2005; 24:509. http://dx.doi.org/10.1007/s00338-005-0502-6

Kenkel, C, Meyer E, Matz M. Gene expression under chronic heat stress in populatiosn of the mustard hill coral (Porites astreoides) from different thermal environments. Mol. Ecol. 2013; 22:4322-4334.

Keppel G, Van Niel KP, Wardell-Johnson GW, Yates CJ, Byrne M, Mucina L et al. Refugia: identifying and understanding safe havens for biodiversity under climate change. Global Ecology and Biogeography. 2012; 21(4):393-404.

Lesser MP. Oxidative stress in marine environments: biochemistry and physiological ecology. Annu. Rev. Physiol. 2006; 68:253-278.

Lirman D, Bowden-Kerby A, Schopmeyer S, Huntington B, Thyberg T, Gough M et al. A window to the past: documenting the status of one of the last remaining 'megapopulations' of the threatened staghorn coral *Acropora cervicornis* in the Dominican Republic. Aquat Conserv: Mar and Freshwat Ecosystems. 2010a; 20:773–81.

Louis YD, Bhagooli R, Kenkel CD, Baker AC, Dyall SD. Gene expression biomarkers of heat stress in scleractinian corals: Promises and limitations. Comparative Biochemistry and Physiology, Part C. 2017; 191:63-77.

McCulloch M, Falter J, Trotter J, Montagna P. Coral resilience to ocean acidification and global warming through pH up-regulation. Nature Climate Change. 2012: 2:623-627.

Mesollela KJ. Zonation of uplifted Pleistocene coral reefs on Barbados, West Indies. Science. 1967; 156:638–40.

Mumby, PJ. Bleaching and hurricane disturbances to populations of coral recruits in Belize. Mar Ecol Prog Ser. 1999; 190:27-35.

National Marine Fisheries Service (NMFS). Recovery Plan for Elkhorn (*Acropora palmata*) and Staghorn (*A. cervicornis*) Corals. Prepared by the *Acropora* Recovery Team for the National Marine Fisheries Service, Silver Spring, Maryland. 2015, 167 p.

NOAA (National Oceanic and Atmospheric Administration) National Hurricane Center. NHC Data Archive. Past Track Seasonal Maps. Atlantic Basin 2015. (http://www.nhc.noaa.gov/data/#tracks_all)

NOAA National Centers for Environmental information, Climate at a Glance: Global Time Series, published June 2020, retrieved on June 18, 2020 from https://www.ncdc.noaa.gov/cag/

Nylander-Asplin HF, Hill RL, Doerr JC, Greer L, Fogarty, ND. Population dynamics and genotypic richness of the threatened *Acropora* spp. And their hybrid in the U.S. Virgin Islands. Coral Reefs. 2021. https://doi.org/10.1007/s00338-021-02093-y

O'Leary JK, Potts D, Schoenock KM, McClahanan TR. Fish and sea urchin grazing opens settlement space equally but urchins reduce survival of coral recruits. MEPS. 2013; 493:165-177.

Ott B, Lewis JB. The importance of the gastropod *Coralliophila abbreviata* (Lamarck) and the polychaete *Hermodice carunculata* (Pallas) as coral reef predators. Canadian Journal of Zoology. 1972; 50:1651-1656.

Pandolfi JM, Jackson JBC, Baron N, Bradbury RH, Guzman HM, Hughes TP et al. Are U.S. coral reefs on the slippery slope to slime? Science. 2005; 307(5716):1725-1726. DOI: 10.1126/science.1104258

Pandolfi JM, Jackson JBC. Ecological persistence interrupted in Caribbean coral reefs. Ecol Lett. 2006; 9(7):818-826. doi:10.1111/j.1461-0248.2006.00933.x

Paradis BT, Henry RP, Chadwick NE. Compound effects of thermal stress and tissue abrasion on photosynthesis and respiration in the reef-building coral Acropora cervicornis (Lamarck, 1816). Journal of Experimental Marine Biology and Ecology. 2019; 521:151222.

Precht WF, Aronson RB, Moody RM, Kaufman L. Changing patterns of microhabitat utilization by the threespot damselfish, *Stegastes planifrons*, on Caribbean reefs. PLoS ONE. 2010; 5:e10835. http://dx.doi.org/10.1371/journal.pone.0010835

Precht W, Bruckner A, Aronson R, Bruckner R. Endangered acroporid corals of the Caribbean. Coral Reefs. 2002; 21:41-2.

Precht W, Deslarzes KJP, Hickerson EL, Schmahl GP, Nuttall MF, Aronson R. Back to the future: The history of acroporid corals at the Flower Garden Banks, Gulf of Mexico, USA. Mar. Geol. 2014; 349:152-161.

Purkis SJ, Myint SW, Riegl BM. Enhanced detection of the coral *Acropora cervicornis* from satellite imagery using a textural operator. Remote Sens Environ. 2006; 101:82–94. http://dx.doi.org/10.1016/j.rse.2005.11.009

Riegl B, Purkis SJ, Keck J, Rowlands GP. Monitored and modeled coral population dynamics and the refuge concept. Mar Pollut Bull. 2009b; 58:24–38. http://dx.doi.org/10.1016/j.marpolbul.2008.10.019

Risk MJ. Assessing the effects of sediments and nutrients on coral reefs. Current Opinion in Environmental Sustainability. 2014; 7:108–117.

Roberts CM. Connectivity and management of Caribbean coral reefs. Science. 1997; 278(5342):1454-1457. DOI: 10.1126/science.278.5342.1454

Rosic N, Kaniewska P, Chan C-KK, Ling EY, Edwards D, Dove S et al. Early transcriptional changes in the reef-building coral Acropora aspera in response to thermal and nutrient stress. BMC Genomics. 2014; 15:1052.

Shinn EA. Coral growth-rate: An environmental indicator. Journal of Paleontology. 1966; 40:233-240.

Stough J, Greer L, Benson W. Texture and color distribution-based classification for live coral detection. Proceedings of the 12th International Coral Reef Symposium, Cairns, Australia. 2012; 5.

Stewart SR (Jan 19, 2017) National Hurricane Center Tropical Cyclone Report Hurricane Earl (AL052016) 2 – 6 August 2016 National Hurricane Center, Retrieved June 22, 2020. https://www.nhc.noaa.gov/data/tcr/AL052016_Earl.pdf

van der Zande RM, Achlatis M, Bender-Champ D, Kubicek A, Dove S, Hoegh-Guldberg O. Paradise lost: End-of-century warming and acidification under business-as-usual emissions have severe consequences for symbiotic corals. Global Change Biology. 2020; 26(4):2203-2219. https://doi-org.ezproxy.wlu.edu/10.1111/gcb.14998

Vargas-Ángel B, Thomas JD, Hoke SM. High-latitude *Acropora cervicornis* thickets off Fort Lauderdale, Florida, USA. Coral Reefs. 2003;22:465–73. http://dx.doi.org/10.1007/ s00338-003-0336-z

Wapnick CM, Precht WF, Aronson RB. Millennial-scale dynamics of staghorn coral in Discovery Bay Jamaica. Ecol Lett. 2004; 7(4):354-361.

Weis VM. Cellular mechanisms of Cnidarian bleaching: stress causes the collapse of symbiosis. J. Expt. Biol. 2008; 201:3059-3066.

Ysaguirre W (2008-06-01). Tropical Storm Arthur hits Yucatan. FOX News. Associated Press. 2008, Retrieved June 22, 2020. https://www.foxnews.com/story/tropical-storm-arthur-hits-yucatan

Figure 1

Click here to access/download;Figure;Fig 1.tif

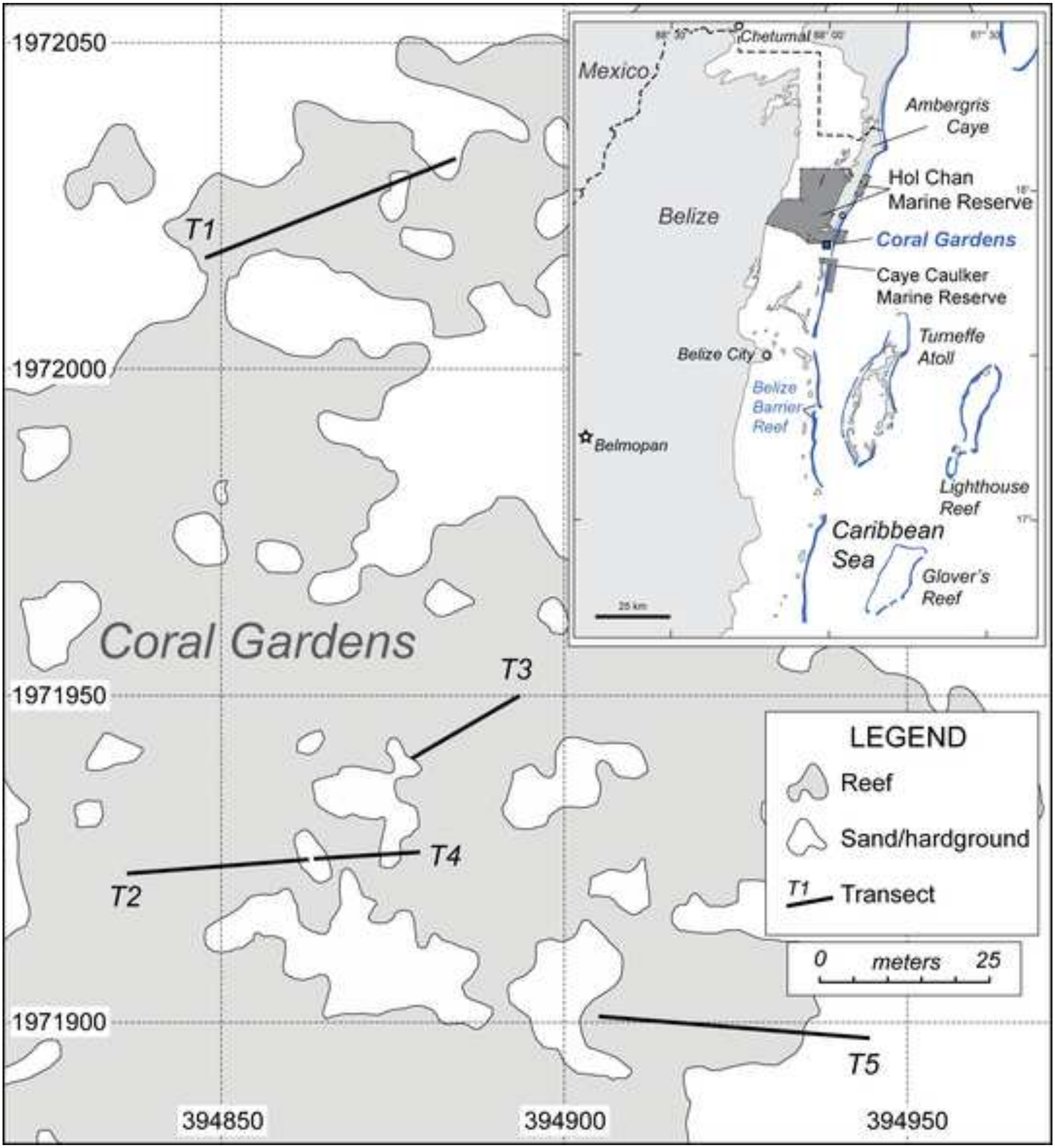

Figure 2

Click here to access/download;Figure;Fig 2.tif

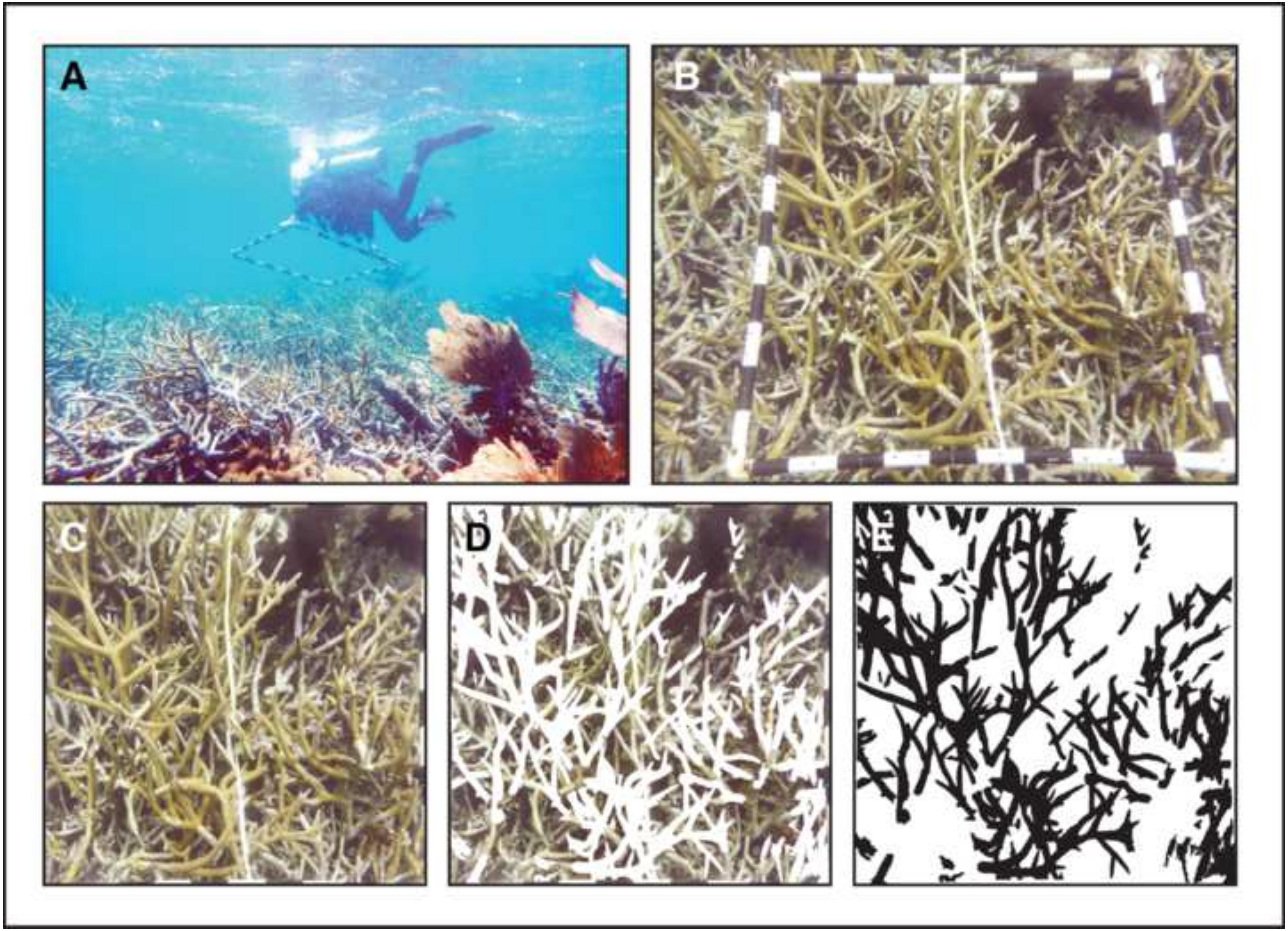

Figure 3

Click here to access/download;Figure;Fig 3.tif

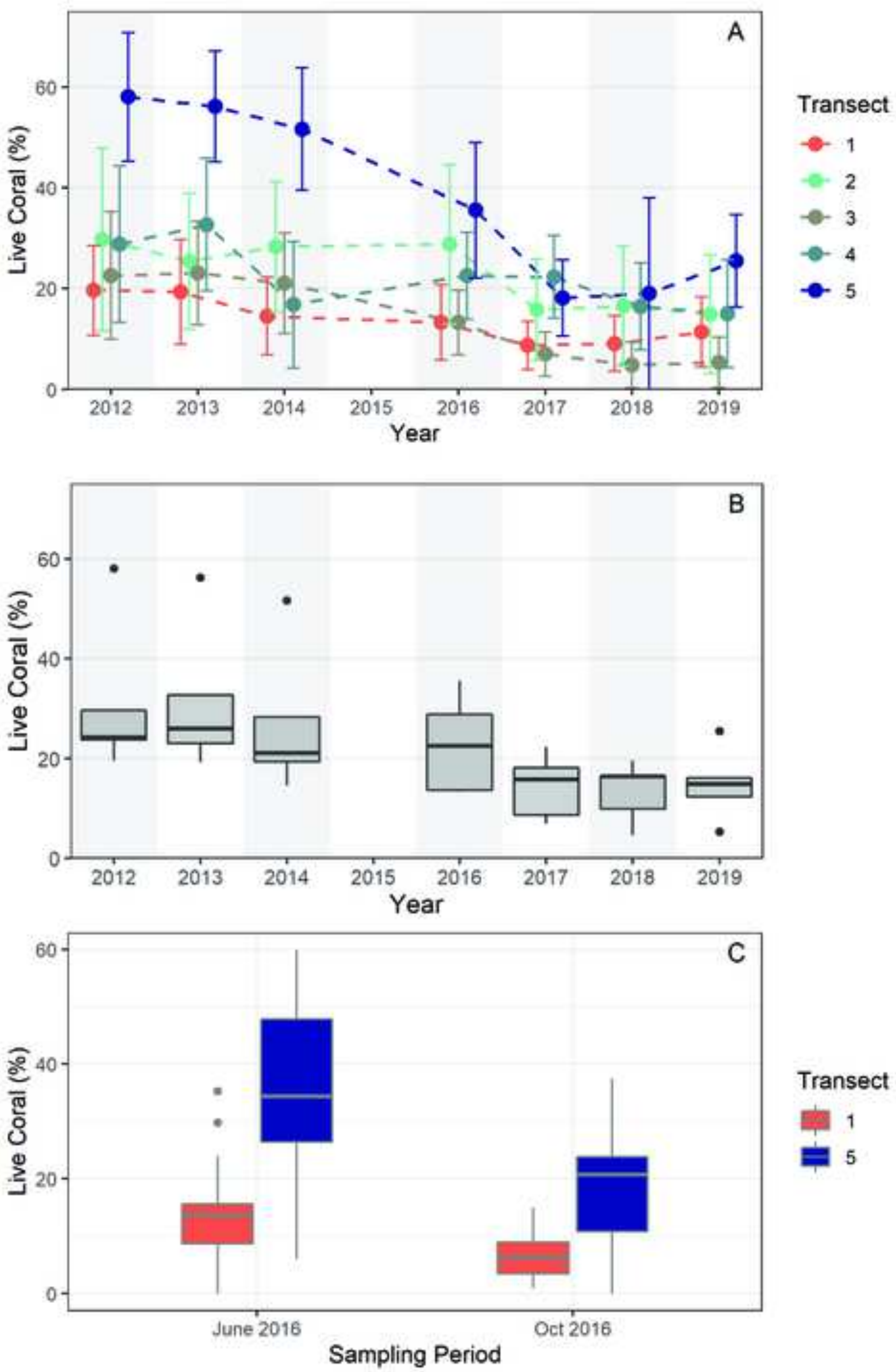

Figure 4

Click here to access/download;Figure;Fig 4.tif

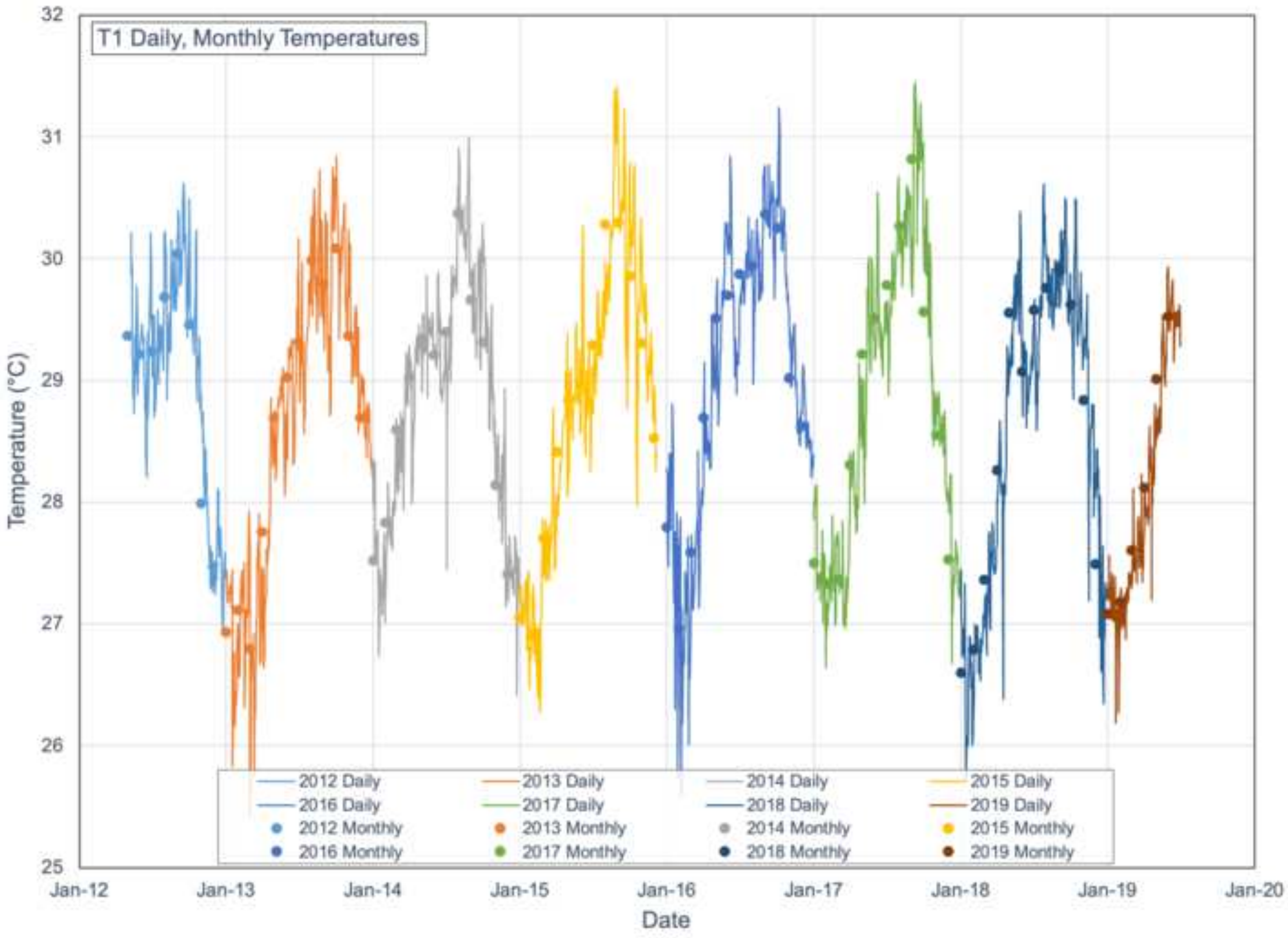

Figure 5

Click here to access/download;Figure;Fig 5.tif

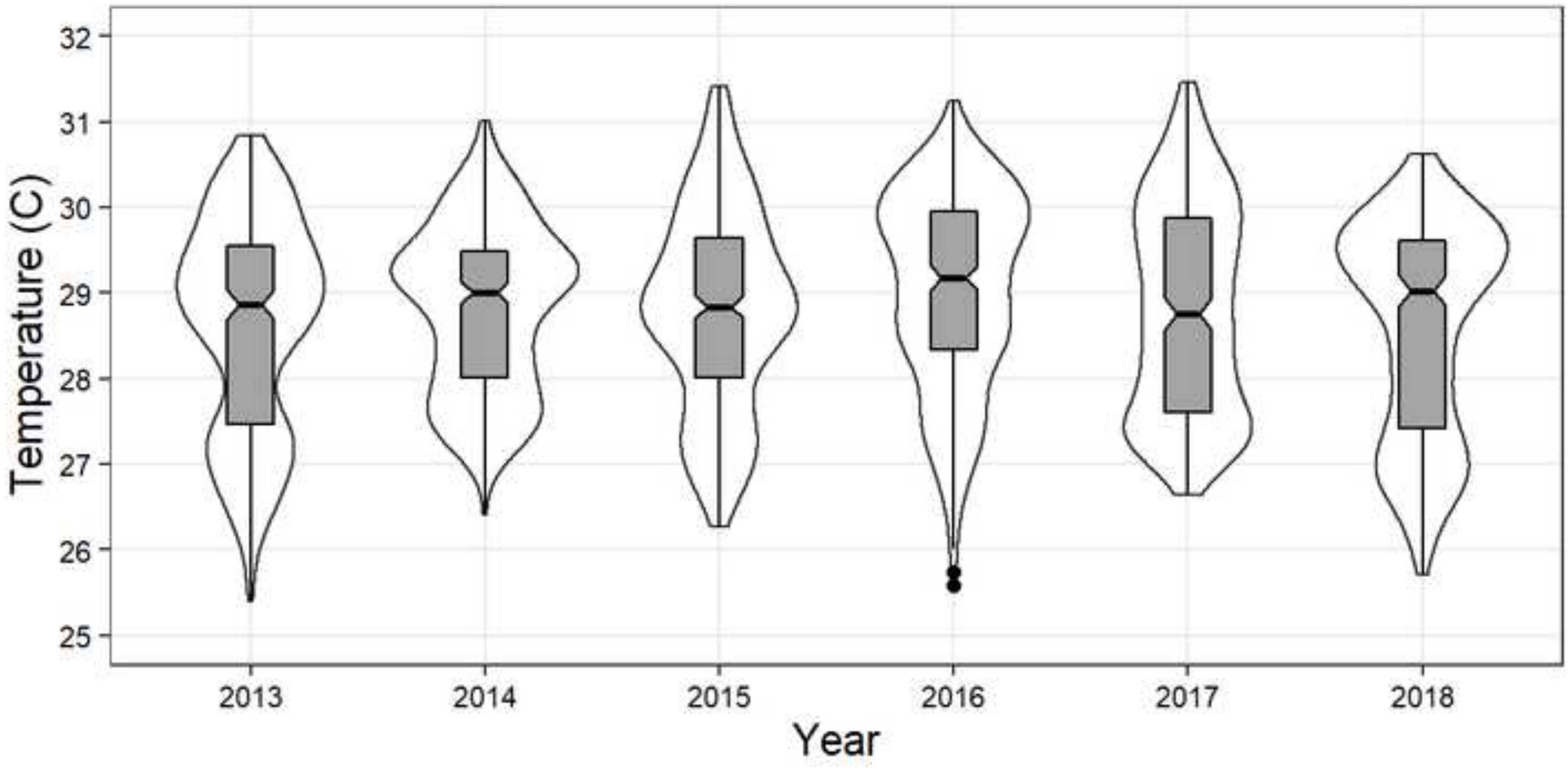

Figure 6a

Click here to access/download;Figure;Fig 6a.tif

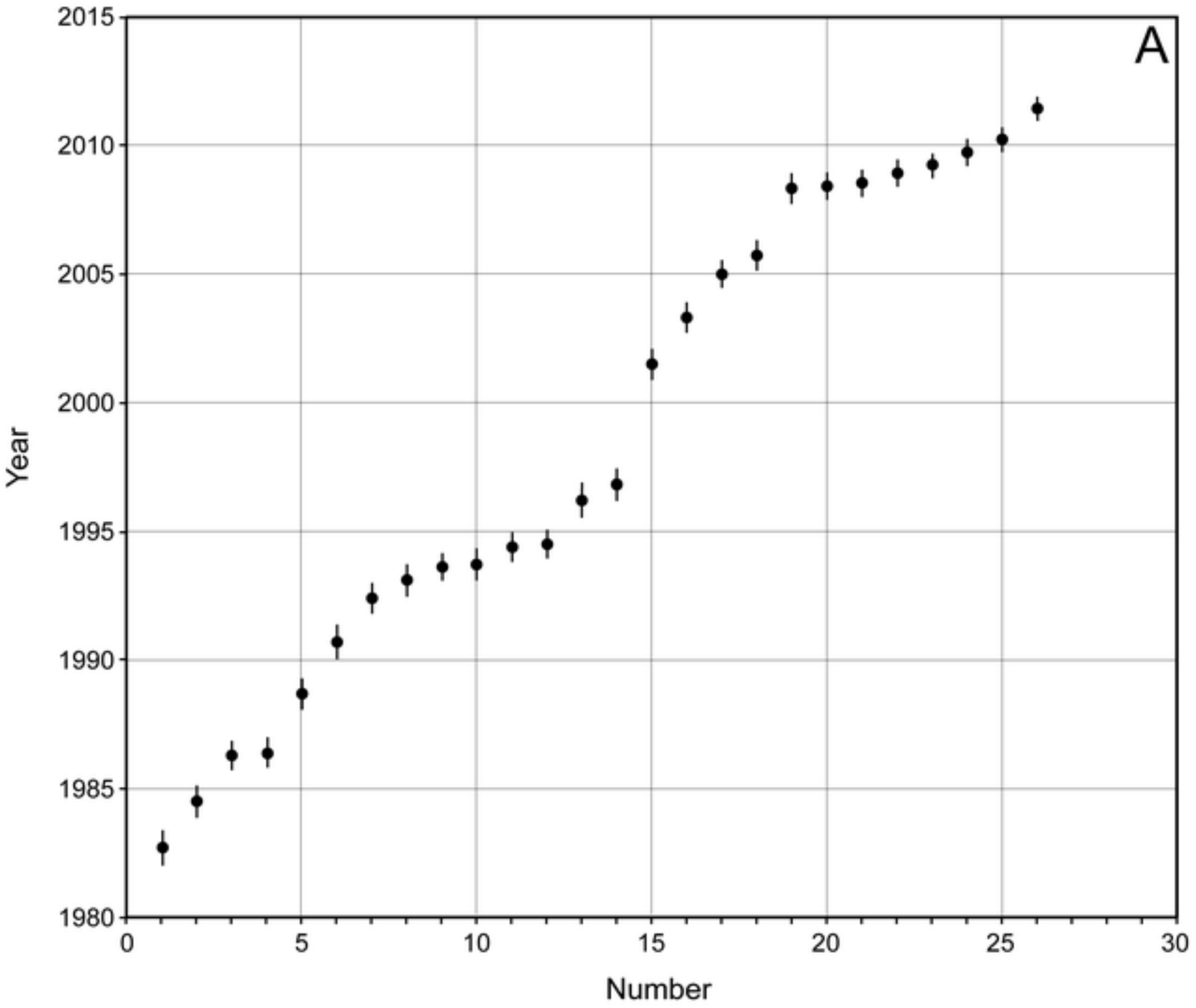

Figure 6b

Click here to access/download;Figure;Fig 6b.tif

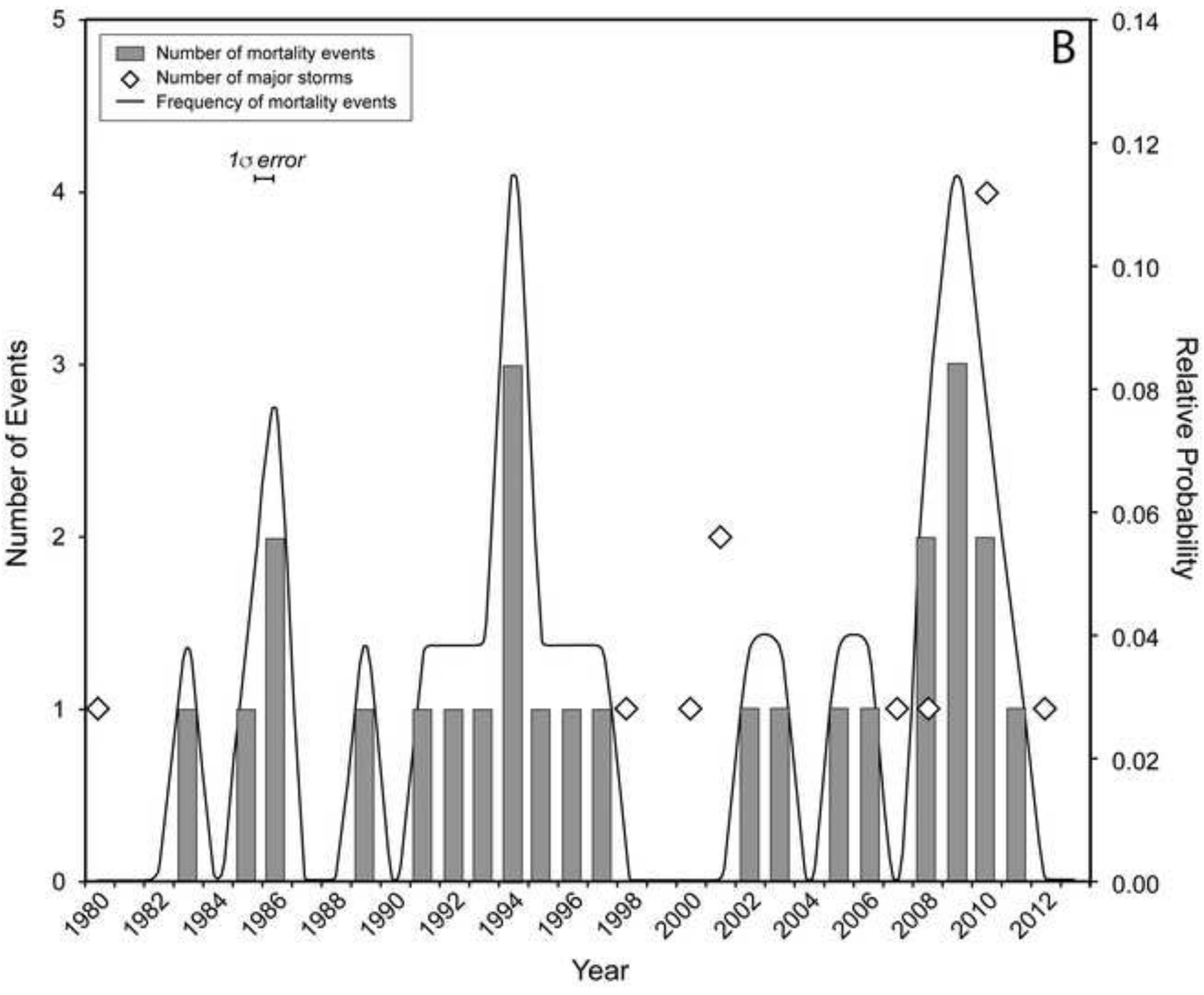

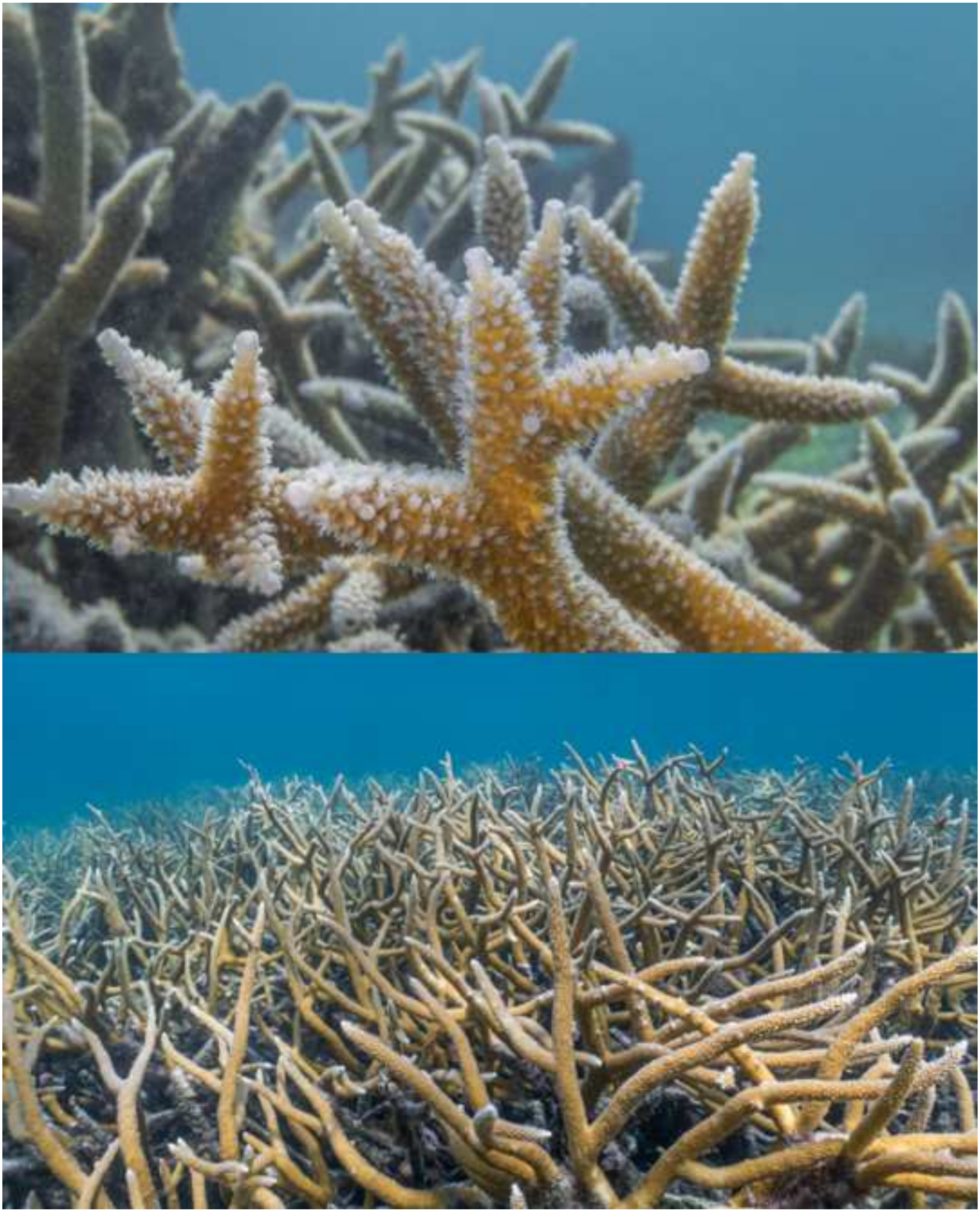

Supplement: Attachment — Submitted filename: PONE-D-22-24875_reviewer 2.pdf [file pone.0280852.s010.pdf]
