# Supplementary material for: Coral Gardens Reef, Belize: An Acropora spp. refugium under threat in a warming world
Source: PLoS One. 2023 Feb 8;18(2):e0280852. doi: 10.1371/journal.pone.0280852 (PMC9907857; doi:10.1371/journal.pone.0280852)
Supplement: Attachment — Submitted filename: Response to Reviewers.pdf [file pone.0280852.s011.pdf]

WASHINGTON AND LEE
UNIVERSITY

DEPARTMENT OF EARTH AND ENVIRONMENTAL GEOSCIENCE

Emily Chenette, Editor-in-Chief, *PLOS ONE*
James Guest, Academic Editor *December 4, 2022*

Dear Drs. Chenette and Guest,

Below is our response to reviewer and editor comments. We appreciate the feedback and feel we now submit a far better manuscript than the first submission. Our comments are in **bold**.

PONE-D-22-24875
Coral Gardens Reef, Belize: An Acropora spp. refugium under threat in a warming world
PLOS ONE

We look forward to receiving your revised manuscript.

1. When submitting your revision, we need you to address these additional requirements.
   Please ensure that your manuscript meets PLOS ONE's style requirements, including those for file naming.

This has been corrected.

2. Thank you for stating the following in the Acknowledgments Section of your manuscript:
"This work was supported by the National Science Foundation and the Keck Geology Consortium under Grant No. NSF-REU #1358987 to LG, KW and HL and Grant No. NSF-REU #1659322 to LG and KW. Support also was received from The Washington and Lee University: Johnson Opportunity Grant, Summer Research Scholar Program, Lenfest summer research grant, R. Preston Hawkins IV Award in Geology, and Department of Geology to LG, GJ, LM, CS."

We note that you have provided funding information that is not currently declared in your Funding Statement. However, funding information should not appear in the Acknowledgments section or other areas of your manuscript. We will only publish funding information present in the Funding Statement section of the online submission form.

Please remove any funding-related text from the manuscript and let us know how you would like to update your Funding Statement. Currently, your Funding Statement reads as follows:

"This work was supported by the National Science Foundation and the Keck Geology Consortium under Grant No. NSF-REU #1358987 to LG, KW and HL and Grant No. NSF-REU #1659322 to LG and KW (https://keckgeology.org/). Support also was received from The Washington and Lee University: Johnson Opportunity Grant, Summer Research Scholar Program, Lenfest summer research grant, R. Preston **Hawkins** IV Award in Geology, and Department of Geology to LG, GJ, LM, CS, and ARC DECRA Fellowship support for TC (https://www.arc.gov.au/grants/discovery-

Founded in 1749

204 W. Washington Street | Lexington, VA 24450-2116
(540) 458-8800 | Fax (540) 458-8142 | go.wlu.edu/geoscience

program/discovery-early-career-researcher-award-decra). The funders had no role in study design, data collection and analysis, decision to publish, or preparation of the manuscript."

Please include your amended statements within your cover letter; we will change the online submission form on your behalf.

We apologize and have removed the acknowledgment section from the manuscript and wish to keep the statement above originally submitted in our funding statement with one correction above -the correct spelling is Hawkins (and *not* Hawkinas). We have included the statement in our cover letter.

3. We noted in your submission details that a portion of your manuscript may have been presented or published elsewhere.

"We do call upon previously aged 230Th data presented in Greer et al. (2020) but we apply the data uniquely to this manuscript."

Please clarify whether this [conference proceeding or publication] was peer-reviewed and formally published. If this work was previously peer-reviewed and published, in the cover letter please provide the reason that this work does not constitute dual publication and should be included in the current manuscript.

Please see our cover letter for clarification.

4. Please include your full ethics statement in the 'Methods' section of your manuscript file. In your statement, please include the full name of the IRB or ethics committee who approved or waived your study, as well as whether or not you obtained informed written or verbal consent. If consent was waived for your study, please include this information in your statement as well.

Our ethics statement was in a later section of the paper. It has been moved to the methods section.

5. We note that Figure 1 in your submission contain [map/satellite] images which may be copyrighted. All PLOS content is published under the Creative Commons Attribution License (CC BY 4.0), which means that the manuscript, images, and Supporting Information files will be freely available online, and any third party is permitted to access, download, copy, distribute, and use these materials in any way, even commercially, with proper attribution. For these reasons, we cannot publish previously copyrighted maps or satellite images created using proprietary data, such as Google software (Google Maps, Street View, and Earth).

Upon inquiry, it is our understanding that since a similar form of our hand-drawn Location Maps (Figure 1) were published in PLOS ONE we are able to present our Figure 1 as is: see author inquiry and response from PLOS ONE Publications Assistant Gena Louque ('Regarding your concern about the map, if the basemap was from any source that was previously copyrighted under anything more restrictive than CC BY 4.0, we will need permission from the copyright holder in order to publish it in PLOS ONE. As the map in Figure 1C was published

WASHINGTON AND LEE
UNIVERSITY

DEPARTMENT OF EARTH AND ENVIRONMENTAL GEOSCIENCE

under PLOS ONE, no permission is needed. The only requirement is that the original source is cited.' Case Number: 07776129 ref:_00DU0Ifis._5004P22uXxA:ref

We now cite the earlier PLOS paper in the figure caption.

6. We note that Figures 2 and 7 in your submission contain copyrighted images. All PLOS content is published under the Creative Commons Attribution License (CC BY 4.0), which means that the manuscript, images, and Supporting Information files will be freely available online, and any third party is permitted to access, download, copy, distribute, and use these materials in any way, even commercially, with proper attribution.

We require you to either (1) present written permission from the copyright holder to publish these figures specifically under the CC BY 4.0 license, or (2) remove the figures from your submission:

The photographs in Figures 2 and 7 were taken by the authors of this manuscript and are not under copyright.

Additional Editor Comments: I received two sets of reviews that identify some issues with the manuscript. Both reviewers agreed that the manuscript was of great value, however one reviewer did notice an issue with the calculation of DHWs. I agree with them on this point and suggest that you look at using a more relevant climatology for calculating MMM. This may change some of your results and interpretation, so I've suggested major revision.

We are grateful for the references and guidance in rigorously calculating DHWs and absolutely agree with our reviewer's suggestions. In revising our methodology, we find even stronger support for our idea that anomalous temperatures may have pre-conditioned Coral Gardens for live coral loss associated with Hurricane Earl in 2016.

Please see our revised methods, results, and discussion sections pertaining to temperature in the revised manuscript. In sum, we first compared our sensor data with the 0.5-deg gridded SST data (*CoralTemp v3.1*) from the Coral Reef Watch 5-km product suite (Heron et al., 2015) and found them to highly correlated. We then calculated the maximum monthly means (MMM) SST climatology for Coral Gardens using the gridded data for 1985-1990 + 1993 as suggested by our reviewer. We calculated DHW as daily values that exceeded MMM + 1°C over a 12-week period.

[Note: HTML markup is below. Please do not edit.]

Reviewers' comments:
Reviewer's Responses to Questions

Comments to the Author

Founded in 1749

204 W. Washington Street | Lexington, VA 24450-2116
(540) 458-8800 | Fax (540) 458-8142 | go.wlu.edu/geoscience

1. Is the manuscript technically sound, and do the data support the conclusions?
The manuscript must describe a technically sound piece of scientific research with data that supports the conclusions. Experiments must have been conducted rigorously, with appropriate controls, replication, and sample sizes. The conclusions must be drawn appropriately based on the data presented.
Reviewer #1: Yes

2. Has the statistical analysis been performed appropriately and rigorously?
Reviewer #1: No

Please see our response to the Editor above for our revised methodology for calculating MMM and DHW.

3. Have the authors made all data underlying the findings in their manuscript fully available? The PLOS Data policy requires authors to make all data underlying the findings described in their manuscript fully available without restriction, with rare exception (please refer to the Data Availability Statement in the manuscript PDF file). The data should be provided as part of the manuscript or its supporting information, or deposited to a public repository. For example, in addition to summary statistics, the data points behind means, medians and variance measures should be available. If there are restrictions on publicly sharing data—e.g. participant privacy or use of data from a third party—those must be specified.
Reviewer #1: No

We now include 9 supplementary data files.

4. Is the manuscript presented in an intelligible fashion and written in standard English? PLOS ONE does not copyedit accepted manuscripts, so the language in submitted articles must be clear, correct, and unambiguous. Any typographical or grammatical errors should be corrected at revision, so please note any specific errors here.
Reviewer #1: Yes

5. Review Comments to the Author

Reviewer #1: This manuscript describes an assessment of threats to one of the largest remaining healthy Acropora cervicornis patches in the Caribbean. Previous work by the lead author has identified this field site as a refugium of Acropora spp. and this paper expands that work with many years of intensive field monitoring and a variety of types of ancillary data to analyze the potential stability of the refugium. The manuscript is clearly written and generally a valuable contribution to the field. It is important for future conservation of endangered species to recognize refugia and recognize the potential threats that could destroy them and the species they protect.

In my review submission, I indicate that the data are not available because I could not find a supplement and the data were not all in the manuscript. There were summary tables in the text, but not all of the raw data. I would have expected a table of daily and monthly temperature data, as well as all of the live coral abundance data for each transect, the pH data, the substrate composition data,

sediment size and composition data, canopy height, sediment thickness and depth to hardground, urchin counts, and damselfish survey data.

We now include 9 supplementary data files.

I have one main concern with the analysis as it is written. The authors use the concept of degree heating weeks, which has been used for many years to quantify coral bleaching risk. However, the calculations described in the present text indicate incorrect application of the concept, according to my understanding. The issue is that DHW is supposed to be relative to the mean maximum monthly temperature (MMM) during a base period when bleaching was not common. Using the monthly data for each year during the study period, 2012-2020, implicitly assumes instantaneous adaptation of coral populations to current warming trends, which is a bad assumption. NOAA coral reef watch, which has popularized this metric uses a base period of 1985–1990+1993 for historic reasons (see Heron et al 2015). I recommend using the same base period.

Please see our response to the Editor above for our revised methodology for calculating MMM and DHW.

I understand that a problem with the 1980s-1990s base period is that you don't have local in-situ data as you do for the 2012-2020 period. However global gridded SST products are widely available and often track local reef temperatures fairly well (Winter et al, 1998). A calibration of the monthly in-situ data with gridded data during the period of common data (as in Smith et al., 2006) will permit you to adjust the available gridded temperature to better match the expected larger variance of a local record during the earlier base period. I think this is will be a much better estimate of MMM against which to calculate DHW.

Please see our response to the Editor above for our revised methodology for calculating MMM and DHW.

Another potential issue with the DHW concept as applied is that recent work (Lachs et al, 2021) indicates that a lower threshold of MMM+0 for 8 weeks duration may better capture thermal stress in corals. I am ambivalent about the need to use this lowered threshold as I agree with Lachs et al., 2021 that spatial variations in coral response are likely significant. However, I think you will find that by using a more reasonable base period for the climatological MMM, you will have substantially more accumulation of DHW during the course of the study, especially 2016.

We calculated DHW's using both methods and the results were similar in annual comparisons, just with greater numbers of DHW's resulting from the Lachs et al. (2021) method. We choose to go with only the original method that is better established and widely used as a measure of DHW.

Some specific comments for small corrections and a list of the references cited above follow.

SPECIFIC COMMENTS

Line 117 The description "high resolution" is very unclear for multiple reasons. Is the resolution over space or time, and how high is "high"? The compound adverb (which needs to be written "high-resolution" to be correct) describes the calculations, but I don't think the calculations had high resolution, instead I think the spatial data used for the calculations were of cm-scale resolution. To a microscopy specialist, cm-scale is exceedingly low resolution. Further the syntax using "high-resolution" first makes a reader wonder if all the data in the list is "high-resolution", or if the authors are only referring to the calculations of live coral cover.

We agree and have changed 'high resolution calculations' to 'measurements', which more accurately reflects our intent.

Line 119. I would argue that all of the data in this list are observational data. Perhaps the authors mean "personal observations" of conditions at the site over the years.

We agree and have re-phrased as 'personal observations in the field'.

Figure 1: This figure would be easier to see as a two-panel figure. The inset map is very small and highly detailed, yet the base map has little fine detail and is rather large. They would both be more appropriately sized if they were equal and side-by-side.

We have now separated the figure into 2 panels.

Figure 2: the caption describes a m2 quadrat, but I think this would be better described as a 1 m2 quadrat. Additionally, the caption describes the coral image traced with Adobe Photoshop, but in the text, Adobe Illustrator is attributed. This should be consistent.

We agree and have changed the caption to read 1 m^2 quadrat and have corrected Photoshop to Illustrator.

REFERENCES

Heron, S.F.; Liu, G.; Eakin, C.M.; Skirving, W.J.; Muller-Karger, F.E.; Vera-Rodriguez, M.; de la Cour, J.L.; Burgess, T.F.R.; Strong, A.E.; Geiger, E.F.; et al. Climatology Development for NOAA Coral Reef Watch's 5-km Product Suite, NOAA Technical Report; NESDIS: Silver Spring, MD, USA, 2015; Volume 145.

Lachs, L.; Bythell, J.C; East, H.K; Edwards, A.J; Mumby, P.J; Skirving, W.J; Spady, B.L; Guest, J.R. Fine-Tuning Heat Stress Algorithms to Optimise Global Predictions of Mass Coral Bleaching. Remote Sens. 2021, 13, 2677. https://doi.org/10.3390/rs13142677

Smith, J. M., Quinn, T. M., Helmle, K. P., and Halley, R. B. (2006), Reproducibility of geochemical and climatic signals in the Atlantic coral Montastraea faveolata, Paleoceanography, 21, PA1010, doi:10.1029/2005PA001187.

A. Winter, R. S. Appeldoorn, A. Bruckner, E. H. Williams. Jr., C. Goenaga, Sea surface temperatures and coral reef bleaching off La Parguera, Puerto Rico (northeastern Caribbean Sea). Coral Reefs (1998) 17 : 377-382.

We appreciate the references, all have been useful!

Reviewer #2:
See attached PDF of paper - see comments in drop down tabs
Summary

1. accept manuscript with revision

2. limitation of study - one field visit (snapshot) per year - hard to infer causality for the intervening 11 months Need to discuss this limitation

We agree that our study is limited by the realities of field season limitations and the time it takes to work with the annual data we do have. In our original manuscript We have included additional references to this limitation (lines 427, 542-544 as well as 573).

3. No repeated measures of coral colonies during period of loss (mortality) –

See response to item 2 above. The reviewer correctly points out that 'Major problem is collection period limited observations to June. Most stress related to temperature including paling, bleaching, increased WBD prevalence, RTL/shutdown reaction are generally observed on Caribbean reefs from July through October.' We agree and have tried to clarify our study limitations in the revised manuscript.

The reviewer also stated 'I disagree that WBD cannot be distinguished from bleaching without histopathology.' We have corrected to say that we personally could not discern. I wish we had the expertise of the reviewer in the field!

4. Can only guess as to the cause of mortality ???

We hope that we have better clarified our proposed causes with the new temperature, MMM, and DHW calculations.

5. Agree that temperature is likely proximal driver of ACER loss

204 W. Washington Street | Lexington, VA 24450-2116
(540) 458-8800 | Fax (540) 458-8142 | go.wlu.edu/geoscience

6. Personal observation to my one visit to site = There is also a minor level of background WBD at Coral Gardens (~1%) - this could have jumped as temperatures reached their maximum in late summer /early fall causing a localized but temporally limited mortality event.

This is most certainly possible. I have not observed bleaching of *Acropora cervicornis* at Coral Gardens in my 16 visits since 2011 (although I have rarely been there late summer) but I have observed bleaching of other massive corals. My colleague and co-author Kirah Forman-Castillo who works at the Hol Chan Marine Reserve has not observed bleaching of acroporids at Coral Gardens either, but has seen some rare instances of paling nearby.

see the attached references

Randall, C.J. and van Woesik, R., 2015. Contemporary white-band disease in Caribbean corals driven by climate change. *Nature Climate Change*, *5*(4), pp.375-379.

Gignoux-Wolfsohn, S.A., Precht, W.F., Peters, E.C., Gintert, B.E. and Kaufman, L.S., 2020. Ecology, histopathology, and microbial ecology of a white-band disease outbreak in the threatened staghorn coral Acropora cervicornis. *Diseases of Aquatic Organisms*, *137*(3), pp.217-237.

We appreciate the references. The reviewer suggests that one of our references is in conflict with those above (Carilli et al., 2009) but we are choosing to leave it in the manuscript. It has been well cited and was published in PLOS ONE.

Just as easy to infer that disease/bleaching was as important as passage of hurricane

We address this concern well in the revised draft and suggest that temperature may be critical to the loss of live coral during the study period in concert with the passage of the storm.

Hurricane wave stress plus temperature stress may have led to shut down reaction (speculation on my part - but possible)

see SDR

Antonius, A., 1977. Coral mortality in reef: a problem for science and management. In: Proc. 3rd Int. Coral. Reef. Symp, 2, pp. 618–623.

We believe this comment is related to our text 529-532 in the original submission that reads 'Tropical storm and hurricane sediment mobilization may also cause abrasion even when the structural foundation is maintained. In addition to sediment suspension, we have observed that even when architectural integrity remains sound, loose branches may move and touch each other during high wave activity. Under optimal temperatures corals may be able to heal, but at higher temperatures they might not.' The reviewer writes 'This could be a form of SDR see Antonius A (1977) Coral mortality in reefs: a problem for science and management. Proc 3rd Int Coral Reef Symp 2: 618–623. Would like to see visual observations confirming'. Here the reviewer refers to

WASHINGTON AND LEE
UNIVERSITY

DEPARTMENT OF EARTH AND ENVIRONMENTAL GEOSCIENCE

the phenomenon of Shut Down Reaction mentioned by Antonius (1977). A 2012 reference to this work (Richardson, 2012) defines this as follows:

***Shut Down Reaction*: In 1977 Antonius first described the coral disease he termed shut down reaction (SDR). He noted that this disease only occurred in corals maintained in aquaria (Antonius 1977), an observation that holds true today (Sutherland *et al.* 2004). The name is based on the extremely rapid rate of tissue destruction, 10 cm per hour along a moving front, that can be transmitted using infected tissue or contact with an infected colony, but only when the recipient colony is under stress (Antonius 1981b). No definitive work has been carried out to define the etiology of SDR.**

Unfortunately, we do not have a visual from Coral Gardens and mean this to be a more speculative statement.

Richardson, LL, 2012, Arnfried Antonius, coral diseases, and the AMLC, Revista de Biologia Tropical, vol. 60, suppl.1 *On-line version* ISSN 0034-7744

9. Manuscript is well written, stats ok, good use of references

10. Broad implication for linking of climate change science with coral loss

My co-authors and I look forward to your review of our manuscript revision. Thank you for your time and consideration.

Sincerely,

Lisa Greer, Professor of Geology
Department of Earth and Environmental Geoscience
Washington and Lee University
greerl@wlu.edu (540) 460-3145
